# Supplementary material for: Effects and utility of an online forward triage tool during the SARS-CoV-2 pandemic: a mixed method study and patient perspectives, Switzerland
Source: BMJ Open. 2022 Jul 11;12(7):e059765. doi: 10.1136/bmjopen-2021-059765 (PMC9274020; doi:10.1136/bmjopen-2021-059765)
Supplement: Supplementary data [file bmjopen-2021-059765supp001.pdf]

Supporting information OFTT Questionnaire and results

Supporting information 1. Usage of online tools during the COVID-19 pandemic: email survey

v1	**How did you get to this Online - Tool?**		
	1 - My family doctor advised me to use this tool.	9	5.1%
	2 - I found the information on the Internet.	113	64.2%
	3 - The website was recommended to me by family/friends.	17	9.7%
	4 - Via a telephone hotline.	1	0.6%
	5 - Other (Free text)	35	19.9%
v2	**Did you find the information that you needed?**		
	1 - Yes	154	87.5%
	2 - No, because the information was not comprehensive.	17	9.7%
	3 - No, because the information was not clear.	5	2.8%
v3	**What information did you search for? I wanted....**		
	1 - ... more information on COVID-19 symptoms	97	55.1%
	2 - ... more information on how to cope with symptoms	4	2.3%
	3 - ... to know when to consult a doctor	36	20.5%
	4 - ... to know more on testing criteria	32	18.2%
	5 - ... to know where to test	7	4.0%
	6 - Other	-	-
v4	**Did the online tool recommend you to test for COVID-19?**		
	1 - Yes	56	31.8%
	2 - No	120	68.2%
v5	**Did you stick to the recommendations?**		
	1 - Yes	149	84.7%
	2 - No	27	15.3%
v6	**If you followed the Online - Tool recommendations, what made you do so?**		
	1 - I trust the website as a reliable information source.	60	34.1%
	2 - I compared the recommendations with recommendations from the media and took a decision.	20	11.4%
	3 - I compared the recommendations with those from FOPH (BAG) and took a decision.	53	30.1%
	4 - I sought advice from a person I trusted.	7	4.0%
	5 - Other, please specify: Free text	9	5.1%
v7	**In case you did not follow the recommendations, why did you not**		
	1 - I did not trust the website as a reliable source of information.	1	0.6%
	2 - The recommendations from the website differed from the media recommendations.	2	1.1%
	3 - I feared for my life and needed to consult a GP in person.	6	3.4%
	4 - Other, please specify: Free text	18	10.2%
v8	**Were your fears and anxieties allayed after visiting the website?**		
	1 - Yes, the information from the website reassured me.	73	41.5%
	2 - No, the information from the website did not reassure me.	13	7.4%

	3 - No, the information from the website increased my fears and anxieties.	6	3.4%
	4 - I was not worried.	84	47.7%
v9	**How did you cope with your fears? What helped you cope?**		
	1 - Free text	-	-
v10	**In case you went to the GP, did you call ahead of time to notify them of your visit?**		
	1 - Yes	115	65.3%
	2 - No	61	34.7%
v11	**Did you get tested for Coronavirus (SARS-CoV-2 Swab)?**		
	1 - Yes	48	27.3%
	2 - No	128	72.7%
v12	**What was the result?**		
	1 - Positive	3	1.7%
	2 - Negative	45	25.6%
v13	**How did the media influence your decision making? The**		
	1 - ... helpful	81	46.0%
	2 - ... confusing	47	26.7%
	3 - I do not rely on the media as an information source.	25	14.2%
	4 - Free text	23	13.1%
v14	**How old are you?**	Mean 50.5 (SD 15), range 18-82	
v15	**What is your sex?**		
	1 - Female	101	57.4%
	2 - Male	75	42.6%
	3 - Other	0	0.0%
v16	**What is your nationality?**		
	0 - Missing	0	0.0%
	1 - Swiss	147	83.5%
	2 - German	13	7.4%
	3 - French	1	0.6%
	4 - Italian	3	1.7%
	5 - Liechtenstein	0	0.0%
	6 - Greater Europe	4	2.3%
	7 - Free text	7	4.0%
v17	**In which province do you live?**		
	1 - Bern	108	61.4%
	2 - Zürich	12	6.8%
	3 - Luzern	10	5.7%
	4 - Uri	0	0.0%
	5 - Schwyz	1	0.6%
	6 - Obwalden	0	0.0%
	7 - Nidwalden	0	0.0%
	8 - Glarus	0	0.0%
	9 - Zug	2	1.1%
	10 - Fribourg	7	4.0%
	11 - Solothurn	3	1.7%
	12 - Basel-Stadt	2	1.1%
	13 - Basel-Landschaft	1	0.6%
	14 - Schaffhausen	0	0.0%

	15 - Appenzell Ausserrhoden	2	1.1%
	16 - Appenzell Innerrhoden	0	0.0%
	17 - St. Gallen	2	1.1%
	18 - Graubünden	3	1.7%
	19 - Aargau	9	5.1%
	20 - Thurgau	1	0.6%
	21 - Ticino	2	1.1%
	22 - Vaud	7	4.0%
	23 - Valais	0	0.0%
	24 - Neuchâtel	1	0.6%
	25 - Geneva	0	0.0%
	26 - Jura	0	0.0%
	27 - I do not live in Switzerland	3	1.7%
v18	**What is your highest level of education?**		
	0 - Missing	6	3.4%
	1 - Tertiary education (university degree, college of education)	120	68.2%
	2 - Upper secondary education (High School Graduation, FMS, EZF, EBA)	27	15.3%
	3 - Lower secondary education/ obligatory schooling completed	23	13.1%
	4 - No formal education		
v19	**Are you currently...**		
	0 - Missing	33	18.8%
	1 - Employed	106	60.2%
	2 - Self employed	24	13.6%
	3 - Unemployed already before the current pandemic	3	1.7%
	4 - I lost my job during the COVID-19 period	1	0.6%
	5 - Studying or in an apprenticeship	9	5.1%
v20	**How much approximately do you earn per month? (net income in December 2019 including 1/12 of the 13th month salary.)**		
	0 - Missing	29	16.5%
	1 - Less that 4'000 CHF	26	14.8%
	2 - Between 4'001 und 6'000 CHF	42	23.9%
	3 - Above 6'001 CHF	79	44.9%
v21	**What type of health insurance do you have?**		
	1 - General	68	38.6%
	2 - Telemedicine - Modell	12	6.8%
	3 - GP - Modell	83	47.2%
	4 - Another alternative model	8	4.5%
	5 - No insurance	5	2.8%
v22	**In a second stage, we will interview individual participants of**		
	1 - Yes, I consent to be contacted.	78	44.3%
	2 - No, please, no more interviews.	98	55.7%
	3 - Free text	-	-
