# Supplementary material for: Effects and utility of an online forward triage tool during the SARS-CoV-2 pandemic: a mixed method study and patient perspectives, Switzerland
Source: BMJ Open. 2022 Jul 11;12(7):e059765. doi: 10.1136/bmjopen-2021-059765 (PMC9274020; doi:10.1136/bmjopen-2021-059765)
Supplement: Supplementary data [file bmjopen-2021-059765supp002.pdf]

Interview Guide: Coronatest.ch -Patients v 2

Rapport

0Describe yourself (prompts; nationality, occupation, living arrangements, employment status)

Accessibility

0How did you get to coronatest.ch website? (referred by …, online search). What is your understanding of an OFTT?

0Did you consult BAG- was the information supplied by BAG understandable to you? In what way was it useful.

0Was the online digital tool easy for you to access? explain why or why not (easy to find on homepage, length of tool, clear and easy to follow instructions, language, sequence, when did you use the tool Monday, Tuesday, weekend or during the week and why)

0What information or components would have helped you better -what do you suggest needs to be done to make such an online too more accessible.

Utility as a reliable information source and decision making

0When you consulted the online digital tool; did you follow the recommended advice? Prompt (why and how socioeconomic status could have influenced the process)

0What made you follow the advice and recommendations? Prompt confirmation from friends, generally, the media influence your decision-making process?

0What made you disregard the advice and recommendations? (prompt for severity of symptoms, change of condition)

0We have noticed that people that earn below 4000 tend not to follow recommendations, why do you think it`s like this, what can be done?

Utility in allaying fear and anxiety

0Describe how you felt after consulting the online digital tool with regards to feeling anxious and or confident that all was going to be well? (Did you feel reassured after visiting corontest.ch)

0If your fears were not allayed, how did you deal with your fears? prompt on what increased confidence, what allayed your anxiety)

Illness and testing Experience

0Did you test for COVID-19, what test and experience? Did you experience COVID-19 symptoms? Explain

0Speaking of self-isolation. Please explain in more detail how you experienced this what worked and what did not work e.g. the need to go shopping, not going for a run or walk protecting others vs own needs, putting others first, dilemmas, challenges

0The road to recovery has been described by many as very cumbersome and long-what was your experience? Any psychiatric or other residual effects experienced-explain.

0What personal life lessons did you learn during this pandemic you would like to share and what personal changes do you foresee in future

0What health and health system related observations did you make and what changes do you fore see in future?

0What, socio-economic changes have you observed and do you foresee in the future as a result of COVID-19

Utility in preventing cross infection

0If you consulted a GP; did you call ahead of time?

0How did your GP/ health care provider react when you told him or her you suspected that you had COVID-19?

Recommendations

0In a future pandemic, what would you do?

0Is there any additional information you wish a site like corontest provides but was missing during COVID-19? What information did you search for but did not find?

0Are there other strategies (to allay fear, anxiety) you deem effective alone or in conjunction with online digital tools when faced with epidemics such as COVID-19 to make it accessible to the older generation?

0How can online tools like corontast be adapted to facilitate your decision making processes
