# Supplementary material for: Effects and utility of an online forward triage tool during the SARS-CoV-2 pandemic: a mixed method study and patient perspectives, Switzerland
Source: BMJ Open. 2022 Jul 11;12(7):e059765. doi: 10.1136/bmjopen-2021-059765 (PMC9274020; doi:10.1136/bmjopen-2021-059765)
Supplement: Author's manuscript [file bmjopen-2021-059765.draft_revisions.pdf]

BMJ Open

BMJ Open is committed to open peer review. As part of this commitment we make the peer review history of every article we publish publicly available.

When an article is published we post the peer reviewers' comments and the authors' responses online. We also post the versions of the paper that were used during peer review. These are the versions that the peer review comments apply to.

The versions of the paper that follow are the versions that were submitted during the peer review process. They are not the versions of record or the final published versions. They should not be cited or distributed as the published version of this manuscript.

BMJ Open is an open access journal and the full, final, typeset and author-corrected version of record of the manuscript is available on our site with no access controls, subscription charges or pay-per-view fees (http://bmjopen.bmj.com).

If you have any questions on BMJ Open's open peer review process please email info.bmjopen@bmj.com

BMJ Open

# Effects and utility of an online forward triage tool during the SARS-CoV-2 pandemic: patient perspectives

| | |
|---|---|
| Journal: | *BMJ Open* |
| Manuscript ID | bmjopen-2021-059765 |
| Article Type: | Original research |
| Date Submitted by the Author: | 01-Dec-2021 |
| Complete List of Authors: | Michel, Janet; Inselspital Universitätsspital Bern, Department of Emergency Medicine<br>Mettler, Annette; University of Bern, Department of Emergency Medicine<br>Stuber, Raphael; University of Bern, Department of Emergency Medicine<br>Müller, Martin; University of Bern, Department of Emergency Medicine<br>Ricklin, Meret; University of Bern, Department of Emergency Medicine<br>Jent, Philipp; University of Bern, Department of Infectious Diseases<br>Hautz, Wolf; University of Bern, Department of Emergency Medicine; University of Oslo<br>Sauter, Thomas; University of Bern, Department of Emergency Medicine |
| Keywords: | COVID-19, Public health < INFECTIOUS DISEASES, QUALITATIVE RESEARCH, Telemedicine < BIOTECHNOLOGY & BIOINFORMATICS, EPIDEMIOLOGY |
| | |

SCHOLARONE™
Manuscripts

*I, the Submitting Author has the right to grant and does grant on behalf of all authors of the Work (as defined in the below author licence), an exclusive licence and/or a non-exclusive licence for contributions from authors who are: i) UK Crown employees; ii) where BMJ has agreed a CC-BY licence shall apply, and/or iii) in accordance with the terms applicable for US Federal Government officers or employees acting as part of their official duties; on a worldwide, perpetual, irrevocable, royalty-free basis to BMJ Publishing Group Ltd ("BMJ") its licensees and where the relevant Journal is co-owned by BMJ to the co-owners of the Journal, to publish the Work in this journal and any other BMJ products and to exploit all rights, as set out in our licence.*

*The Submitting Author accepts and understands that any supply made under these terms is made by BMJ to the Submitting Author unless you are acting as an employee on behalf of your employer or a postgraduate student of an affiliated institution which is paying any applicable article publishing charge ("APC") for Open Access articles. Where the Submitting Author wishes to make the Work available on an Open Access basis (and intends to pay the relevant APC), the terms of reuse of such Open Access shall be governed by a Creative Commons licence – details of these licences and which Creative Commons licence will apply to this Work are set out in our licence referred to above.*

*Other than as permitted in any relevant BMJ Author's Self Archiving Policies, I confirm this Work has not been accepted for publication elsewhere, is not being considered for publication elsewhere and does not duplicate material already published. I confirm all authors consent to publication of this Work and authorise the granting of this licence.*

For peer review only

# Effects and utility of an online forward triage tool during the SARS-CoV-2 pandemic: patient perspectives

Janet Michel[1]*, Annette Mettler[1]*, Raphael Stuber[1], Martin Müller[1], Meret E. Ricklin[1], Philipp Jent[3], Wolf E. Hautz[1,2], Thomas C. Sauter[1]

**Affiliations:**
[1] Department of Emergency Medicine, Inselspital, University Hospital, University of Bern, Bern, Switzerland
[2] Centre for Educational Measurement, University of Oslo, Oslo, Norway
[3] Department of Infectious Diseases, Inselspital, University Hospital, University of Bern, Bern, Switzerland
* Co-first authorship

For peer review only

**Author Details**

**Corresponding Author:**
Janet Michel RN, BA Cur, MPH, PhD
Department of Emergency Medicine
Inselspital, University Hospital, University of Bern.
3010 Bern, Switzerland
E-Mail: janetmichel71@gmail.com
Phone: +41 326322111

**Author 2**
Annette Mettler
Department of Emergency Medicine
Inselspital, University Hospital, University of Bern.
3010 Bern, Switzerland
E-Mail: annette.mettler@insel.ch

**Author 3**
Raphael Stuber
Department of Emergency Medicine
Inselspital, University Hospital, University of Bern.
3010 Bern, Switzerland
E-Mail: raphael.stuber@students.unibe.ch

**Author 4**
Martin Müller
Department of Emergency Medicine
Inselspital, University Hospital, University of Bern.
3010 Bern, Switzerland
E-Mail: martin.mueller2@insel.ch

**Author 5**
Meret E. Ricklin
Department of Emergency Medicine
Inselspital, University Hospital, University of Bern.
3010 Bern, Switzerland
E-Mail: meret.ricklin@insel.ch

**Author 6**
Philipp Jent
Department of Infectious Diseases
Inselspital, University Hospital, University of Bern.
3010 Bern, Switzerland
E-Mail: philipp.jent@insel.ch

**Author 7**
Wolf E. Hautz
Department of Emergency Medicine
Inselspital, University Hospital, University of Bern.
3010 Bern, Switzerland
E-Mail: wolf.hautz@insel.ch

**Author 8**
Thomas C. Sauter
Department of Emergency Medicine
Inselspital, University Hospital, University of Bern.
3010 Bern, Switzerland
E-Mail: thomas.sauter@insel.ch

**Word count 5 600**

**Key words**

online forward triage tool (OFTT), effects, utility, SARS-CoV-2, COVID-19, pandemic

For peer review only

# ABSTRACT

**Introduction:** Online forward triage tools (OFTT) are being widely used during this COVID-19 pandemic. The effects and utility of such tools however, have not been widely assessed.
Objective**:** To assess the effects (quantitatively) and the utility (qualitatively) of a COVID-19 OFTT in a pandemic context, exploring patient perspectives as well as eliciting recommendations for OFTT improvement.
**Methods**: We employed a mixed-method sequential explanatory study design. Quantitative data of all users of the OFTT between March 2nd, 2020 and May 12th, 2020 were collected. A follow-up survey of people who consented to participation was conducted. Secondly, qualitative data was collected through key informant interviews (n=19) to explain the quantitative findings, as well as explore tool utility, user experience and elicit recommendations.
**Results:** In the study period, 6,272 users consulted our OFTT; 40.2% (1626/4049) would have contacted a healthcare provider had the tool not existed. 560 participants consented to a follow-up survey and provided a valid e-mail address. 31.4% (176/560) participants returned a complete follow-up questionnaire. 84.7% (149/176) followed the recommendations given. 41.5% (73/176) reported that their fear was allayed after using tool. Qualitatively, seven overarching themes emerged namely i) accessibility of tool, ii) user-friendliness of tool, iii) utility of tool as an information source, iv) utility of tool in allaying fear and anxiety, v) utility of tool in medical decision making vi) utility of tool in reducing the potential for onward transmissions and vii) utility of tool in reducing health system burden.
**Conclusion:** Our findings demonstrated that a COVID-19 OFTT does not only reduce the health system burden, but can also serve as an information source, reduce anxiety and fear, reduce cross infections and facilitate medical decision making.

**Word count 277**

**Strengths and limitations**

Many online tools have been developed during the COVID-19 pandemic. The effects and utility of these tools however have not been assessed.

- *Coronatest.ch* was one of the first COVID-19 OFTTs in Switzerland. Our study could become the base line for studies that assess the effects and utility of such online tools. The identified themes namely i) accessibility of tool, ii) user-friendliness of tool, iii) utility of tool as an information source, iv) utility of tool in allaying fear and anxiety, v) utility of tool in decision making (test or not to test), and vi) utility of tool in reducing onward transmission-cross infection, vii) utility of tool in reducing health system burden, could serve as a framework for assessing OFTT utility (follow-up paper). The mixed method sequential explanatory design gave us a better understanding of OFTTs, their effects measured quantitatively and utility explained with the aid of qualitative findings. We did not simply report the effects but could also explain why the results were that way, generating a holistic picture of the phenomenon.
- The selection of the participants in our study carries the risk of a selection bias. Perspectives of those that do not use online tools are missing and should be explored in further studies. In addition, only a limited number of OFTT users took part in our study. This selection bias cannot, to the best of our knowledge, be prevented due to data

For peer review only

protection regulations which impose a voluntary participation and prohibit a technically possible automatic tracking of participants. Another way to avoid this possible selection bias would be to make the use of such a tool conditional on participation in the study. We have deliberately decided against this procedure for ethical reasons, in order to make our OFTT accessible to as many users as possible and to keep barriers as low as possible. In addition, mandatory entry of personal data in OFTT for study purposes would also discourage individuals from using the tool and thus trigger a new bias. Our comparison of overlapping questions between the OFTT and the follow-up survey can at least help to estimate the similarities within the two groups. For both questions, the percentages are comparable and can help in estimating the similarity of the groups.

- As with all online tools, we cannot confirm the accuracy of the data entered. In particular, we cannot say for sure whether the OFTT users used the tool to assess own symptoms or for other reasons, such as curiosity, fear or uncertainty about how to deal with the novel infection. Likewise, multiple use, trial runs or use of tool by a health care worker on behalf of patients, relatives and friends are all possible. Socio-economic status might have introduced a selection bias in our study since most of the participants had a higher education. Income emerged not to be a good proxy for assessing socio-economic status. Other instruments, apart from income are therefore needed to assess socio-economic status. Additionally, an on online assessment cannot fully replace a (polymerase chain reaction) PCR test as some asymptomatic people might be positive and those with COVID-19 specific symptoms might be suffering from a different disease.[5]

In our mind, the data still sheds light on the effects and utility of such an online tool and the recommendations given could guide other OFTT developers as the third wave sweeps across Europe. As the study was conducted with a specific OFTT, transferability of our results to other OFTTs is not necessarily a given. Given the limited evidence on the use of OFTTs, the results, in particular the qualitative component of the study, could be of value to other OFTT developers, with particular regards to utility and accessibility issues. Further studies with other OFTTs outside the COVID-19 context are recommended so as to increase transferability and improve the utility of OFTTs in the current third wave, future pandemics and other health care settings.

For peer review only

**What is already known**

- Online forward triage tools (OFTT) are being widely used during this COVID-19 pandemic as misinformation and worry in the population abound. However, there is a dearth of studies on the effects and utility of these OFTTs to the end-users (patient voices missing).

**What this study adds**

- Several studies have revealed that OFTTs reduces the health system burden but the voices of the end-users on how useful these OFTTs are, are scant and missing. That makes our study one of the firsts in assessing the effects and utility of a COVID-19 OFTT utilizing a mixed method approach. The mixed method sequential explanatory design gives a better understanding of OFTTs, their effects measured quantitatively and utility explained with the aid of qualitative findings. We did not simply report the effects but could also explain why the results were that way, generating a holistic picture of the phenomenon.
- Over and above the agreed notion that OFTTs reduce the health system burden, our study provided further evidence for the utility of OFTTs to end users namely: serving as an information source, allaying fear and anxiety, reducing potential for onward transmission and facilitating decision making.

**Policy implications**

- Systems thinking-refers to the ability to see interconnectedness in a system with a dysfunction in one part affecting other parts and consequently outcomes. Our study revealed the reasons patients did not follow the OFTT recommendation to test, as multipronged. Attention has to be paid to supply chain issues, as test shortages affected outcomes. The cost of a test and the fear of a positive result additionally emerged as hindrances to testing. Noteworthy, is the reaction of GPs who labelled OFTT users who asked for a COVID-19 test as hysteric. This demonstrates the need to involve, collaborate with and win the local health care providers like GPs and Spitex (home based nursing)-policy implementers, in OFTT development to ensure buy in and positive outcomes when implementing such a tool. All the above calls for systems thinking in implementation.
- Many elderly people are willing to embrace telemedicine. Telephone and voice activated system for the older population or call centers to serve this group, are still needed during this transitional phase.

For peer review only - http://bmjopen.bmj.com/site/about/guidelines.xhtml

# INTRODUCTION

The number of COVID-19 cases across the globe has surpassed 25 million and incident rates are again on the rise as many European countries experience subsequent waves.[1–4] Many people are seeking reliable information, recommendations on testing and management of COVID-19 as well as reassurance, adding to the health system burden. Online forward triage tools (OFTT) are being widely used during this COVID-19 pandemic context[5–8] as misinformation and worry in the population abound. There is evidence from an earlier 2009 H1N1 influenza pandemic, that online tools are effective and practical in reducing the health system burden.[9,10] There is also emerging evidence of this nature from the COVID-19 context. [6,11–14] For example, OFTTs help reduce exposure of worried but uninfected and infected persons, through avoidance of hospitals and doctors' offices – enabling patients to access recommendations of what to do, from the comfort of their own homes.[10,11]
Using OFTTs is relatively easy to the computer literate. People respond to questions and upon completion, recommendations are given, e.g., isolate, test, do not test etc. Existing evidence on the effects and utility of OFTTs differ with possible implications on the quality of the symptom assessment [5]. According to literature, the reasons patients use symptom checkers or OFTTs are i) to understand the causes of their symptoms (76%), ii) to determine whether or not to seek care (33%), and iii) where to seek care (21%).[15] There is also evidence that patients that have previously experienced a diagnostic error are more likely to use OFTT to search for where to seek care [15] than those that have not.

### Challenges with OFTT use and research gap

In the European Union, 87% of people aged 75 years and above have never been online according to a recent survey.[16]That means the elderly, may be less inclined to use online tools if not computer literate. This in turn shuts the elderly out from society, increasing isolation and loneliness, not to mention the missed health benefits [10]. The digital divide is real [17]. How can digital tools be designed to be more inclusive?[18] Information on factors influencing the use of OFTTs is scant and the validation of COVID-19 OFTTs like other OFTTs, seems neglected. [15,19] That makes the quality assessment of these tools paramount [5] as evidence on effects and utility of OFTTs is limited.

### The aim of this study

This study aimed at assessing the effects (quantitatively) and the utility (qualitatively) of a COVID-19 OFTT during a pandemic context in Switzerland, exploring patient perspectives and derive recommendations for tool improvement. We hypothesized that an OFTT adequately reduces patient visits to the health care system and consequently reduces the health system burden. We further explored qualitatively, for emergent themes, capturing the tool utility to this population.

For peer review only

# METHODS

## Study design and participants

We employed a mixed-method sequential explanatory design to study the utility of the OFTT and the effects of using such a tool. The rationale for mixing both kinds of data within one study is that neither qualitative nor quantitative methods, are sufficient by themselves, to capture details of a phenomenon. In combination, they complement each other, taking advantage of the strengths of each. As in sequential explanatory designs, quantitative data collection was done first, as a major component of our study to inform qualitative interviews, see Figure 1.

**About here Figure 1: Mixed-Methods Sequential Explanatory Study Design.**

## Online forward triage tool description and setting

The working group e-emergency medicine at the emergency department (ED), Inselspital University Hospital Bern, together with the Department of Infectious Diseases, Inselspital University Hospital Bern, developed an online forward triage tool (OFTT) which was made available online (*coronatest.ch*). To the best of our knowledge, this was one of the first COVID-19 OFFTs set up in the German speaking part of Switzerland. In a skip-logic, the OFTT displayed the current test recommendations of the Federal Office of Public Health (FOPH) on whether someone needed testing for COVID-19 or not. No diagnosis was provided by the OFTT.

The questions and the content of the OFTT represented the official FOPH recommendations at the time. Thus, the OFTT was comparable in content to other OFTTs in Switzerland, which were based on the FOPH guidelines within that time period. One additional non-mandatory question, which did not affect the result, was integrated in our OFTT from the 11th March 2020, namely the question "What would you do if this online test did not exist?".

There were two possible outcomes of the OFTT: "According to the criteria of the Federal Office of Public Health (BAG), one meets or does not meet the criteria for a test for an infection with the coronavirus, COVID-19". The results page was linked to the FOPH's official behavioural recommendations and recommendations for the testing process. The average time to complete the assessment was 75sec.

## Quantitative data

### Research participants and data collection

Participants included all users above the age of 18 that used the OFTT between March 2nd, 2020 and May 12th, 2020. In this timeframe, the recommendations on COVID-19 frequently changed in Switzerland and there was an initial lack of testing reagents and capacity as well as the risk of overburdening the healthcare system. During the first few weeks of the pandemic, the Federal Office of Public Health (FOPH) recommended testing only for symptomatic patients after travel to high-risk countries (e.g., Italy and China) or symptomatic contacts of coronavirus patients. In weeks that followed (as from the 20th March 2020), the strategy changed to testing of high-

For peer review only

risk groups (older than 65 years, pre-existing conditions, and healthcare workers). The countries and risk groups were regularly adjusted according to the spread of the virus and the findings about risk groups but also the availability of testing capacity.
Due to the rapid spread of the virus in Switzerland, and broadly available testing capacities, a universal test recommendation was made by the Federal Office of Public Health (FOPH)- on April 27th, 2020. All symptomatic individuals were eligible to test. With this recommendation, our OFTT provided less benefit to the user and was finally removed on May 12th 2020 from the website paving the way to a second generation OFTT.

To minimise the barrier to the use of the OFTT and for legal data protection reasons, no personal data was collected within the OFTT. Further data on the users of the OFTT was collected in a second step, from participants who gave their explicit consent and provided their email addresses to be contacted. This also made it possible to investigate the adherence to recommendations and the test results. A non-mandatory additional question was built into the OFFT from 11th March 2020.
A pretested online questionnaire (see supplementary info) was used to assess the

i) utilization of the OFTT, including way of referral to the tool, reasons for use and information searched,
ii) additional factors, including influence of the media and influence of the OFTT on fear and anxiety.

The database used is compliant with Swiss laws on the collection of personal health related information. The follow-up questionnaire is available as supplementary information. Due to ethical reasons, we included the option "not want to answer" as a choice in the questionnaire for the socio-demographic data, in case the respondent did not want to give a statement on this specific sensitive topic.
The qualitative interviews were conducted with purposefully selected key informants who gave their consent during the survey (see below).

### Data analysis

Quantitative data was analysed in Stata® 16.1 (StataCorp, The College Station, Texas, USA). Descriptive statistics for all variables as mean and standard deviation or frequency as determined by the type and distribution of the data were computed. Categorical variables between two groups were compared using Chi-square statistics and the distribution of continuous variables were compared using Wilcoxon rank sum test.
To assess the risk of selection bias and to estimate the similarity of the groups, we compared responses to overlapping questions within the OFTT and the follow-up survey.

### Qualitative data

To explain the quantitative results, we explored the experience of tool use by the patients qualitatively. Following quantitative data analysis, an interview guide was created and adapted iteratively.

### Purposeful and quota sampling

We purposefully sampled participants from those that had firstly, utilized our OFTT, secondly, had taken part in the follow-up survey and thirdly, had consented to a follow-up interview. We included participants of all age groups (quota) to ensure inclusiveness.

### Sample Size

For peer review only

Many experts suggest saturation as central to qualitative sampling [20]. In this study we aimed for both data saturation and rich and detailed narratives and achieved this with 19 key informants from all age groups (see Table 1).

### Data collection

Due to COVID-19 concerns, video rather than face to face interviews were held with most participants in September 2020. A combination of video and telephonic interviews were conducted with three participants who had technical challenges and a telephone only interview was held with one lady, aged above 65, who had no computer access. Three face to face interviews were held with three key informants: one that was a hospital health care worker, and two key informants who worked close to Bern university hospital. A semi-structured interview guide informed by the quantitative results was used (see supplementary info). This was adapted iteratively throughout the data collection period. Two qualitative researchers sat in each session fielding questions in turns. All interviews were conducted in German by two researchers fluent in both English and German. The interviews lasted between 45 minutes to one and a half hours. Two audio-recorders were used in each session. All participants gave individual written consent as well as oral consent to the recording at the beginning of each session. See Table 1 for summary of Key Informants.

**Table 1. Key Informant summary**

| Age-group | Males | Females | Total |
|---|---|---|---|
| 18-29 | 1 | 2 | **3** |
| 30-45 | 2 | 2 | **4** |
| 46-64 | 3 | 4 | **7** |
| 65+ | 4 | 1 | **5** |
| **Total** | **10** | **9** | **19** |

### Data analysis

Audio recordings were transcribed, analysed and triangulated with quantitative data results. Qualitative narratives were obtained to explain quantitative results as well as to explore utility of OFTT to patients as well as elicit recommendations to make online tools more useful and inclusive.

For peer review only - http://bmjopen.bmj.com/site/about/guidelines.xhtml

**Measures to ensure trustworthiness of data:** To ensure dependability, data collection and analysis were performed iteratively, continuously adjusting our interview guide to capture newly emerging themes. Throughout data collection, two qualitative researchers kept reflexive journals and debriefed at the end of each interview. To ensure transferability, a thick description of participants, context and data collection process has been outlined. Data was managed and analysed with the aid of MAXQDA2018.

## Ethics approval

The local ethics committee of the Canton of Bern, Switzerland, deemed this project a quality evaluation study and waived the need for full ethical review (Req-2020-00289) on the 23rd of March 2020.

## Patients and Public Involvement statement

Patients and public were not involved in the design, conduct, reporting or dissemination of this research since the OFTT was set up as an emergency response to the pandemic.

For peer review only

# RESULTS

## Quantitative results

In total, n=6,272 completed assessments of the OFTT were recorded on the website during the study period from March 2nd, 2020 to May 12th, 2020. This question asked OFTT users what they would have done had the OFTT not existed. The question was answered by 97.6% (3953/4049) of the users as follows: 40.2% (1626/4049) would have contacted the GP or visited a hospital had the tool not existed; furthermore, 16.4% (665/4049) would have contacted a hotline.

In the OFTT, 25.6% (1,608/6272) of assessments received a recommendation to test for COVID-19 during the study period. In the follow-up survey question, "Did the online tool recommend you to test for COVID-19?" -31.8% (56/176) answered, yes.

In the OFTT, 13.2% (564/4270) of OFTT users reported being over 65 years of age. The variable age was only included and mandatory during some phases of the study period in accordance with the FOPH guidelines, that changed frequently. This resulted in 4270 assessments with data on age. In the follow-up survey, 17.6% (31/176) reported being over 65 years.

A link to the online follow-up questionnaire was sent to 560 participants that consented to a follow-up survey by providing a valid e-mail address. The online questionnaire was filled out by 37.9% (212/560) of the participants; 31.4% (176/560) completed the whole questionnaire and were included in the analysis (all 22 questions-see supplement). An overview of socio-demographic characteristics of participants of the follow-up survey are presented in Table 2.

For peer review only

**Table 2. Socio-demographic table of participants of follow-up survey**

| | Total | (n=176) | Female | (n=101) | Male | (n=75) | P-value* |
|---|---|---|---|---|---|---|---|
| **Age [mean, SD]** | 50.1 | [±15.4] | 45.9 | [±14.1] | 55.7 | [±15.4] | <0.001 |
| **Education** | | | | | | | |
| Not want to answer | 6 | [3.4] | 3 | [3.0] | 3 | [4.0] | |
| University | 120 | [68.2] | 67 | [66.3] | 53 | [70.7] | |
| Higher secondary school | 27 | [15.3] | 17 | [16.8] | 10 | [13.3] | |
| Lower secondary school | 23 | [13.1] | 14 | [13.9] | 9 | [12.0] | 0.871 |
| **Income per month** | | | | | | | |
| Not want to answer | 29 | [16.5] | 17 | [16.8] | 12 | [16.0] | |
| <4000 CHF | 26 | [14.8] | 20 | [19.8] | 6 | [8.0] | |
| 4000 - 6000 | 42 | [23.9] | 27 | [26.7] | 15 | [20.0] | |
| >6000 | 79 | [44.9] | 37 | [36.6] | 42 | [56.0] | 0.037 |
| **Work** | | | | | | | |
| Not want to answer | 33 | [18.8] | 14 | [13.9] | 19 | [25.3] | |
| Employed | 106 | [60.2] | 64 | [63.4] | 42 | [56.0] | |
| Self-employed | 24 | [13.6] | 13 | [12.9] | 11 | [14.7] | |
| Unemployed | 3 | [1.7] | 3 | [3.0] | 0 | [0.0] | |
| Lost work (Covid-19) | 1 | [0.6] | 1 | [1.0] | 0 | [0.0] | |
| Student/trainee | 9 | [5.1] | 6 | [5.9] | 3 | [4.0] | 0.236 |
| **Insurance** | | | | | | | |
| Don't know | 5 | [2.8] | 3 | [3.0] | 2 | [2.7] | |
| General | 68 | [38.6] | 39 | [38.6] | 29 | [38.7] | |
| Telemedicine | 12 | [6.8] | 6 | [5.9] | 6 | [8.0] | |
| GP | 83 | [47.2] | 47 | [46.5] | 36 | [48.0] | |
| Other | 8 | [4.5] | 6 | [5.9] | 2 | [2.7] | 0.859 |
| **Nationality** | | | | | | | |
| Not want to answer | 1 | [0.6] | 1 | [1.0] | 0 | [0.0] | |
| Switzerland | 147 | [83.5] | 80 | [79.2] | 67 | [89.3] | |
| Germany | 13 | [7.4] | 8 | [7.9] | 5 | [6.7] | |
| French | 1 | [0.6] | 0 | [0.0] | 1 | [1.3] | |
| Italy | 3 | [1.7] | 2 | [2.0] | 1 | [1.3] | |
| Other Europe | 4 | [2.3] | 3 | [3.0] | 1 | [1.3] | |
| Other | 7 | [4.0] | 7 | [6.9] | 0 | [0.0] | 0.202 |

For peer review only

* Chi-squared for categorical variables and Wilcoxon rank sum test for continuous variables; data are total number and percentage if not mentioned otherwise

The survey revealed that 84.7% (149/176) followed the tool recommendations and stayed at home thereby reducing the work-load of GPs and hospitals. Information about the utilization of the OFTT, specifically which information was searched for, how subjects found the tool, and information about satisfaction with the tool is presented in Table 3.

**Table 3. Online forward triage tool use**

| | Total (n=176) | [%] |
|---|---|---|
| Information searched | | |
| Information on COVID-19 symptom | 97 | [55.1] |
| How to cope with symptoms | 4 | [2.3] |
| To know when to consult a doctor | 36 | [20.5] |
| To know more on testing criteria | 32 | [18.2] |
| To know where to test | 7 | [4.0] |
| Mode of referral | | |
| Referral by family doctor | 9 | [5.1] |
| Online search | 113 | [64.2] |
| Recommendation by peers | 17 | [9.7] |
| Hotline | 2 | [1.1] |
| Other | 35 | [19.9] |
| Satisfaction with information | | |
| Helpful | 154 | [87.5] |
| Not comprehensive | 17 | [9.7] |
| Not clear | 5 | [2.8] |

We present additional factors that may have influenced how individuals coped during the coronavirus pandemic, their use of the OFTT and adherence to OFTT recommendations. Overarching topics that were asked included the influence of the media, fear and uncertainty, and reasons for adherence to the recommendation (see table 4). All questions and answers from the follow-up questionnaire are attached. See supplement 1.

For peer review only

**Table 4. Additional factors**

| | Total (n=176) | [%] |
|---|---|---|
| Estimated influence of media | | |
| Helpful | 81 | [46.0] |
| Confusing | 47 | [26.7] |
| No trust in media as source of information | 25 | [14.2] |
| Other | 23 | [13.1] |
| Influence of OFTT on fear and anxieties | | |
| Reassured | 73 | [41.5] |
| No reassurance | 13 | [7.4] |
| Increased fears and anxieties. | 6 | [3.4] |
| Not worried before OFTT use | 84 | [47.7] |
| Reasons for following the recommendation (n=149) | | |
| Trust in tool | 60 | [40.3] |
| Information congruent with media | 20 | [13.4] |
| Comparison with FOPH recommendation | 53 | [35.6] |
| Reassurance by others | 7 | [4.7] |
| Other | 9 | [6.0] |

## Qualitative findings

Seven overarching themes on the utility of the OFTT emerged during the qualitative interviews. These are used to structure the report of our findings, i.e., i) accessibility of the tool, ii) user-friendliness of the tool, iii) utility of the tool as an information source, iv) utility of the tool in allaying fear and anxiety, v) utility of the tool in decision making (test or not to test), vi) utility of the tool in reducing onward transmission-cross infection, and vii) utility of the tool in reducing health system burden. The qualitative findings are summarised in Table 5.

For peer review only

**Table 5. Summary of qualitative themes**

| Theme | Category | Unit meaning |
|---|---|---|
| **Accessibility** | Online search | Appeared but not on the top of google search<br>Advertise tool in future |
| | Unreachable for some | Include telephonic services to reach the elderly<br>Tool buddies |
| **Utility as a reliable information source** | COVID-19 Symptoms | Cough was a main symptom<br>Symptom description like type of cough and severity of fever etc. was not possible<br>Test or do not test decision was arbitrary-how the decision was arrived at was not clear e.g., 95% probability test or 5% probability do not test |
| | Testing info and centres missing | Information on when to call doctor was not clear e.g., fever above 39 degrees for 4 days -call doctor<br>List of where to test and contact numbers were missing |
| **Utility in decision making** | Followed recommendations | Trust- the university hospital is a trusted institution |
| | Did not follow recommendations | Fear of a positive result and the resultant consequences<br>Cost of test<br>Test shortage<br>GP refusing patients to test -hysteria |
| **Utility in allaying fear and anxiety** | Reassured some | Fear and anxiety allayed after tool use |
| | Person contact | An online tool is still an online tool -recommendations seen as not having a lot of weight<br>A talk with a general practitioner (GP)-debriefing after tool use could have put them at ease |
| | Testing | Testing in itself is reassuring -make test available to all who are anxious |
| | Friends and family as a resource | Many relied on family and friends to deal with fear- social circle still a major source of support |
| | Increased anxiety in some | |

| | | |
|---|---|---|
| | | High risk label unsettled some |
| **Utility in reducing health system burden** | Many stayed at home | Recommendations followed- stay at home<br>Some called Insurance companies |
| **Utility in reducing onward transmission** | Call GP before a visit | Most called GP ahead of visit |
| **Systems thinking** | Utility of tool is dependent upon other health system and societal components<br><br>Fear of a positive test<br>-rather not know | Participants told by tool to test only to be told that there are no tests (shortages)<br>Fear of a positive test<br><br>Media misinformation of painful test influenced some not to test-work with media<br><br>Economic factors like cost of test influenced some not to test<br>A new life-threatening disease in a population is associated with psycho-social and behavioural issues that need to be taken into account |

**Theme 1: Accessibility of the tool**

The accessibility of the tool emerged as very important. Many participants suggested to advertise the tool to make it more accessible as revealed below:

*"I did not know of the existence of tool (an accidental internet search led the key informant to the tool). Please advertise tool on TV and to Insurance companies."* -Key Informant 15

The older people seem willing to embrace technology and were prepared to use it. However, they stated that they needed help with practical application at times as revealed below;

*"Provide telephone services for the elderly and a contact person, a GP so one can ask questions if unsure."* -Key Informant 14

**Theme 2: User-friendliness of the tool**

Most participants could not remember the tool immediately due to the time lapse from the tool usage to interview. After being shown the tool once again, the header only, many cited it as having been easy and simple to follow with the language being clear and the length acceptable.

**Theme 3: Utility of the tool as an information source**

The novel nature of COVID-19 infection left many scrambling for knowledge of the disease. Many health care providers were inundated with phone calls. One participant said the following;

*"The tool provided information on symptoms but did not have a list of testing centers. The recommendations said call GP before visit but there was no number to call."* -Key Informant 1

*"Telemedicine could play a better information spreading role – media spread fear and misinformed people for example mask use vs no mask."* -Key Informant 15

**Theme 4: Utility of the tool in allaying fear and anxiety**

Many participants interviewed reported being reassured after tool use. Others cited being more anxious after tool use due to terminology and language and many suggested that a person, a doctor be available after tool use for closure. Participants revealed the following;

*"Wording of tool could be adapted – a friend aged 65, a diabetic, became depressed after using tool and getting the high-risk patient classification. He needed a psychiatrist to cope. Rather ask how are you, do you take any medication, which ones? Mentioning conditions seem to increase anxiety."* -Key Informant 17

*"I felt discriminated against by tool-differentiate between a health 73-year-old with no chronic illnesses and a 50-year overweight diabetic."* -Key Informant 13

**Theme 5: Utility of the tool in decision making process (to test or not to test)**

Many participants cited trust in our university hospital (Insel) as one of the main reason participants followed the recommendations. Some participants revealed the following;

*"Insel has a good name and trusted the tool."* -Key Informant 16

*"Coordination is needed for FOPH and Insel to speak in one voice."* -Key Informant 17

Juxtaposed and not necessarily contradicting the quantitative survey, where trust was reported as the main reason for following the recommendations, most of the participants cited shortages of tests, improved symptoms, cost of test, misinformation that the test was painful and fear of a positive result as reasons for not testing. Of utmost importance were GPs who viewed the test request by online tool users as being hysteric. Below is what some participants said:

*"I read scientific papers to inform oneself and then decided."* -Key Informant 8

*"Remember recommendations from an online tool have less weight than recommendations from a doctor – there is no person behind this and so many might have taken the tool and went further to contact own GP"*- Key Informant 8

*"I wished to see an algorithm that said something like, "the probability of you having COVID-19 is 75% test or 25% do not test."*-Key Informant 5

**Theme 6: Utility in reducing the potential for onward transmission- cross infection**

The tool recommended all participants to call the health care provider ahead of visit and most of them did. A reason some participants might not have called the testing centres ahead of a visit could be that the tool itself did not provide a list of contact numbers-a short coming that was rectified in the second generation OFTT.

For peer review only

**Theme 7: Utility of tool in reducing health system burden**

Social distancing, isolation and quarantine were among the recommendations made to reduce the spread of COVID-19. Most of the participants stayed at home. One participant said the following;

*"I followed recommendations and stayed at home. However, home testing should be provided if people should stay at home. Engage Spitex [organization for outpatient and home-based care in Switzerland] in future pandemics and work with them."* -Key Informant 6

For peer review only

# DISCUSSION

This study quantitatively assessed the effects and confirmed the utility (qualitatively) of a COVID-19 online forward triage tool by exploring patient perspectives. We further elaborate on areas for improvement as well as share lessons learned for policy makers. Qualitatively, seven overarching themes emerged namely i) accessibility of tool, ii) user-friendliness of tool, iii) utility of tool as an information source, iv) utility of tool in allaying fear and anxiety, v) utility of tool in decision making (test or not to test), vi) utility of tool in reducing the potential for onward transmissions (preventing cross infection) and vii) utility of tool in reducing health system burden.

**Accessibility of OFTT**

One of the objectives of our OFTT was to provide an easily accessible, reliable and up to date information platform for professionals and the public. The tool was not advertised commercially; hence it did not appear at the top of the google search and many participants cited coming across the tool accidentally. Information about the tool was only disseminated via the hospital website and hospital communication to local doctors.

Despite the above -mentioned shortcoming, our findings revealed that the tool was accessible to both genders and all age groups including the elderly. In line with other studies,[21] the elderly seem ready to embrace online tools, contradicting other studies.[10,17] Contradicting our findings, one study revealed that it's the young and highly educated patients that tend to use symptom checkers or OFTTs.[22]

Despite the revealed readiness of the elderly to embrace technology, key informants suggested keeping the use of telephonic services for the elderly as an option in telemedicine. Further supporting these findings, nurse triage lines (telephone) have been proven effective in this COVID-19 pandemic context in the US and in Canton Vaud, Switzerland.[10,23] Others suggested having a list of tool buddies reachable by phone, that links people that have used the tool before and are willing to be contacted by a new user, that might be experiencing challenges in using the OFTT. With regards to reaching the low education and low-income group, additional studies need to be done as those who earned less than CHF 4000 were not necessarily lowly educated but PhD and post doc students, concurring with findings elsewhere.[24]

**User-friendliness of OFTT**

Most of the participants could not recall tool, but after showing them tool header only, many cited tools as user-friendly, easy, with a clear language and an acceptable length, concurring with a study that was conducted elsewhere.[25] In support of our findings, online tools have been shown to be risk averse as compared to health care professionals and the users have expressed high levels of satisfaction.[22] The optimal amount of time spent filling in OFTT questionnaires nor the optimal number of questions an OFTT should ask in general, is still unclear[26] and warrants further studies.

**Utility of OFTT as an information source**

Overall, the tool was very useful in providing information on signs and symptoms. Information on where to test (list with contact numbers), how to self-care, when to contact a GP were cited by some as shortcomings and ought to be included to make the tool comprehensive in future. Information challenges with OFTTs have also been reported elsewhere.[27,28] This finding underlines the need to have an option to talk directly to a GP after OFTT use so as to debrief.

Further information or links to comprehensive and reliable sources with information on how to self-care and when to contact a GP or health care centre emerged as gaps that need to be incorporated in COVID-19 OFTTs so as to increase their utility as information sources.

For peer review only

The majority of our participants were highly educated, and this segment of the population seems to inform itself, by consulting a variety of scientific sources as well as keeping abreast with the FOPH announcements. In the context of a novel infection, where guidelines change quickly and continuously, the credibility of the tool to the highly educated, could be enhanced by stipulating sources of information and referencing and dating the FOPH criteria informing the tool.

**Utility of OFTT in allaying fear and anxiety**

For most of the participants, the tool was effective in allaying their fear and anxiety. Many wished a human presence, a doctor to debrief with after the online tool use as mentioned above. There was however, a downside for some that felt labelled as being high risk. For this group, the tool had a negative effect and increased their anxiety. Other studies have revealed similar effects.[29,30] This raises the issue of language and terminology use in such tools. Bearing in mind that COVID-19 is a novel condition, not well understood and considered fatal, the impact of a high-risk label should not be underestimated, including discrimination. Concurring with our findings, COVID-19 stigma has been reported elsewhere [31]. Many participants reported fear of a positive test result and the consequences thereof, concurring with findings from elsewhere. [32,33] Further concurring with our findings, lasting psychological consequences that last beyond the COVID-19 infection itself have also been revealed.[31] This raises the question of psychological readiness to deal with such a diagnosis. Emerging studies have reported COVID-19 patients as having psychiatric related conditions post infection, further concurring with our study.[34,35]

**Utility of OFTT in facilitating decision making**

The tool was useful in assisting patients in decision making particularly not to test. Trust in the institution proved pivotal as many followed recommendations simply because they trusted the source of the tool, our university hospital. Studies elsewhere concur with our findings.[36,37] On the other hand, some of those that got the recommendation to test did not do so due to a myriad of reasons as revealed above. In addition, the cost of the test (CHF 180 at the time), shortages of tests and fear of a positive result and the resultant consequences of isolating, stigma etc. further influenced decisions not to test. A low income was found not to be a reliable socio-economic status proxy in our study. Most low-income participants were PhD students and post-docs who cited various reasons for not following recommendations. Many told us how they sought and read scientific evidence to inform themselves and this, rather than the recommendations, guided their decision making. In line with our findings, salary is not a good proxy for socio-economic status among online tool users.[24] A shortcoming in this regard, was the missing information on how the tool arrived at the recommendation to test or not to test e.g. algorithm used[19] something some key informants wished to know. The issue of safety concerns with regards to specificity of digital tool algorithms has also been reported elsewhere.[38]

**Utility of OFTT in preventing onward transmission- cross infection**

The tool proved useful in preventing cross infection concurring with findings elsewhere.[19] Most participants who were told to stay at home did so, reducing mobility and exposure. Most of the participants called the GP practice ahead of time. That gave the GP practices time to ensure that the suspect patient did not mix with other patients, thereby reducing the potential for onward transmission (cross infection).[19]

**Utility of OFTT in reducing health system burden**

For peer review only

Our primary hypothesis was that such an OFTT reduces the health system burden. Most of the participants who used the tool would have called their GP or visited the hospital. OFTT use effectively kept these worried participants at home and out of the doctors' offices and hospitals, effectively reducing the health system burden. Contradicting our findings, research from elsewhere has produced inconclusive and sometimes contradicting evidence.[28,39] Further studies in different contexts are therefore called for. Further contradicting our findings,, another study reported that symptom checkers` triage capabilities are not greater than that of an average lay person.[40] In fact the convenience of telemedicine has also been associated with increased utilization of services, increasing work load and health care spending.[41]

**Recommendations and lessons learned**

Our study demonstrated the effects and utility of a COVID-19 OFTT. The assessment of an OFFTT is important but not without challenges. Below are some of the lessons worth sharing with both health care providers and policy makers as subsequent waves sweep across Europe;

- Most of the participants had challenges remembering the tool. Immediate feedback e.g., in one minute, please rate this tool, or three open questions; please tell us how useful this tool was with regards to i) accessibility of tool, ii) utility of tool as an Information source, ii) utility of tool in facilitating your decision making could be more effective. Data protection concerns and the need to keep barriers to use as low as possible, could stand in the way of this approach.
- The tool simply instructed patients to test or not to test, an arbitrary decision, without shedding light on how the decision was made. Patients wish to see an algorithm that says something like, "*the probability of you having COVID-19 is 75% test or 25% do not test.*"
- Many participants said, "*bear in mind that online tool recommendations have less weight than recommendations from a GP.*" Additional caution is needed in language and terminology use as some patients that felt labelled by tool as high risk, had negative outcomes. Ensuring access to a doctor to debrief with after such tool use is advisable. Retired doctors who are still willing to make a contribution to the society, could play such a role.
- Many participants found the tool by accident; hence it is advisable to advertise tool on social media platforms, billboards, TV, radio and could make it appear at the top of google search. In addition, taking the tool to the people e.g., through road shows could be a useful strategy to reach the old people – if they do not come to the tool, take the tool to the people.
- Many participants compared the tool recommendations with what the Federal Office of Public Health (FOPH) recommended at the time. Having a tool link on FOPH website that stipulates and references the FOPH criteria informing the tool, could increase trust in tool and acceptability. Coordination between FOPH, university hospitals, and other medical professional bodies is recommended to further enhance trust in the tool.
- Many elderly people are willing to embrace telemedicine, but challenges persist. Telephone and voice activated system for the older population or call centers to serve this group, are still needed (taking heed of unreachable and unanswered calls) during this transitional phase.
- Most participants found media confusing – telemedicine could play a better information spreading role, sifting through the noise and offering scientific based recommendations. For many, the media spread fear and misinformed people in many instances.
- The OFTT lacked information on where to test (contact list of testing centres), how to self-care, how to manage symptoms and when to contact a doctor-

For peer review only

addressing these shortcomings could improve the utility of OFTTs. Our results underline the importance of not offering a telehealth tool as a stand-alone product, but to integrate it into an overall concept with links to credible reliable sources.

- Systems thinking-refers to the ability to see interconnectedness in a system with a dysfunction in one part affecting other parts and consequently outcomes. Our study revealed the reasons patients did not follow the recommendation to test, as multipronged. Attention has to be paid to supply chain issues, as test shortages affected outcomes. The cost of a test and the fear of a positive result additionally emerged as hindrances to testing. This calls for systems thinking. Noteworthy, is the reaction of GPs who labelled OFTT users who asked for a COVID-19 test as hysteric. This does not only reveal that the pandemic caught everyone by surprise, but also demonstrates the need to involve, collaborate with and win the local health care providers-policy implementers, like GPs and Spitex (home based nursing), to enhance tool utility as well as ensure positive outcomes
- One key informant suggested having patients who had recovered from COVID-19 act as champions to share their illness experience, and motivate the public to take preventive measures and take the disease seriously-an approach that was also effective in HIV prevention and coping strategies.

For peer review only

# Conclusion

OFTT use has increased greatly during this pandemic. The effects and utility of such tools however, have not been widely assessed. That makes our study, one of the firsts, in assessing effects and utility of a COVID-19 OFTT. Our study revealed that an OFTT does not only reduce the health system burden but can also serve as an information source, reduce anxiety and fear, reduces potential for onward transmission and facilitate decision making.

For peer review only

# DECLARATIONS

**Acknowledgement:** Emergency telemedicine at University of Bern, Switzerland is supported by an endowed professorship by the Touring Club Switzerland.

**Author contributions:** Study design and idea: J.M., A.M, R.S, T.C.S., M.M, M.E.R, P.J, W.E.H.; Data extraction and preparation: R.S., M.M, A.M; Qualitative interviews: J.M., R.S., A.M.; Statistical analysis: M.M.; Qualitative analysis: JM. Writing of first draft: J.M., A.M, M.M.; Revision of the final draft and final approval: all authors; Supervision: T.C.S., W.E.H.; Project administration: T.C.S., W.E.H.

**Category of article:** original article

**Running title:** Usage of online tools during the COVID-19 pandemic

**Source of funding:** Swiss National Science Foundation (Project ID: 196615)

**Conflict of interest:** This manuscript is partially founded by the Swiss National Science Foundation (Project ID: 196615). The founder has no influence on the content of the manuscript or the decision to publish it.
TCS holds the endowed professorship for emergency telemedicine at University of Bern, Switzerland. The founder has no influence on the research performed, the content of any manuscript or any decision to publish. All other authors have nothing to disclose.

**Data sharing**
Due to the nature of the study (OFTT) participants did not agree for their data to be shared publicly. The data to support findings are available. Please contact corresponding author JM.

For peer review only

# REFERENCES


1. Higgins-Dunn N. Coronavirus cases surpass 25 million as pandemic strains nations around the world. CNBC. Published August 30, 2020. Accessed August 31, 2020. https://www.cnbc.com/2020/08/30/global-coronavirus-cases-surpass-25-million-as-pandemic-strains-nations-around-the-world.html

2. Switzerland Coronavirus: 42,393 Cases and 2,010 Deaths - Worldometer. Accessed September 1, 2020. https://www.worldometers.info/coronavirus/country/switzerland/

3. Germany Coronavirus: 244,792 Cases and 9,371 Deaths - Worldometer. Accessed September 1, 2020. https://www.worldometers.info/coronavirus/country/germany/

4. United Kingdom Coronavirus: 335,873 Cases and 41,501 Deaths - Worldometer. Accessed September 1, 2020. https://www.worldometers.info/coronavirus/country/uk/

5. Munsch N, Martin A, Gruarin S, et al. *A Benchmark of Online COVID-19 Symptom Checkers*. Health Informatics; 2020. doi:10.1101/2020.05.22.20109777

6. Hollander JE, Carr BG. Virtually Perfect? Telemedicine for Covid-19. *N Engl J Med*. 2020;382(18):1679-1681. doi:10.1056/NEJMp2003539

7. Schrager J, Schuler K, Wright D, et al. Development and Usability Testing of a Web-based COVID-19 Self-triage Platform. *West J Emerg Med*. 2020;21(5). doi:10.5811/westjem.2020.7.48217

8. Hamm JM, Greene C, Sweeney M, et al. Telemedicine in the emergency department in the era of COVID-19: front-line experiences from 2 institutions. *J Am Coll Emerg Physicians Open*. n/a(n/a). doi:10.1002/emp2.12204

9. Kellermann AL, Isakov AP, Parker R, Handrigan MT, Foldy S. Web-Based Self-Triage of Influenza-Like Illness During the 2009 H1N1 Influenza Pandemic. *Ann Emerg Med*. 2010;56(3):288-294.e6. doi:10.1016/j.annemergmed.2010.04.005

10. Spaulding AB, Radi D, Macleod H, et al. Design and Implementation of a Statewide Influenza Nurse Triage Line in Response to Pandemic H1N1 Influenza. *Public Health Rep*. 2012;127(5):532-540.

11. Gann B. Giving patients choice and control: health informatics on the patient journey. *Yearb Med Inform*. 2012;7:70-73.

12. Joshi AU,, Lewiss RE. Telehealth in the time of COVID-19. *Emerg Med J*. Published online 2020. doi:10.1136/ emermed-2020-209846

13. Judson TJ, Odisho AY, Neinstein AB, et al. Rapid design and implementation of an integrated patient self-triage and self-scheduling tool for COVID-19. *J Am Med Inform Assoc JAMIA*. 2020;27(6):860-866. doi:10.1093/jamia/ocaa051

14. Amir-Behghadami M, Janati A. Iranian national COVID-19 electronic screening system: experience to share. *Emerg Med J*. 2020;37(7):412-413. doi:10.1136/emermed-2020-209806


For peer review only

15. Meyer AND, Giardina TD, Spitzmueller C, Shahid U, Scott TMT, Singh H. Patient Perspectives on the Usefulness of an Artificial Intelligence-Assisted Symptom Checker: Cross-Sectional Survey Study. *J Med Internet Res*. 2020;22(1):e14679. doi:10.2196/14679

16. Why do many seniors have trouble using technology? No Isolation. Accessed September 8, 2020. https://www.noisolation.com/global/research/why-do-many-seniors-have-trouble-using-technology/

17. Conger K, Griffith E. As Life Moves Online, an Older Generation Faces a Digital Divide. *The New York Times*. https://www.nytimes.com/2020/03/27/technology/virus-older-generation-digital-divide.html. Published March 27, 2020. Accessed September 8, 2020.

18. Digital inclusion for low-skilled and low-literate people: a landscape review - UNESCO Digital Library. Accessed September 8, 2020. https://unesdoc.unesco.org/ark:/48223/pf0000261791

19. Millenson ML, Baldwin JL, Zipperer L, Singh H. Beyond Dr. Google: the evidence on consumer-facing digital tools for diagnosis. *Diagn Berl Ger*. 2018;5(3):95-105. doi:10.1515/dx-2018-0009

20. Morse JM. Determining Sample Size. *Qual Health Res*. 2000;10(1):3-5. doi:10.1177/104973200129118183

21. Chambers D, Cantrell AJ, Johnson M, et al. Digital and online symptom checkers and health assessment/triage services for urgent health problems: systematic review. *BMJ Open*. 2019;9(8). doi:10.1136/bmjopen-2018-027743

22. Chambers D, Cantrell AJ, Johnson M, et al. Digital and online symptom checkers and health assessment/triage services for urgent health problems: systematic review. *BMJ Open*. 2019;9(8). doi:10.1136/bmjopen-2018-027743

23. CoronaCheck for healthcare professionals | CoronaCheck Unisanté. Accessed December 1, 2020. https://coronavirus.unisante.ch/en/professionnels

24. The not-so-straightforward measurement of socioeconomic status. The Dunn Lab. Accessed September 18, 2020. http://www.thedunnlab.com/updates/measuringses

25. Nelson CA, Pérez-Chada LM, Creadore A, et al. Patient Perspectives on the Use of Artificial Intelligence for Skin Cancer Screening: A Qualitative Study. *JAMA Dermatol*. 2020;156(5):501-512. doi:10.1001/jamadermatol.2019.5014

26. What questions are priority areas for primary care during the COVID-19 pandemic? A rapid question generation and prioritisation exercise. CEBM. Accessed July 14, 2020. https://www.cebm.net/covid-19/what-questions-are-priority-areas-for-primary-care-during-the-covid-19-pandemic-a-rapid-question-generation-and-prioritisation-exercise/

27. Advantages and Disadvantages of using Symptoms Checker ⋆ The Stuff of Success. Accessed October 19, 2020. https://thestuffofsuccess.com/2018/04/08/advantages-and-disadvantages-of-using-symptoms-checker/

28. Symptom Checkers and Self-Triage Tools in the Era of COVID-19: What's the Evidence for their Use? NODE.Health. Published April 17, 2020. Accessed September 18, 2020.

For peer review only

https://nodehealth.org/2020/04/17/symptom-checkers-and-self-triage-tools-in-the-era-of-covid-19-whats-the-evidence-for-their-use/

29. How Online Searches Fuel Health-Related Anxiety. ICANotes. Published May 7, 2019. Accessed October 19, 2020. https://www.icanotes.com/2019/05/07/how-online-searches-fuel-health-related-anxiety/

30. Van Riel N, Auwerx K, Debbaut P, Van Hees S, Schoenmakers B. The effect of Dr Google on doctor–patient encounters in primary care: a quantitative, observational, cross-sectional study. *BJGP Open*. 1(2). doi:10.3399/bjgpopen17X100833

31. The "Untold" Side of COVID-19: Social Stigma and Its Consequences in India - Prama Bhattacharya, Debanjan Banerjee, TS Sathyanarayana Rao, 2020. Accessed September 18, 2020. https://journals.sagepub.com/doi/full/10.1177/0253717620935578

32. How Fear Inhibits HIV Testing and Treatment in African Men. Contagion Live. Accessed October 19, 2020. https://www.contagionlive.com/view/how-fear-inhibits-hiv-testing-and-treatment-in-african-men

33. Desmon S. Study: Fear Keeps Men from Getting Tested for HIV. Johns Hopkins Center for Communication Programs. Published October 28, 2019. Accessed October 19, 2020. https://ccp.jhu.edu/2019/10/28/study-fear-keeps-men-from-hiv-testing/

34. More Than Half of COVID-19 Survivors in Study Reported Psychiatric Disorders. EcoWatch. Published August 4, 2020. Accessed September 18, 2020. https://www.ecowatch.com/covid-19-psychiatric-disorders-2646878306.html

35. Varatharaj A, Thomas N, Ellul MA, et al. Neurological and neuropsychiatric complications of COVID-19 in 153 patients: a UK-wide surveillance study. *Lancet Psychiatry*. 2020;7(10):875-882. doi:10.1016/S2215-0366(20)30287-X

36. Birkhäuer J, Gaab J, Kossowsky J, et al. Trust in the health care professional and health outcome: A meta-analysis. *PLoS ONE*. 2017;12(2). doi:10.1371/journal.pone.0170988

37. Brennan N, Barnes R, Calnan M, Corrigan O, Dieppe P, Entwistle V. Trust in the health-care provider–patient relationship: a systematic mapping review of the evidence base. *Int J Qual Health Care*. 2013;25(6):682-688. doi:10.1093/intqhc/mzt063

38. Anhang Price R, Fagbuyi D, Harris R, et al. Feasibility of Web-Based Self-Triage by Parents of Children With Influenza-Like Illness: A Cautionary Tale. *JAMA Pediatr*. 2013;167(2):112. doi:10.1001/jamapediatrics.2013.1573

39. EBSCO HEALTH. Enabling Patient Engagement with a Symptom Checker. Published online 2020:16.

40. Schmieding ML, Mörgeli R, Schmieding MAL, Feufel MA, Balzer F. Benchmarking Triage Capability of Symptom Checkers Against That of Medical Laypersons: Survey Study. *J Med Internet Res*. 2021;23(3):e24475. doi:10.2196/24475

41. Ashwood JS, Mehrotra A, Cowling D, Uscher-Pines L. Direct-To-Consumer Telehealth May Increase Access To Care But Does Not Decrease Spending. *Health Aff (Millwood)*. 2017;36(3):485-491. doi:10.1377/hlthaff.2016.1130

For peer review only

# LIST OF FIGURES

**Figure 1: Mixed-Methods Sequential Explanatory Study Design**

For peer review only

**Figure 1: Mixed-Methods Sequential Explanatory Study Design**

| Phase | Procedure | Product |
|---|---|---|
| **Collection of clinical data** | OFTT | Data about usage of the tool<br>Contact data for later study phases |
| **Quantitative data collection** | Questionnaires | Numeric data |
| **Quantitative data analysis** | Statistical analysis | Descriptive statistics |
| **Connecting quantitative and qualitative data** | Purposefully selecting patients for interviews | Interview guide |
| **Qualitative data collection** | Individual in-depth interviews | Interview transcripts |
| **Qualitative data analysis** | Coding and thematic analysis | Codes and themes |
| **Integration of qualitative and quantitative results** | Interpretation and explanation of quantitative results with the aid of and qualitative findings | Implications for OFTT development |

For peer review only

# Supporting information OFTT Questionnaire and results

## Supporting information 1. Usage of online tools during the COVID-19 pandemic: email survey

| | | | |
|---|---|---|---|
| v1 | **How did you get to this Online - Tool?** | | |
| | 1 - My family doctor advised me to use this tool. | 9 | 5.1% |
| | 2 - I found the information on the Internet. | 113 | 64.2% |
| | 3 - The website was recommended to me by family/friends. | 17 | 9.7% |
| | 4 - Via a telephone hotline. | 1 | 0.6% |
| | 5 - Other (Free text) | 35 | 19.9% |
| v2 | **Did you find the information that you needed?** | | |
| | 1 - Yes | 154 | 87.5% |
| | 2 - No, because the information was not comprehensive. | 17 | 9.7% |
| | 3 - No, because the information was not clear. | 5 | 2.8% |
| v3 | **What information did you search for? I wanted....** | | |
| | 1 - ... more information on COVID-19 symptoms | 97 | 55.1% |
| | 2 - ... more information on how to cope with symptoms | 4 | 2.3% |
| | 3 - ... to know when to consult a doctor | 36 | 20.5% |
| | 4 - ... to know more on testing criteria | 32 | 18.2% |
| | 5 - ... to know where to test | 7 | 4.0% |
| | 6 - Other | - | - |
| v4 | **Did the online tool recommend you to test for COVID-19?** | | |
| | 1 - Yes | 56 | 31.8% |
| | 2 - No | 120 | 68.2% |
| v5 | **Did you stick to the recommendations?** | | |
| | 1 - Yes | 149 | 84.7% |
| | 2 - No | 27 | 15.3% |
| v6 | **If you followed the Online - Tool recommendations, what made you do so?** | | |
| | 1 - I trust the website as a reliable information source. | 60 | 34.1% |
| | 2 - I compared the recommendations with recommendations from the media and took a decision. | 20 | 11.4% |
| | 3 - I compared the recommendations with those from FOPH (BAG) and took a decision. | 53 | 30.1% |
| | 4 - I sought advice from a person I trusted. | 7 | 4.0% |
| | 5 - Other, please specify: Free text | 9 | 5.1% |
| v7 | **In case you did not follow the recommendations, why did you not** | | |
| | 1 - I did not trust the website as a reliable source of information. | 1 | 0.6% |
| | 2 - The recommendations from the website differed from the media recommendations. | 2 | 1.1% |
| | 3 - I feared for my life and needed to consult a GP in person. | 6 | 3.4% |
| | 4 - Other, please specify: Free text | 18 | 10.2% |
| v8 | **Were your fears and anxieties allayed after visiting the website?** | | |
| | 1 - Yes, the information from the website reassured me. | 73 | 41.5% |
| | 2 - No, the information from the website did not reassure me. | 13 | 7.4% |

For peer review only

| | | | |
|---|---|---|---|
| | 3 - No, the information from the website increased my fears and anxieties. | 6 | 3.4% |
| | 4 - I was not worried. | 84 | 47.7% |
| v9 | **How did you cope with your fears? What helped you cope?** | | |
| | 1 - Free text | - | - |
| v10 | **In case you went to the GP, did you call ahead of time to notify them of your visit?** | | |
| | 1 - Yes | 115 | 65.3% |
| | 2 - No | 61 | 34.7% |
| v11 | **Did you get tested for Coronavirus (SARS-CoV-2 Swab)?** | | |
| | 1 - Yes | 48 | 27.3% |
| | 2 - No | 128 | 72.7% |
| v12 | **What was the result?** | | |
| | 1 - Positive | 3 | 1.7% |
| | 2 - Negative | 45 | 25.6% |
| v13 | **How did the media influence your decision making? The** | | |
| | 1 - ... helpful | 81 | 46.0% |
| | 2 - ... confusing | 47 | 26.7% |
| | 3 - I do not rely on the media as an information source. | 25 | 14.2% |
| | 4 - Free text | 23 | 13.1% |
| v14 | **How old are you?** | Mean 50.5 (SD 15), range 18-82 | |
| v15 | **What is your sex?** | | |
| | 1 - Female | 101 | 57.4% |
| | 2 - Male | 75 | 42.6% |
| | 3 - Other | 0 | 0.0% |
| v16 | **What is your nationality?** | | |
| | 0 - Missing | 0 | 0.0% |
| | 1 - Swiss | 147 | 83.5% |
| | 2 - German | 13 | 7.4% |
| | 3 - French | 1 | 0.6% |
| | 4 - Italian | 3 | 1.7% |
| | 5 - Liechtenstein | 0 | 0.0% |
| | 6 - Greater Europe | 4 | 2.3% |
| | 7 - Free text | 7 | 4.0% |
| v17 | **In which province do you live?** | | |
| | 1 - Bern | 108 | 61.4% |
| | 2 - Zürich | 12 | 6.8% |
| | 3 - Luzern | 10 | 5.7% |
| | 4 - Uri | 0 | 0.0% |
| | 5 - Schwyz | 1 | 0.6% |
| | 6 - Obwalden | 0 | 0.0% |
| | 7 - Nidwalden | 0 | 0.0% |
| | 8 - Glarus | 0 | 0.0% |
| | 9 - Zug | 2 | 1.1% |
| | 10 - Fribourg | 7 | 4.0% |
| | 11 - Solothurn | 3 | 1.7% |
| | 12 - Basel-Stadt | 2 | 1.1% |
| | 13 - Basel-Landschaft | 1 | 0.6% |
| | 14 - Schaffhausen | 0 | 0.0% |

For peer review only

| | | | |
|---|---|---|---|
| | 15 - Appenzell Ausserrhoden | 2 | 1.1% |
| | 16 - Appenzell Innerrhoden | 0 | 0.0% |
| | 17 - St. Gallen | 2 | 1.1% |
| | 18 - Graubünden | 3 | 1.7% |
| | 19 - Aargau | 9 | 5.1% |
| | 20 - Thurgau | 1 | 0.6% |
| | 21 - Ticino | 2 | 1.1% |
| | 22 - Vaud | 7 | 4.0% |
| | 23 - Valais | 0 | 0.0% |
| | 24 - Neuchâtel | 1 | 0.6% |
| | 25 - Geneva | 0 | 0.0% |
| | 26 - Jura | 0 | 0.0% |
| | 27 - I do not live in Switzerland | 3 | 1.7% |
| v18 | **What is your highest level of education?** | | |
| | 0 - Missing | 6 | 3.4% |
| | 1 - Tertiary education (university degree, college of education) | 120 | 68.2% |
| | 2 - Upper secondary education (High School Graduation, FMS, EZF, EBA) | 27 | 15.3% |
| | 3 - Lower secondary education/ obligatory schooling completed | 23 | 13.1% |
| | 4 - No formal education | | |
| v19 | **Are you currently...** | | |
| | 0 - Missing | 33 | 18.8% |
| | 1 - Employed | 106 | 60.2% |
| | 2 - Self employed | 24 | 13.6% |
| | 3 - Unemployed already before the current pandemic | 3 | 1.7% |
| | 4 - I lost my job during the COVID-19 period | 1 | 0.6% |
| | 5 - Studying or in an apprenticeship | 9 | 5.1% |
| v20 | **How much approximately do you earn per month? (net income in December 2019 including 1/12 of the 13th month salary.)** | | |
| | 0 - Missing | 29 | 16.5% |
| | 1 - Less that 4'000 CHF | 26 | 14.8% |
| | 2 - Between 4'001 und 6'000 CHF | 42 | 23.9% |
| | 3 - Above 6'001 CHF | 79 | 44.9% |
| v21 | **What type of health insurance do you have?** | | |
| | 1 - General | 68 | 38.6% |
| | 2 - Telemedicine - Modell | 12 | 6.8% |
| | 3 - GP - Modell | 83 | 47.2% |
| | 4 - Another alternative model | 8 | 4.5% |
| | 5 - No insurance | 5 | 2.8% |
| v22 | **In a second stage, we will interview individual participants of** | | |
| | 1 - Yes, I consent to be contacted. | 78 | 44.3% |
| | 2 - No, please, no more interviews. | 98 | 55.7% |
| | 3 - Free text | - | - |

For peer review only

Interview Guide: Coronatest.ch -Patients v 2

# Rapport

0Describe yourself (prompts; nationality, occupation, living arrangements, employment status)

# Accessibility

0How did you get to coronatest.ch website? (referred by …, online search). What is your understanding of an OFTT?

0Did you consult BAG- was the information supplied by BAG understandable to you? In what way was it useful.

0Was the online digital tool easy for you to access? explain why or why not (easy to find on homepage, length of tool, clear and easy to follow instructions, language, sequence, when did you use the tool Monday, Tuesday, weekend or during the week and why)

0What information or components would have helped you better -what do you suggest needs to be done to make such an online too more accessible.

# Utility as a reliable information source and decision making

0When you consulted the online digital tool; did you follow the recommended advice? Prompt (why and how socioeconomic status could have influenced the process)

0What made you follow the advice and recommendations? Prompt confirmation from friends, generally, the media influence your decision-making process?

0What made you disregard the advice and recommendations? (prompt for severity of symptoms, change of condition)

0We have noticed that people that earn below 4000 tend not to follow recommendations, why do you think it`s like this, what can be done?

# Utility in allaying fear and anxiety

0Describe how you felt after consulting the online digital tool with regards to feeling anxious and or confident that all was going to be well? (Did you feel reassured after visiting corontest.ch)

0If your fears were not allayed, how did you deal with your fears? prompt on what increased confidence, what allayed your anxiety)

For peer review only

# Illness and testing Experience

0Did you test for COVID-19, what test and experience? Did you experience COVID-19 symptoms? Explain

0Speaking of self-isolation. Please explain in more detail how you experienced this what worked and what did not work e.g. the need to go shopping, not going for a run or walk protecting others vs own needs, putting others first, dilemmas, challenges

0The road to recovery has been described by many as very cumbersome and long-what was your experience? Any psychiatric or other residual effects experienced-explain.

0What personal life lessons did you learn during this pandemic you would like to share and what personal changes do you foresee in future

0What health and health system related observations did you make and what changes do you fore see in future?

0What, socio-economic changes have you observed and do you foresee in the future as a result of COVID-19

# Utility in preventing cross infection

0If you consulted a GP; did you call ahead of time?

0How did your GP/ health care provider react when you told him or her you suspected that you had COVID-19?

# Recommendations

0In a future pandemic, what would you do?

0Is there any additional information you wish a site like corontest provides but was missing during COVID-19? What information did you search for but did not find?

0Are there other strategies (to allay fear, anxiety) you deem effective alone or in conjunction with online digital tools when faced with epidemics such as COVID-19 to make it accessible to the older generation?

0How can online tools like corontast be adapted to facilitate your decision making processes

For peer review only

STROBE Statement—checklist of items that should be included in reports of observational studies

| | Item No | Recommendation | Page No |
|---|---|---|---|
| **Title and abstract** | 1 | (*a*) Indicate the study's design with a commonly used term in the title or the abstract | 1 |
| | | (*b*) Provide in the abstract an informative and balanced summary of what was done and what was found | 3 |
| **Introduction** | | | |
| Background/rationale | 2 | Explain the scientific background and rationale for the investigation being reported | 5 |
| Objectives | 3 | State specific objectives, including any prespecified hypotheses | 5 |
| **Methods** | | | |
| Study design | 4 | Present key elements of study design early in the paper | 6 |
| Setting | 5 | Describe the setting, locations, and relevant dates, including periods of recruitment, exposure, follow-up, and data collection | 6 |
| Participants | 6 | (*a*) *Cohort study*—Give the eligibility criteria, and the sources and methods of selection of participants. Describe methods of follow-up<br>*Case-control study*—Give the eligibility criteria, and the sources and methods of case ascertainment and control selection. Give the rationale for the choice of cases and controls<br>*Cross-sectional study*—Give the eligibility criteria, and the sources and methods of selection of participants | 7 |
| | | (*b*) *Cohort study*—For matched studies, give matching criteria and number of exposed and unexposed<br>*Case-control study*—For matched studies, give matching criteria and the number of controls per case | |
| Variables | 7 | Clearly define all outcomes, exposures, predictors, potential confounders, and effect modifiers. Give diagnostic criteria, if applicable | 7 |
| Data sources/ measurement | 8* | For each variable of interest, give sources of data and details of methods of assessment (measurement). Describe comparability of assessment methods if there is more than one group | *8-9* |
| Bias | 9 | Describe any efforts to address potential sources of bias | 9 |
| Study size | 10 | Explain how the study size was arrived at | 8 |
| Quantitative variables | 11 | Explain how quantitative variables were handled in the analyses. If applicable, describe which groupings were chosen and why | 10 |
| Statistical methods | 12 | (*a*) Describe all statistical methods, including those used to control for confounding | 10 |
| | | (*b*) Describe any methods used to examine subgroups and interactions | 10 |
| | | (*c*) Explain how missing data were addressed | 10.11.12.13 |
| | | (*d*) *Cohort study*—If applicable, explain how loss to follow-up was addressed<br>*Case-control study*—If applicable, explain how matching of cases and controls was addressed<br>*Cross-sectional study*—If applicable, describe analytical methods taking account of sampling strategy | |


For peer review only - http://bmjopen.bmj.com/site/about/guidelines.xhtml

| | |
|---|---|
| (*e*) Describe any sensitivity analyses | 12 |

Continued on next page

For peer review only


For peer review only - http://bmjopen.bmj.com/site/about/guidelines.xhtml

| | | | |
|---|---|---|---|
| **Results** | | | |
| Participants | 13* | (a) Report numbers of individuals at each stage of study—eg numbers potentially eligible, examined for eligibility, confirmed eligible, included in the study, completing follow-up, and analysed | 11 |
| | | (b) Give reasons for non-participation at each stage | |
| | | (c) Consider use of a flow diagram | |
| Descriptive data | 14* | (a) Give characteristics of study participants (eg demographic, clinical, social) and information on exposures and potential confounders | 11 |
| | | (b) Indicate number of participants with missing data for each variable of interest | 11 |
| | | (c) *Cohort study*—Summarise follow-up time (eg, average and total amount) | |
| Outcome data | 15* | *Cohort study*—Report numbers of outcome events or summary measures over time | |
| | | *Case-control study*—Report numbers in each exposure category, or summary measures of exposure | |
| | | *Cross-sectional study*—Report numbers of outcome events or summary measures | |
| Main results | 16 | (*a*) Give unadjusted estimates and, if applicable, confounder-adjusted estimates and their precision (eg, 95% confidence interval). Make clear which confounders were adjusted for and why they were included | 12.13.14 |
| | | (*b*) Report category boundaries when continuous variables were categorized | |
| | | (*c*) If relevant, consider translating estimates of relative risk into absolute risk for a meaningful time period | |
| Other analyses | 17 | Report other analyses done—eg analyses of subgroups and interactions, and sensitivity analyses | |
| **Discussion** | | | |
| Key results | 18 | Summarise key results with reference to study objectives | 18 |
| Limitations | 19 | Discuss limitations of the study, taking into account sources of potential bias or imprecision. Discuss both direction and magnitude of any potential bias | 21 |
| Interpretation | 20 | Give a cautious overall interpretation of results considering objectives, limitations, multiplicity of analyses, results from similar studies, and other relevant evidence | 20 |
| Generalisability | 21 | Discuss the generalisability (external validity) of the study results | 21 |
| **Other information** | | | |
| Funding | 22 | Give the source of funding and the role of the funders for the present study and, if applicable, for the original study on which the present article is based | 24 |

*Give information separately for cases and controls in case-control studies and, if applicable, for exposed and unexposed groups in cohort and cross-sectional studies.

**Note:** An Explanation and Elaboration article discusses each checklist item and gives methodological background and published examples of transparent reporting. The STROBE checklist is best used in conjunction with this article (freely available on the Web sites of PLoS Medicine at http://www.plosmedicine.org/, Annals of Internal Medicine at http://www.annals.org/, and Epidemiology at http://www.epidem.com/). Information on the STROBE Initiative is available at www.strobe-statement.org.

For peer review only



For peer review only - http://bmjopen.bmj.com/site/about/guidelines.xhtml

BMJ Open

# Effects and utility of an online forward triage tool during the SARS-CoV-2 pandemic: a mixed method study and patient perspectives, Switzerland

| | |
|---|---|
| Journal: | *BMJ Open* |
| Manuscript ID | bmjopen-2021-059765.R1 |
| Article Type: | Original research |
| Date Submitted by the Author: | 01-Apr-2022 |
| Complete List of Authors: | Michel, Janet; Inselspital Universitätsspital Bern, Department of Emergency Medicine<br>Mettler, Annette; University of Bern, Department of Emergency Medicine<br>Stuber, Raphael; University of Bern, Department of Emergency Medicine<br>Müller, Martin; University of Bern, Department of Emergency Medicine<br>Ricklin, Meret; University of Bern, Department of Emergency Medicine<br>Jent, Philipp; University of Bern, Department of Infectious Diseases<br>Hautz, Wolf; University of Bern, Department of Emergency Medicine; University of Oslo<br>Sauter, Thomas; University of Bern, Department of Emergency Medicine |
| <b>Primary Subject Heading</b>: | Emergency medicine |
| Secondary Subject Heading: | Health policy, Qualitative research, Public health, Emergency medicine |
| Keywords: | COVID-19, Public health < INFECTIOUS DISEASES, QUALITATIVE RESEARCH, Telemedicine < BIOTECHNOLOGY & BIOINFORMATICS, EPIDEMIOLOGY |
| | |

SCHOLARONE™
Manuscripts

*I, the Submitting Author has the right to grant and does grant on behalf of all authors of the Work (as defined in the below author licence), an exclusive licence and/or a non-exclusive licence for contributions from authors who are: i) UK Crown employees; ii) where BMJ has agreed a CC-BY licence shall apply, and/or iii) in accordance with the terms applicable for US Federal Government officers or employees acting as part of their official duties; on a worldwide, perpetual, irrevocable, royalty-free basis to BMJ Publishing Group Ltd ("BMJ") its licensees and where the relevant Journal is co-owned by BMJ to the co-owners of the Journal, to publish the Work in this journal and any other BMJ products and to exploit all rights, as set out in our licence.*

*The Submitting Author accepts and understands that any supply made under these terms is made by BMJ to the Submitting Author unless you are acting as an employee on behalf of your employer or a postgraduate student of an affiliated institution which is paying any applicable article publishing charge ("APC") for Open Access articles. Where the Submitting Author wishes to make the Work available on an Open Access basis (and intends to pay the relevant APC), the terms of reuse of such Open Access shall be governed by a Creative Commons licence – details of these licences and which Creative Commons licence will apply to this Work are set out in our licence referred to above.*

*Other than as permitted in any relevant BMJ Author's Self Archiving Policies, I confirm this Work has not been accepted for publication elsewhere, is not being considered for publication elsewhere and does not duplicate material already published. I confirm all authors consent to publication of this Work and authorise the granting of this licence.*

For peer review only

# Effects and utility of an online forward triage tool during the SARS-CoV-2 pandemic: a mixed method study and patient perspectives, Switzerland

Janet Michel[1]*, Annette Mettler[1]*, Raphael Stuber[1], Martin Müller[1], Meret E. Ricklin[1], Philipp Jent[3], Wolf E. Hautz[1,2], Thomas C. Sauter[1]

**Affiliations:**
[1] Department of Emergency Medicine, Inselspital, University Hospital, University of Bern, Bern, Switzerland
[2] Centre for Educational Measurement, University of Oslo, Oslo, Norway
[3] Department of Infectious Diseases, Inselspital, University Hospital, University of Bern, Bern, Switzerland
* Co-first authorship

**Author Details**

**Corresponding Author:**
Janet Michel RN, BA Cur, MPH, PhD
Department of Emergency Medicine
Inselspital, University Hospital, University of Bern.
3010 Bern, Switzerland
E-Mail: janetmichel71@gmail.com
Phone: +41 326322111

**Author 2**
Annette Mettler
Department of Emergency Medicine
Inselspital, University Hospital, University of Bern.
3010 Bern, Switzerland
E-Mail: annette.mettler@insel.ch

**Author 3**
Raphael Stuber
Department of Emergency Medicine
Inselspital, University Hospital, University of Bern.
3010 Bern, Switzerland
E-Mail: raphael.stuber@students.unibe.ch

**Author 4**
Martin Müller
Department of Emergency Medicine
Inselspital, University Hospital, University of Bern.
3010 Bern, Switzerland
E-Mail: martin.mueller2@insel.ch

For peer review only

**Author 5**
Meret E. Ricklin
Department of Emergency Medicine
Inselspital, University Hospital, University of Bern.
3010 Bern, Switzerland
E-Mail: meret.ricklin@insel.ch

**Author 6**
Philipp Jent
Department of Infectious Diseases
Inselspital, University Hospital, University of Bern.
3010 Bern, Switzerland
E-Mail: philipp.jent@insel.ch

**Author 7**
Wolf E. Hautz
Department of Emergency Medicine
Inselspital, University Hospital, University of Bern.
3010 Bern, Switzerland
E-Mail: wolf.hautz@insel.ch

**Author 8**
Thomas C. Sauter
Department of Emergency Medicine
Inselspital, University Hospital, University of Bern.
3010 Bern, Switzerland
E-Mail: thomas.sauter@insel.ch

**Word count 5 600**

**Key words**

online forward triage tool (OFTT), effects, utility, SARS-CoV-2, COVID-19, pandemic

For peer review only

For peer review only

# ABSTRACT

**Introduction:** Online forward triage tools (OFTT) are being widely used during this COVID-19 pandemic. The effects and utility of such tools however, have not been widely assessed.
Objective**:** To assess the effects (quantitatively) and the utility (qualitatively) of a COVID-19 OFTT in a pandemic context, exploring patient perspectives as well as eliciting recommendations for OFTT improvement.
**Methods**: We employed a mixed-method sequential explanatory study design. Quantitative data of all users of the OFTT between March 2$^{nd}$, 2020 and May 12$^{th}$, 2020 were collected. A follow-up survey of people who consented to participation was conducted. Secondly, qualitative data was collected through key informant interviews (n=19) to explain the quantitative findings, as well as explore tool utility, user experience and elicit recommendations.
**Results:** In the study period, 6,272 users consulted our OFTT; 40.2% (1626/4049) would have contacted a healthcare provider had the tool not existed. 560 participants consented to a follow-up survey and provided a valid e-mail address. 31.4% (176/560) participants returned a complete follow-up questionnaire. 84.7% (149/176) followed the recommendations given. 41.5% (73/176) reported that their fear was allayed after using tool. Qualitatively, seven overarching themes emerged namely i) accessibility of tool, ii) user-friendliness of tool, iii) utility of tool as an information source, iv) utility of tool in allaying fear and anxiety, v) utility of tool in medical decision making vi) utility of tool in reducing the potential for onward transmissions and vii) utility of tool in reducing health system burden.
**Conclusion:** Our findings demonstrated that a COVID-19 OFTT does not only reduce the health system burden, but can also serve as an information source, reduce anxiety and fear, reduce cross infections and facilitate medical decision making.

**Word count 277**

**Strengths and limitations**

Many online tools have been developed during the COVID-19 pandemic. The effects and utility of these tools however have not been assessed.

- *Coronatest.ch* was one of the first COVID-19 OFTTs in Switzerland. Our study could become the base line for studies that assess the effects and utility of such online tools. The identified themes namely i) accessibility of tool, ii) user-friendliness of tool, iii) utility of tool as an information source, iv) utility of tool in allaying fear and anxiety, v) utility of tool in decision making (test or not to test), and vi) utility of tool in reducing onward transmission-cross infection, vii) utility of tool in reducing health system burden, could serve as a framework for assessing OFTT utility (follow-up paper). The mixed method sequential explanatory design gave us a better understanding of OFTTs, their effects measured quantitatively and utility explained with the aid of qualitative findings. We did not simply report the effects but could also explain why the results were that way, generating a holistic picture of the phenomenon.
- The selection of the participants in our study carries the risk of a selection bias. Perspectives of those that do not use online tools are missing and should be explored in further studies. In addition, only a limited number of OFTT users took part in our study. This selection bias cannot, to the best of our knowledge, be prevented due to data

For peer review only

protection regulations which impose a voluntary participation and prohibit a technically possible automatic tracking of participants. Another way to avoid this possible selection bias would be to make the use of such a tool conditional on participation in the study. We have deliberately decided against this procedure for ethical reasons, in order to make our OFTT accessible to as many users as possible and to keep barriers as low as possible. In addition, mandatory entry of personal data in OFTT for study purposes would also discourage individuals from using the tool and thus trigger a new bias. Our comparison of overlapping questions between the OFTT and the follow-up survey can at least help to estimate the similarities within the two groups. For both questions, the percentages are comparable and can help in estimating the similarity of the groups.

- Another limit of our study is the relatively long duration between the use of tool and the qualitative interviews. This could have introduced a certain degree of recall bias.
- As with all online tools, we cannot confirm the accuracy of the data entered. In particular, we cannot say for sure whether the OFTT users used the tool to assess own symptoms or for other reasons, such as curiosity, fear or uncertainty about how to deal with the novel infection. Likewise, multiple use, trial runs or use of tool by a health care worker on behalf of patients, relatives and friends are all possible. Socio-economic status might have introduced a selection bias in our study since most of the participants had a higher education. Income emerged not to be a good proxy for assessing socio-economic status. Other instruments, apart from income are therefore needed to assess socio-economic status. Additionally, an on online assessment cannot fully replace a (polymerase chain reaction) PCR test as some asymptomatic people might be positive and those with COVID-19 specific symptoms might be suffering from a different disease.[5]
- In our mind, the data still sheds light on the effects and utility of such an online tool and the recommendations given could guide other OFTT developers as the third wave sweeps across Europe. As the study was conducted with a specific OFTT, transferability of our results to other OFTTs is not necessarily a given. Given the limited evidence on the use of OFTTs, the results, in particular the qualitative component of the study, could be of value to other OFTT developers, with particular regards to utility and accessibility issues. Further studies with other OFTTs outside the COVID-19 context are recommended so as to increase transferability and improve the utility of OFTTs in the current third wave, future pandemics and other health care settings.

For peer review only

# INTRODUCTION

The number of COVID-19 cases across the globe has surpassed 25 million and incident rates are again on the rise as many European countries experience subsequent waves.[1–4] Many people are seeking reliable information, recommendations on testing and management of COVID-19 as well as reassurance, adding to the health system burden. Online forward triage tools (OFTT) are being widely used during this COVID-19 pandemic context[5–8] as misinformation and worry in the population abound. There is evidence from an earlier 2009 H1N1 influenza pandemic, that online tools are effective and practical in reducing the health system burden.[9,10] There is also emerging evidence of this nature from the COVID-19 context. [6,11–14] For example, OFTTs help reduce exposure of worried but uninfected and infected persons, through avoidance of hospitals and doctors' offices – enabling patients to access recommendations of what to do, from the comfort of their own homes.[10,11]

Using OFTTs is relatively easy to the computer literate. People respond to questions and upon completion, recommendations are given, e.g., isolate, test, do not test etc. Existing evidence on the effects and utility of OFTTs differ with possible implications on the quality of the symptom assessment [5]. According to literature, the reasons patients use symptom checkers or OFTTs are i) to understand the causes of their symptoms (76%), ii) to determine whether or not to seek care (33%), and iii) where to seek care (21%).[15] There is also evidence that patients that have previously experienced a diagnostic error are more likely to use OFTT to search for where to seek care [15] than those that have not.

**Challenges with OFTT use and research gap**

In the European Union, 87% of people aged 75 years and above have never been online according to a recent survey.[16]That means the elderly, may be less inclined to use online tools if not computer literate. This in turn shuts the elderly out from society, increasing isolation and loneliness, not to mention the missed health benefits [10]. The digital divide is real [17]. How can digital tools be designed to be more inclusive?[18] Information on factors influencing the use of OFTTs is scant and the validation of COVID-19 OFTTs like other OFTTs, seems neglected. [15,19] That makes the quality assessment of these tools paramount [5] as evidence on effects and utility of OFTTs is limited.

**The aim of this study**

This study aimed at assessing the effects (quantitatively) and the utility (qualitatively) of a COVID-19 OFTT during a pandemic context in Switzerland, exploring patient perspectives and derive recommendations for tool improvement. We hypothesized that an OFTT adequately reduces patient visits to the health care system and consequently reduces the health system burden. We further explored qualitatively, for emergent themes, capturing the tool utility to this population.

For peer review only

# METHODS

## Study design and participants

We employed a mixed-method sequential explanatory design to study the utility of the OFTT and the effects of using such a tool. The rationale for mixing both kinds of data within one study is that neither qualitative nor quantitative methods, are sufficient by themselves, to capture details of a phenomenon. In combination, they complement each other, taking advantage of the strengths of each. As in sequential explanatory designs, quantitative data collection was done first, as a major component of our study to inform qualitative interviews, see Figure 1.

**About here Figure 1: Mixed-Methods Sequential Explanatory Study Design.**

## Online forward triage tool description and setting

The working group e-emergency medicine at the emergency department (ED), Inselspital University Hospital Bern, together with the Department of Infectious Diseases, Inselspital University Hospital Bern, developed an online forward triage tool (OFTT) which was made available online (*coronatest.ch*). To the best of our knowledge, this was one of the first COVID-19 OFFTs set up in the German speaking part of Switzerland. In a skip-logic, the OFTT displayed the current test recommendations of the Federal Office of Public Health (FOPH) on whether someone needed testing for COVID-19 or not. No diagnosis was provided by the OFTT.

The questions and the content of the OFTT represented the official FOPH recommendations at the time. Thus, the OFTT was comparable in content to other OFTTs in Switzerland, which were based on the FOPH guidelines within that time period. One additional non-mandatory question, which did not affect the result, was integrated in our OFTT from the 11$^{th}$ March 2020, namely the question "What would you do if this online test did not exist?".

There were two possible outcomes of the OFTT: "According to the criteria of the Federal Office of Public Health (BAG), one meets or does not meet the criteria for a test for an infection with the coronavirus, COVID-19". The results page was linked to the FOPH's official behavioural recommendations and recommendations for the testing process. The average time to complete the assessment was 75sec.

## OFTT triage

Details on the structure of the OFTT as well as screen shot are published in a separate quantitative paper[20]. The Federal office of public health (FOPH) national COVID-19 Swiss testing criteria were transferred into a digital decision tree and adjusted promptly after the criteria were adapted by the FOPH. During the first phase of the pandemic, the recommendations for testing or not testing were mainly based on contact with an infected person or a visit to a risk area and were then changed during the course of the pandemic to a testing regime based on risk groups (healthcare professionals, patients>65 years and patients with pre-existing conditions). With the general availability of the tests, the test recommendations were extended to all symptomatic patients and our OFTT became obsolete. Unlike other triage techniques performed on emergency patients, the aim of the OFTT was not to make a COVID-19 diagnosis, assess the risk of severe COVID-19 progression or recommend treatment. See Fig 2 below.

For peer review only

**About here Fig 2: OFTT triage**

## Quantitative data

### Research participants and data collection

Participants included all users above the age of 18 that used the OFTT between March 2nd, 2020 and May 12th, 2020. In this timeframe, the recommendations on COVID-19 frequently changed in Switzerland and there was an initial lack of testing reagents and capacity as well as the risk of overburdening the healthcare system. During the first few weeks of the pandemic, the Federal Office of Public Health (FOPH) recommended testing only for symptomatic patients after travel to high-risk countries (e.g., Italy and China) or symptomatic contacts of coronavirus patients. In weeks that followed (as from the 20th March 2020), the strategy changed to testing of high-risk groups (older than 65 years, pre-existing conditions, and healthcare workers). The countries and risk groups were regularly adjusted according to the spread of the virus and the findings about risk groups but also the availability of testing capacity.

Due to the rapid spread of the virus in Switzerland, and broadly available testing capacities, a universal test recommendation was made by the Federal Office of Public Health (FOPH)- on April 27th, 2020. All symptomatic individuals were eligible to test. With this recommendation, our OFTT provided less benefit to the user and was finally removed on May 12th 2020 from the website paving the way to a second generation OFTT.

To minimise the barrier to the use of the OFTT and for legal data protection reasons, no personal data was collected within the OFTT. Further data on the users of the OFTT was collected in a second step, from participants who gave their explicit consent and provided their email addresses to be contacted. This also made it possible to investigate the adherence to recommendations and the test results. A non-mandatory additional question was built into the OFFT from 11th March 2020.

A pretested online questionnaire (see supplementary info) was used to assess the

i) utilization of the OFTT, including way of referral to the tool, reasons for use and information searched,
ii) additional factors, including influence of the media and influence of the OFTT on fear and anxiety.

The database used is compliant with Swiss laws on the collection of personal health related information. The follow-up questionnaire is available as supplementary information. Due to ethical reasons, we included the option "not want to answer" as a choice in the questionnaire for the socio-demographic data, in case the respondent did not want to give a statement on this specific sensitive topic.

The qualitative interviews were conducted with purposefully selected key informants who gave their consent during the survey (see below).

### Data analysis

Quantitative data was analysed in Stata® 16.1 (StataCorp, The College Station, Texas, USA). Descriptive statistics for all variables as mean and standard deviation or frequency as determined by the type and distribution of the data were computed. Categorical variables between two groups were compared using Chi-square statistics and the distribution of continuous variables were compared using Wilcoxon rank sum test.

To assess the risk of selection bias and to estimate the similarity of the groups, we compared responses to overlapping questions within the OFTT and the follow-up survey.

For peer review only

## Qualitative data

To explain the quantitative results, we explored the experience of tool use by the patients qualitatively. Following quantitative data analysis, an interview guide was created and adapted iteratively.

## Purposeful and quota sampling

We purposefully sampled participants from those that had firstly, utilized our OFTT, secondly, had taken part in the follow-up survey and thirdly, had consented to a follow-up interview. We included participants of all age groups (quota) to ensure inclusiveness.

## Sample Size

Many experts suggest saturation as central to qualitative sampling [20]. In this study we aimed for both data saturation and rich and detailed narratives and achieved this with 19 key informants from all age groups (see Table 1).

## Data collection

Due to COVID-19 concerns, video rather than face to face interviews were held with most participants in September 2020. A combination of video and telephonic interviews were conducted with three participants who had technical challenges and a telephone only interview was held with one lady, aged above 65, who had no computer access. Three face to face interviews were held with three key informants: one that was a hospital health care worker, and two key informants who worked close to Bern university hospital. A semi-structured interview guide informed by the quantitative results was used (see supplementary info). This was adapted iteratively throughout the data collection period. Two qualitative researchers sat in each session fielding questions in turns. All interviews were conducted in German by two researchers fluent in both English and German. The interviews lasted between 45 minutes to one and a half hours. Two audio-recorders were used in each session. All participants gave individual written consent as well as oral consent to the recording at the beginning of each session. See Table 1 for summary of Key Informants.

For peer review only

**Table 1. Key Informant summary**

| Age-group | Males | Females | Total |
|---|---|---|---|
| 18-29 | 1 | 2 | **3** |
| 30-45 | 2 | 2 | **4** |
| 46-64 | 3 | 4 | **7** |
| 65+ | 4 | 1 | **5** |
| **Total** | **10** | **9** | **19** |

## Data analysis

Audio recordings were transcribed, analysed and triangulated with quantitative data results. Qualitative narratives were obtained to explain quantitative results as well as to explore utility of OFTT to patients as well as elicit recommendations to make online tools more useful and inclusive. A grounded theory approach was utilized. Concepts were identified from collected data and compared iteratively. These concepts were grouped into categories and culminated into the identified themes.

**Measures to ensure trustworthiness of data:** To ensure dependability, data collection and analysis were performed iteratively, continuously adjusting our interview guide to capture newly emerging themes. Throughout data collection, two qualitative researchers kept reflexive journals and debriefed at the end of each interview. To ensure transferability, a thick description of participants, context and data collection process has been outlined. Data was managed and analysed with the aid of MAXQDA2018.

## Ethics approval

The local ethics committee of the Canton of Bern, Switzerland, deemed this project a quality evaluation study and waived the need for full ethical review (Req-2020-00289) on the 23rd of March 2020.

## Patients and Public Involvement statement

Patients and public were not involved in the design, conduct, reporting or dissemination of this research since the OFTT was set up as an emergency response to the pandemic.

For peer review only

# RESULTS

## Quantitative results

In total, n=6,272 completed assessments of the OFTT were recorded on the website during the study period from March 2nd, 2020 to May 12th, 2020. This question asked OFTT users what they would have done had the OFTT not existed. The question was answered by 97.6% (3953/4049) of the users as follows: 40.2% (1626/4049) would have contacted the GP or visited a hospital had the tool not existed; furthermore, 16.4% (665/4049) would have contacted a hotline.

In the OFTT, 25.6% (1,608/6272) of assessments received a recommendation to test for COVID-19 during the study period. In the follow-up survey question, "Did the online tool recommend you to test for COVID-19?" -31.8% (56/176) answered, yes.

In the OFTT, 13.2% (564/4270) of OFTT users reported being over 65 years of age. The variable age was only included and mandatory during some phases of the study period in accordance with the FOPH guidelines, that changed frequently. This resulted in 4270 assessments with data on age. In the follow-up survey, 17.6% (31/176) reported being over 65 years.

A link to the online follow-up questionnaire was sent to 560 participants that consented to a follow-up survey by providing a valid e-mail address. The online questionnaire was filled out by 37.9% (212/560) of the participants; 31.4% (176/560) completed the whole questionnaire and were included in the analysis (all 22 questions-see supplement). An overview of socio-demographic characteristics of participants of the follow-up survey are presented in Table 2.

For peer review only

**Table 2. Socio-demographic table of participants of follow-up survey**

| | Total | (n=176) | Female | (n=101) | Male | (n=75) | P-value* |
|---|---|---|---|---|---|---|---|
| **Age [mean, SD]** | 50.1 | [±15.4] | 45.9 | [±14.1] | 55.7 | [±15.4] | <0.001 |
| **Education** | | | | | | | |
| Not want to answer | 6 | [3.4] | 3 | [3.0] | 3 | [4.0] | |
| University | 120 | [68.2] | 67 | [66.3] | 53 | [70.7] | |
| Higher secondary school | 27 | [15.3] | 17 | [16.8] | 10 | [13.3] | |
| Lower secondary school | 23 | [13.1] | 14 | [13.9] | 9 | [12.0] | 0.871 |
| **Income per month** | | | | | | | |
| Not want to answer | 29 | [16.5] | 17 | [16.8] | 12 | [16.0] | |
| <4000 CHF | 26 | [14.8] | 20 | [19.8] | 6 | [8.0] | |
| 4000 - 6000 | 42 | [23.9] | 27 | [26.7] | 15 | [20.0] | |
| >6000 | 79 | [44.9] | 37 | [36.6] | 42 | [56.0] | 0.037 |
| **Work** | | | | | | | |
| Not want to answer | 33 | [18.8] | 14 | [13.9] | 19 | [25.3] | |
| Employed | 106 | [60.2] | 64 | [63.4] | 42 | [56.0] | |
| Self-employed | 24 | [13.6] | 13 | [12.9] | 11 | [14.7] | |
| Unemployed | 3 | [1.7] | 3 | [3.0] | 0 | [0.0] | |
| Lost work (Covid-19) | 1 | [0.6] | 1 | [1.0] | 0 | [0.0] | |
| Student/trainee | 9 | [5.1] | 6 | [5.9] | 3 | [4.0] | 0.236 |
| **Insurance** | | | | | | | |
| Don't know | 5 | [2.8] | 3 | [3.0] | 2 | [2.7] | |
| General | 68 | [38.6] | 39 | [38.6] | 29 | [38.7] | |
| Telemedicine | 12 | [6.8] | 6 | [5.9] | 6 | [8.0] | |
| GP | 83 | [47.2] | 47 | [46.5] | 36 | [48.0] | |
| Other | 8 | [4.5] | 6 | [5.9] | 2 | [2.7] | 0.859 |
| **Nationality** | | | | | | | |
| Not want to answer | 1 | [0.6] | 1 | [1.0] | 0 | [0.0] | |
| Switzerland | 147 | [83.5] | 80 | [79.2] | 67 | [89.3] | |
| Germany | 13 | [7.4] | 8 | [7.9] | 5 | [6.7] | |
| French | 1 | [0.6] | 0 | [0.0] | 1 | [1.3] | |
| Italy | 3 | [1.7] | 2 | [2.0] | 1 | [1.3] | |
| Other Europe | 4 | [2.3] | 3 | [3.0] | 1 | [1.3] | |
| Other | 7 | [4.0] | 7 | [6.9] | 0 | [0.0] | 0.202 |

For peer review only

* Chi-squared for categorical variables and Wilcoxon rank sum test for continuous variables; data are total number and percentage if not mentioned otherwise

The survey revealed that 84.7% (149/176) followed the tool recommendations and stayed at home thereby reducing the work-load of GPs and hospitals. Information about the utilization of the OFTT, specifically which information was searched for, how subjects found the tool, and information about satisfaction with the tool is presented in Table 3.

**Table 3. Online forward triage tool use**

| | **Total (n=176)** | **[%]** |
|---|---|---|
| Information searched | | |
| Information on COVID-19 symptom | 97 | [55.1] |
| How to cope with symptoms | 4 | [2.3] |
| To know when to consult a doctor | 36 | [20.5] |
| To know more on testing criteria | 32 | [18.2] |
| To know where to test | 7 | [4.0] |
| Mode of referral | | |
| Referral by family doctor | 9 | [5.1] |
| Online search | 113 | [64.2] |
| Recommendation by peers | 17 | [9.7] |
| Hotline | 2 | [1.1] |
| Other | 35 | [19.9] |
| Satisfaction with information | | |
| Helpful | 154 | [87.5] |
| Not comprehensive | 17 | [9.7] |
| Not clear | 5 | [2.8] |

For peer review only

We present additional factors that may have influenced how individuals coped during the coronavirus pandemic, their use of the OFTT and adherence to OFTT recommendations. Overarching topics that were asked included the influence of the media, fear and uncertainty, and reasons for adherence to the recommendation (see table 4). All questions and answers from the follow-up questionnaire are attached. See supplement 1.

For peer review only - http://bmjopen.bmj.com/site/about/guidelines.xhtml

**Table 4. Additional factors**

| | Total (n=176) | [%] |
|---|---|---|
| Estimated influence of media | | |
| Helpful | 81 | [46.0] |
| Confusing | 47 | [26.7] |
| No trust in media as source of information | 25 | [14.2] |
| Other | 23 | [13.1] |
| Influence of OFTT on fear and anxieties | | |
| Reassured | 73 | [41.5] |
| No reassurance | 13 | [7.4] |
| Increased fears and anxieties. | 6 | [3.4] |
| Not worried before OFTT use | 84 | [47.7] |
| Reasons for following the recommendation (n=149) | | |
| Trust in tool | 60 | [40.3] |
| Information congruent with media | 20 | [13.4] |
| Comparison with FOPH recommendation | 53 | [35.6] |
| Reassurance by others | 7 | [4.7] |
| Other | 9 | [6.0] |

For peer review only

**Qualitative findings**

Seven overarching themes on the utility of the OFTT emerged during the qualitative interviews. These are used to structure the report of our findings, i.e., i) accessibility of the tool, ii) user-friendliness of the tool, iii) utility of the tool as an information source, iv) utility of the tool in allaying fear and anxiety, v) utility of the tool in decision making (test or not to test), vi) utility of the tool in reducing onward transmission-cross infection, and vii) utility of the tool in reducing health system burden. The qualitative findings are summarised in Table 5.

**Table 5. Summary of qualitative themes**

| Theme | Category | Unit meaning |
|---|---|---|
| **Accessibility** | Online search | Appeared but not on the top of google search<br>Advertise tool in future |
| | Unreachable for some | Include telephonic services to reach the elderly<br>Tool buddies |
| **Utility as a reliable information source** | COVID-19 Symptoms | Cough was a main symptom<br>Symptom description like type of cough and severity of fever etc. was not possible<br>Test or do not test decision was arbitrary-how the decision was arrived at was not clear e.g., 95% probability test or 5% probability do not test |
| | Testing info and centres missing | Information on when to call doctor was not clear e.g., fever above 39 degrees for 4 days -call doctor<br>List of where to test and contact numbers were missing |
| **Utility in decision making** | Followed recommendations | Trust- the university hospital is a trusted institution |
| | Did not follow recommendations | Fear of a positive result and the resultant consequences<br>Cost of test<br>Test shortage<br>GP refusing patients to test -hysteria |
| **Utility in allaying fear and anxiety** | Reassured some | Fear and anxiety allayed after tool use |
| | Person contact | An online tool is still an online tool - recommendations seen as not having a lot of weight<br>A talk with a general practitioner (GP)-debriefing after tool use could have put them at ease |
| | Testing | Testing in itself is reassuring -make test available to all who are anxious |
| | Friends and family as a resource | Many relied on family and friends to deal with fear- social circle still a major source of support |
| | Increased anxiety in some | |

For peer review only

| | | |
|---|---|---|
| | | High risk label unsettled some |
| **Utility in reducing health system burden** | Many stayed at home | Recommendations followed- stay at home<br>Some called Insurance companies |
| **Utility in reducing onward transmission** | Call GP before a visit | Most called GP ahead of visit |
| **Systems thinking** | Utility of tool is dependent upon other health system and societal components<br><br>Fear of a positive test<br>-rather not know | Participants told by tool to test only to be told that there are no tests (shortages)<br>Fear of a positive test<br><br>Media misinformation of painful test influenced some not to test-work with media<br><br>Economic factors like cost of test influenced some not to test<br>A new life-threatening disease in a population is associated with psycho-social and behavioural issues that need to be taken into account |

**Theme 1: Accessibility of the tool**

The accessibility of the tool emerged as very important. Many participants suggested to advertise the tool to make it more accessible as revealed below:

> *"I did not know of the existence of tool (an accidental internet search led the key informant to the tool). Please advertise tool on TV and to Insurance companies."* -Key Informant 15

The older people seem willing to embrace technology and were prepared to use it. However, they stated that they needed help with practical application at times as revealed below;

> *"Provide telephone services for the elderly and a contact person, a GP so one can ask questions if unsure."* -Key Informant 14

**Theme 2: User-friendliness of the tool**

Most participants could not remember the tool immediately due to the time lapse from the tool usage to interview. After being shown the tool once again, the header only, many cited it as having been easy and simple to follow with the language being clear and the length acceptable.

**Theme 3: Utility of the tool as an information source**

The novel nature of COVID-19 infection left many scrambling for knowledge of the disease. Many health care providers were inundated with phone calls. One participant said the following;

review only

*"The tool provided information on symptoms but did not have a list of testing centers. The recommendations said call GP before visit but there was no number to call."* -Key Informant 1

*"Telemedicine could play a better information spreading role – media spread fear and misinformed people for example mask use vs no mask."* -Key Informant 15

**Theme 4: Utility of the tool in allaying fear and anxiety**

Many participants interviewed reported being reassured after tool use. Others cited being more anxious after tool use due to terminology and language and many suggested that a person, a doctor be available after tool use for closure. Participants revealed the following;

*"Wording of tool could be adapted – a friend aged 65, a diabetic, became depressed after using tool and getting the high-risk patient classification. He needed a psychiatrist to cope. Rather ask how are you, do you take any medication, which ones? Mentioning conditions seem to increase anxiety."* -Key Informant 17

*"I felt discriminated against by tool-differentiate between a health 73-year-old with no chronic illnesses and a 50-year overweight diabetic."* -Key Informant 13

**Theme 5: Utility of the tool in decision making process (to test or not to test)**

Many participants cited trust in our university hospital (Insel) as one of the main reason participants followed the recommendations. Some participants revealed the following;

*"Insel has a good name and trusted the tool."* -Key Informant 16

*"Coordination is needed for FOPH and Insel to speak in one voice."* -Key Informant 17

Juxtaposed and not necessarily contradicting the quantitative survey, where trust was reported as the main reason for following the recommendations, most of the participants cited shortages of tests, improved symptoms, cost of test, misinformation that the test was painful and fear of a positive result as reasons for not testing. Of utmost importance were GPs who viewed the test request by online tool users as being hysteric. Below is what some participants said:

*"I read scientific papers to inform oneself and then decided."* -Key Informant 8

*"Remember recommendations from an online tool have less weight than recommendations from a doctor – there is no person behind this and so many might have taken the tool and went further to contact own GP"*- Key Informant 8

*"I wished to see an algorithm that said something like, "the probability of you having COVID-19 is 75% test or 25% do not test."*-Key Informant 5

**Theme 6: Utility in reducing the potential for onward transmission- cross infection**

The tool recommended all participants to call the health care provider ahead of visit and most of them did. A reason some participants might not have called the testing centres ahead of a visit could be that the tool itself did not provide a list of contact numbers-a short coming that was rectified in the second generation OFTT.

For peer review only

**Theme 7: Utility of tool in reducing health system burden**

Social distancing, isolation and quarantine were among the recommendations made to reduce the spread of COVID-19. Most of the participants stayed at home. One participant said the following;

> *"I followed recommendations and stayed at home. However, home testing should be provided if people should stay at home. Engage Spitex [organization for outpatient and home-based care in Switzerland] in future pandemics and work with them."* -Key Informant 6

For peer review only

# DISCUSSION

This study quantitatively assessed the effects and confirmed the utility (qualitatively) of a COVID-19 online forward triage tool by exploring patient perspectives. We further elaborate on areas for improvement as well as share lessons learned for policy makers. Qualitatively, seven overarching themes emerged namely i) accessibility of tool, ii) user-friendliness of tool, iii) utility of tool as an information source, iv) utility of tool in allaying fear and anxiety, v) utility of tool in decision making (test or not to test), vi) utility of tool in reducing the potential for onward transmissions (preventing cross infection) and vii) utility of tool in reducing health system burden.

*Accessibility of OFTT*

One of the objectives of our OFTT was to provide an easily accessible, reliable and up to date information platform for professionals and the public. The tool was not advertised commercially; hence it did not appear at the top of the google search and many participants cited coming across the tool accidentally. Information about the tool was only disseminated via the hospital website and hospital communication to local doctors.

Despite the above -mentioned shortcoming, our findings revealed that the tool was accessible to both genders and all age groups including the elderly. In line with other studies,[21] the elderly seem ready to embrace online tools, contradicting other studies.[10,17] Contradicting our findings, one study revealed that it's the young and highly educated patients that tend to use symptom checkers or OFTTs.[22]

Despite the revealed readiness of the elderly to embrace technology, key informants suggested keeping the use of telephonic services for the elderly as an option in telemedicine. Further supporting these findings, nurse triage lines (telephone) have been proven effective in this COVID-19 pandemic context in the US and in Canton Vaud, Switzerland.[10,23] Others suggested having a list of tool buddies reachable by phone, that links people that have used the tool before and are willing to be contacted by a new user, that might be experiencing challenges in using the OFTT. With regards to reaching the low education and low-income group, additional studies need to be done as those who earned less than CHF 4000 were not necessarily lowly educated but PhD and post doc students, concurring with findings elsewhere.[24]

*User-friendliness of OFTT*

Most of the participants could not recall tool, but after showing them tool header only, many cited tools as user-friendly, easy, with a clear language and an acceptable length, concurring with a study that was conducted elsewhere.[25] In support of our findings, online tools have been shown to be risk averse as compared to health care professionals and the users have expressed high levels of satisfaction.[22] The optimal amount of time spent filling in OFTT questionnaires nor the optimal number of questions an OFTT should ask in general, is still unclear[26] and warrants further studies.

*Utility of OFTT as an information source*

Overall, the tool was very useful in providing information on signs and symptoms. Information on where to test (list with contact numbers), how to self-care, when to contact a GP were cited by some as shortcomings and ought to be included to make the tool comprehensive in future. Information challenges with OFTTs have also been reported elsewhere.[27,28] This finding underlines the need to have an option to talk directly to a GP after OFTT use so as to debrief.

Further information or links to comprehensive and reliable sources with information on how to self-care and when to contact a GP or health care centre emerged as gaps that need to be incorporated in COVID-19 OFTTs so as to increase their utility as information sources. The

majority of our participants were highly educated, and this segment of the population seems to inform itself, by consulting a variety of scientific sources as well as keeping abreast with the FOPH announcements. In the context of a novel infection, where guidelines change quickly and continuously, the credibility of the tool to the highly educated, could be enhanced by stipulating sources of information and referencing and dating the FOPH criteria informing the tool.

*Utility of OFTT in allaying fear and anxiety*

For most of the participants, the tool was effective in allaying their fear and anxiety. Many wished a human presence, a doctor to debrief with after the online tool use as mentioned above. There was however, a downside for some that felt labelled as being high risk. For this group, the tool had a negative effect and increased their anxiety. Other studies have revealed similar effects.[29,30] This raises the issue of language and terminology use in such tools. Bearing in mind that COVID-19 is a novel condition, not well understood and considered fatal, the impact of a high-risk label should not be underestimated, including discrimination. Concurring with our findings, COVID-19 stigma has been reported elsewhere [31]. Many participants reported fear of a positive test result and the consequences thereof, concurring with findings from elsewhere. [32,33] Further concurring with our findings, lasting psychological consequences that last beyond the COVID-19 infection itself have also been revealed.[31] This raises the question of psychological readiness to deal with such a diagnosis. Emerging studies have reported COVID-19 patients as having psychiatric related conditions post infection, further concurring with our study.[34,35]

*Utility of OFTT in facilitating decision making*

The tool was useful in assisting patients in decision making particularly not to test. Trust in the institution proved pivotal as many followed recommendations simply because they trusted the source of the tool, our university hospital. Studies elsewhere concur with our findings.[36,37] On the other hand, some of those that got the recommendation to test did not do so due to a myriad of reasons as revealed above. In addition, the cost of the test (CHF 180 at the time), shortages of tests and fear of a positive result and the resultant consequences of isolating, stigma etc. further influenced decisions not to test. A low income was found not to be a reliable socio-economic status proxy in our study. Most low-income participants were PhD students and post-docs who cited various reasons for not following recommendations. Many told us how they sought and read scientific evidence to inform themselves and this, rather than the recommendations, guided their decision making. In line with our findings, salary is not a good proxy for socio-economic status among online tool users.[24] A shortcoming in this regard, was the missing information on how the tool arrived at the recommendation to test or not to test e.g. algorithm used[19] something some key informants wished to know. The issue of safety concerns with regards to specificity of digital tool algorithms has also been reported elsewhere.[38]

*Utility of OFTT in preventing onward transmission- cross infection*

The tool proved useful in preventing cross infection concurring with findings elsewhere.[19] Most participants who were told to stay at home did so, reducing mobility and exposure. Most of the participants called the GP practice ahead of time. That gave the GP practices time to ensure that the suspect patient did not mix with other patients, thereby reducing the potential for onward transmission (cross infection).[19]

For peer review only

*Utility of OFTT in reducing health system burden*

Our primary hypothesis was that such an OFTT reduces the health system burden. Most of the participants who used the tool would have called their GP or visited the hospital. OFTT use effectively kept these worried participants at home and out of the doctors' offices and hospitals, effectively reducing the health system burden. Contradicting our findings, research from elsewhere has produced inconclusive and sometimes contradicting evidence.[28,39] Further studies in different contexts are therefore called for. Further contradicting our findings,, another study reported that symptom checkers` triage capabilities are not greater than that of an average lay person.[40] In fact the convenience of telemedicine has also been associated with increased utilization of services, increasing work load and health care spending.[41]

**Recommendations and lessons learned**

Our study demonstrated the effects and utility of a COVID-19 OFTT. The assessment of an OFFTT is important but not without challenges. Below are some of the lessons worth sharing with both health care providers and policy makers as subsequent waves sweep across Europe;

- Most of the participants had challenges remembering the tool. Immediate feedback e.g., in one minute, please rate this tool, or three open questions; please tell us how useful this tool was with regards to i) accessibility of tool, ii) utility of tool as an Information source, ii) utility of tool in facilitating your decision making could be more effective. Data protection concerns and the need to keep barriers to use as low as possible, could stand in the way of this approach.
- The tool simply instructed patients to test or not to test, an arbitrary decision, without shedding light on how the decision was made. Patients wish to see an algorithm that says something like, "*the probability of you having COVID-19 is 75% test or 25% do not test.*"
- Many participants said, "*bear in mind that online tool recommendations have less weight than recommendations from a GP.*" Additional caution is needed in language and terminology use as some patients that felt labelled by tool as high risk, had negative outcomes. Ensuring access to a doctor to debrief with after such tool use is advisable. Retired doctors who are still willing to make a contribution to the society, could play such a role.
- Many participants found the tool by accident; hence it is advisable to advertise tool on social media platforms, billboards, TV, radio and could make it appear at the top of google search. In addition, taking the tool to the people e.g., through road shows could be a useful strategy to reach the old people – if they do not come to the tool, take the tool to the people.
- Many participants compared the tool recommendations with what the Federal Office of Public Health (FOPH) recommended at the time. Having a tool link on FOPH website that stipulates and references the FOPH criteria informing the tool, could increase trust in tool and acceptability. Coordination between FOPH, university hospitals, and other medical professional bodies is recommended to further enhance trust in the tool.
- Many elderly people are willing to embrace telemedicine, but challenges persist. Telephone and voice activated system for the older population or call centers to serve this group, are still needed (taking heed of unreachable and unanswered calls) during this transitional phase.
- Most participants found media confusing – telemedicine could play a better information spreading role, sifting through the noise and offering scientific based recommendations. For many, the media spread fear and misinformed people in many instances.

For peer review only

- The OFTT lacked information on where to test (contact list of testing centres), how to self-care, how to manage symptoms and when to contact a doctor-addressing these shortcomings could improve the utility of OFTTs. Our results underline the importance of not offering a telehealth tool as a stand-alone product, but to integrate it into an overall concept with links to credible reliable sources.
- Systems thinking-refers to the ability to see interconnectedness in a system with a dysfunction in one part affecting other parts and consequently outcomes. Our study revealed the reasons patients did not follow the recommendation to test, as multipronged. Attention has to be paid to supply chain issues, as test shortages affected outcomes. The cost of a test and the fear of a positive result additionally emerged as hindrances to testing. This calls for systems thinking. Noteworthy, is the reaction of GPs who labelled OFTT users who asked for a COVID-19 test as hysteric. This does not only reveal that the pandemic caught everyone by surprise, but also demonstrates the need to involve, collaborate with and win the local health care providers-policy implementers, like GPs and Spitex (home based nursing), to enhance tool utility as well as ensure positive outcomes
- One key informant suggested having patients who had recovered from COVID-19 act as champions to share their illness experience, and motivate the public to take preventive measures and take the disease seriously-an approach that was also effective in HIV prevention and coping strategies.

For peer review only

# CONCLUSION

OFTT use has increased greatly during this pandemic. The effects and utility of such tools however, have not been widely assessed. That makes our study, one of the firsts, in assessing effects and utility of a COVID-19 OFTT. Our study revealed that an OFTT does not only reduce the health system burden but can also serve as an information source, reduce anxiety and fear, reduces potential for onward transmission and facilitate decision making.

For peer review only

# DECLARATIONS

**Acknowledgement:** Emergency telemedicine at University of Bern, Switzerland is supported by an endowed professorship by the Touring Club Switzerland.

**Author contributions:** Study design and idea: J.M., A.M, R.S, T.C.S., M.M, M.E.R, P.J, W.E.H.; Data extraction and preparation: R.S., M.M, A.M; Qualitative interviews: J.M., R.S., A.M.; Statistical analysis: M.M.; Qualitative analysis: JM. Writing of first draft: J.M., A.M, M.M.; Revision of the final draft and final approval: all authors; Supervision: T.C.S., W.E.H.; Project administration: T.C.S., W.E.H.

**Category of article:** original article

**Running title:** Usage of online tools during the COVID-19 pandemic

**Source of funding:** Swiss National Science Foundation (Project ID: 196615)

**Conflict of interest:** This manuscript is partially founded by the Swiss National Science Foundation (Project ID: 196615). The founder has no influence on the content of the manuscript or the decision to publish it.
TCS holds the endowed professorship for emergency telemedicine at University of Bern, Switzerland. The founder has no influence on the research performed, the content of any manuscript or any decision to publish. All other authors have nothing to disclose.

**Data sharing**
Due to the nature of the study (OFTT) participants did not agree for their data to be shared publicly. The data to support findings are available. Please contact corresponding author JM.

For peer review only

# REFERENCES


1. Higgins-Dunn N. Coronavirus cases surpass 25 million as pandemic strains nations around the world. CNBC. Published August 30, 2020. Accessed August 31, 2020. https://www.cnbc.com/2020/08/30/global-coronavirus-cases-surpass-25-million-as-pandemic-strains-nations-around-the-world.html

2. Switzerland Coronavirus: 42,393 Cases and 2,010 Deaths - Worldometer. Accessed September 1, 2020. https://www.worldometers.info/coronavirus/country/switzerland/

3. Germany Coronavirus: 244,792 Cases and 9,371 Deaths - Worldometer. Accessed September 1, 2020. https://www.worldometers.info/coronavirus/country/germany/

4. United Kingdom Coronavirus: 335,873 Cases and 41,501 Deaths - Worldometer. Accessed September 1, 2020. https://www.worldometers.info/coronavirus/country/uk/

5. Munsch N, Martin A, Gruarin S, et al. *A Benchmark of Online COVID-19 Symptom Checkers*. Health Informatics; 2020. doi:10.1101/2020.05.22.20109777

6. Hollander JE, Carr BG. Virtually Perfect? Telemedicine for Covid-19. *N Engl J Med*. 2020;382(18):1679-1681. doi:10.1056/NEJMp2003539

7. Schrager J, Schuler K, Wright D, et al. Development and Usability Testing of a Web-based COVID-19 Self-triage Platform. *West J Emerg Med*. 2020;21(5). doi:10.5811/westjem.2020.7.48217

8. Hamm JM, Greene C, Sweeney M, et al. Telemedicine in the emergency department in the era of COVID-19: front-line experiences from 2 institutions. *J Am Coll Emerg Physicians Open*. n/a(n/a). doi:10.1002/emp2.12204

9. Kellermann AL, Isakov AP, Parker R, Handrigan MT, Foldy S. Web-Based Self-Triage of Influenza-Like Illness During the 2009 H1N1 Influenza Pandemic. *Ann Emerg Med*. 2010;56(3):288-294.e6. doi:10.1016/j.annemergmed.2010.04.005

10. Spaulding AB, Radi D, Macleod H, et al. Design and Implementation of a Statewide Influenza Nurse Triage Line in Response to Pandemic H1N1 Influenza. *Public Health Rep*. 2012;127(5):532-540.

11. Gann B. Giving patients choice and control: health informatics on the patient journey. *Yearb Med Inform*. 2012;7:70-73.

12. Joshi AU,, Lewiss RE. Telehealth in the time of COVID-19. *Emerg Med J*. Published online 2020. doi:10.1136/ emermed-2020-209846

13. Judson TJ, Odisho AY, Neinstein AB, et al. Rapid design and implementation of an integrated patient self-triage and self-scheduling tool for COVID-19. *J Am Med Inform Assoc JAMIA*. 2020;27(6):860-866. doi:10.1093/jamia/ocaa051

14. Amir-Behghadami M, Janati A. Iranian national COVID-19 electronic screening system: experience to share. *Emerg Med J*. 2020;37(7):412-413. doi:10.1136/emermed-2020-209806


For peer review only

15. Meyer AND, Giardina TD, Spitzmueller C, Shahid U, Scott TMT, Singh H. Patient Perspectives on the Usefulness of an Artificial Intelligence-Assisted Symptom Checker: Cross-Sectional Survey Study. *J Med Internet Res*. 2020;22(1):e14679. doi:10.2196/14679

16. Why do many seniors have trouble using technology? No Isolation. Accessed September 8, 2020. https://www.noisolation.com/global/research/why-do-many-seniors-have-trouble-using-technology/

17. Conger K, Griffith E. As Life Moves Online, an Older Generation Faces a Digital Divide. *The New York Times*. https://www.nytimes.com/2020/03/27/technology/virus-older-generation-digital-divide.html. Published March 27, 2020. Accessed September 8, 2020.

18. Digital inclusion for low-skilled and low-literate people: a landscape review - UNESCO Digital Library. Accessed September 8, 2020. https://unesdoc.unesco.org/ark:/48223/pf0000261791

19. Millenson ML, Baldwin JL, Zipperer L, Singh H. Beyond Dr. Google: the evidence on consumer-facing digital tools for diagnosis. *Diagn Berl Ger*. 2018;5(3):95-105. doi:10.1515/dx-2018-0009

20. Morse JM. Determining Sample Size. *Qual Health Res*. 2000;10(1):3-5. doi:10.1177/104973200129118183

21. Chambers D, Cantrell AJ, Johnson M, et al. Digital and online symptom checkers and health assessment/triage services for urgent health problems: systematic review. *BMJ Open*. 2019;9(8). doi:10.1136/bmjopen-2018-027743

22. Chambers D, Cantrell AJ, Johnson M, et al. Digital and online symptom checkers and health assessment/triage services for urgent health problems: systematic review. *BMJ Open*. 2019;9(8). doi:10.1136/bmjopen-2018-027743

23. CoronaCheck for healthcare professionals | CoronaCheck Unisanté. Accessed December 1, 2020. https://coronavirus.unisante.ch/en/professionnels

24. The not-so-straightforward measurement of socioeconomic status. The Dunn Lab. Accessed September 18, 2020. http://www.thedunnlab.com/updates/measuringses

25. Nelson CA, Pérez-Chada LM, Creadore A, et al. Patient Perspectives on the Use of Artificial Intelligence for Skin Cancer Screening: A Qualitative Study. *JAMA Dermatol*. 2020;156(5):501-512. doi:10.1001/jamadermatol.2019.5014

26. What questions are priority areas for primary care during the COVID-19 pandemic? A rapid question generation and prioritisation exercise. CEBM. Accessed July 14, 2020. https://www.cebm.net/covid-19/what-questions-are-priority-areas-for-primary-care-during-the-covid-19-pandemic-a-rapid-question-generation-and-prioritisation-exercise/

27. Advantages and Disadvantages of using Symptoms Checker ⋆ The Stuff of Success. Accessed October 19, 2020. https://thestuffofsuccess.com/2018/04/08/advantages-and-disadvantages-of-using-symptoms-checker/

28. Symptom Checkers and Self-Triage Tools in the Era of COVID-19: What's the Evidence for their Use? NODE.Health. Published April 17, 2020. Accessed September 18, 2020.


For peer review only

https://nodehealth.org/2020/04/17/symptom-checkers-and-self-triage-tools-in-the-era-of-covid-19-whats-the-evidence-for-their-use/

29. How Online Searches Fuel Health-Related Anxiety. ICANotes. Published May 7, 2019. Accessed October 19, 2020. https://www.icanotes.com/2019/05/07/how-online-searches-fuel-health-related-anxiety/

30. Van Riel N, Auwerx K, Debbaut P, Van Hees S, Schoenmakers B. The effect of Dr Google on doctor–patient encounters in primary care: a quantitative, observational, cross-sectional study. *BJGP Open*. 1(2). doi:10.3399/bjgpopen17X100833

31. The "Untold" Side of COVID-19: Social Stigma and Its Consequences in India - Prama Bhattacharya, Debanjan Banerjee, TS Sathyanarayana Rao, 2020. Accessed September 18, 2020. https://journals.sagepub.com/doi/full/10.1177/0253717620935578

32. How Fear Inhibits HIV Testing and Treatment in African Men. Contagion Live. Accessed October 19, 2020. https://www.contagionlive.com/view/how-fear-inhibits-hiv-testing-and-treatment-in-african-men

33. Desmon S. Study: Fear Keeps Men from Getting Tested for HIV. Johns Hopkins Center for Communication Programs. Published October 28, 2019. Accessed October 19, 2020. https://ccp.jhu.edu/2019/10/28/study-fear-keeps-men-from-hiv-testing/

34. More Than Half of COVID-19 Survivors in Study Reported Psychiatric Disorders. EcoWatch. Published August 4, 2020. Accessed September 18, 2020. https://www.ecowatch.com/covid-19-psychiatric-disorders-2646878306.html

35. Varatharaj A, Thomas N, Ellul MA, et al. Neurological and neuropsychiatric complications of COVID-19 in 153 patients: a UK-wide surveillance study. *Lancet Psychiatry*. 2020;7(10):875-882. doi:10.1016/S2215-0366(20)30287-X

36. Birkhäuer J, Gaab J, Kossowsky J, et al. Trust in the health care professional and health outcome: A meta-analysis. *PLoS ONE*. 2017;12(2). doi:10.1371/journal.pone.0170988

37. Brennan N, Barnes R, Calnan M, Corrigan O, Dieppe P, Entwistle V. Trust in the health-care provider–patient relationship: a systematic mapping review of the evidence base. *Int J Qual Health Care*. 2013;25(6):682-688. doi:10.1093/intqhc/mzt063

38. Anhang Price R, Fagbuyi D, Harris R, et al. Feasibility of Web-Based Self-Triage by Parents of Children With Influenza-Like Illness: A Cautionary Tale. *JAMA Pediatr*. 2013;167(2):112. doi:10.1001/jamapediatrics.2013.1573

39. EBSCO HEALTH. Enabling Patient Engagement with a Symptom Checker. Published online 2020:16.

40. Schmieding ML, Mörgeli R, Schmieding MAL, Feufel MA, Balzer F. Benchmarking Triage Capability of Symptom Checkers Against That of Medical Laypersons: Survey Study. *J Med Internet Res*. 2021;23(3):e24475. doi:10.2196/24475

41. Ashwood JS, Mehrotra A, Cowling D, Uscher-Pines L. Direct-To-Consumer Telehealth May Increase Access To Care But Does Not Decrease Spending. *Health Aff (Millwood)*. 2017;36(3):485-491. doi:10.1377/hlthaff.2016.1130

For peer review only

For peer review only - http://bmjopen.bmj.com/site/about/guidelines.xhtml

# LIST OF FIGURES

**Figure 1: Mixed-Methods Sequential Explanatory Study Design**

**Fig 2: OFTT triage**

For peer review only

**Figure 1: Mixed-Methods Sequential Explanatory Study Design.**

| Phase | Procedure | Product |
|---|---|---|
| **Collection of clinical data** | OFTT | Data about usage of the tool<br>Contact data for later study phases |
| **Quantitative data collection** | Questionnaires | Numeric data |
| **Quantitative data analysis** | Statistical analysis | Descriptive statistics |
| **Connecting quantitative and qualitative data** | Purposefully selecting patients for interviews | Interview guide |
| **Qualitative data collection** | Individual in-depth interviews | Interview transcripts |
| **Qualitative data analysis** | Coding and thematic analysis | Codes and themes |
| **Integration of qualitative and quantitative results** | Interpretation and explanation of quantitative results with the aid of and qualitative findings | Implications for OFTT development |

For peer review only

**Fig 2 OFTT triage**

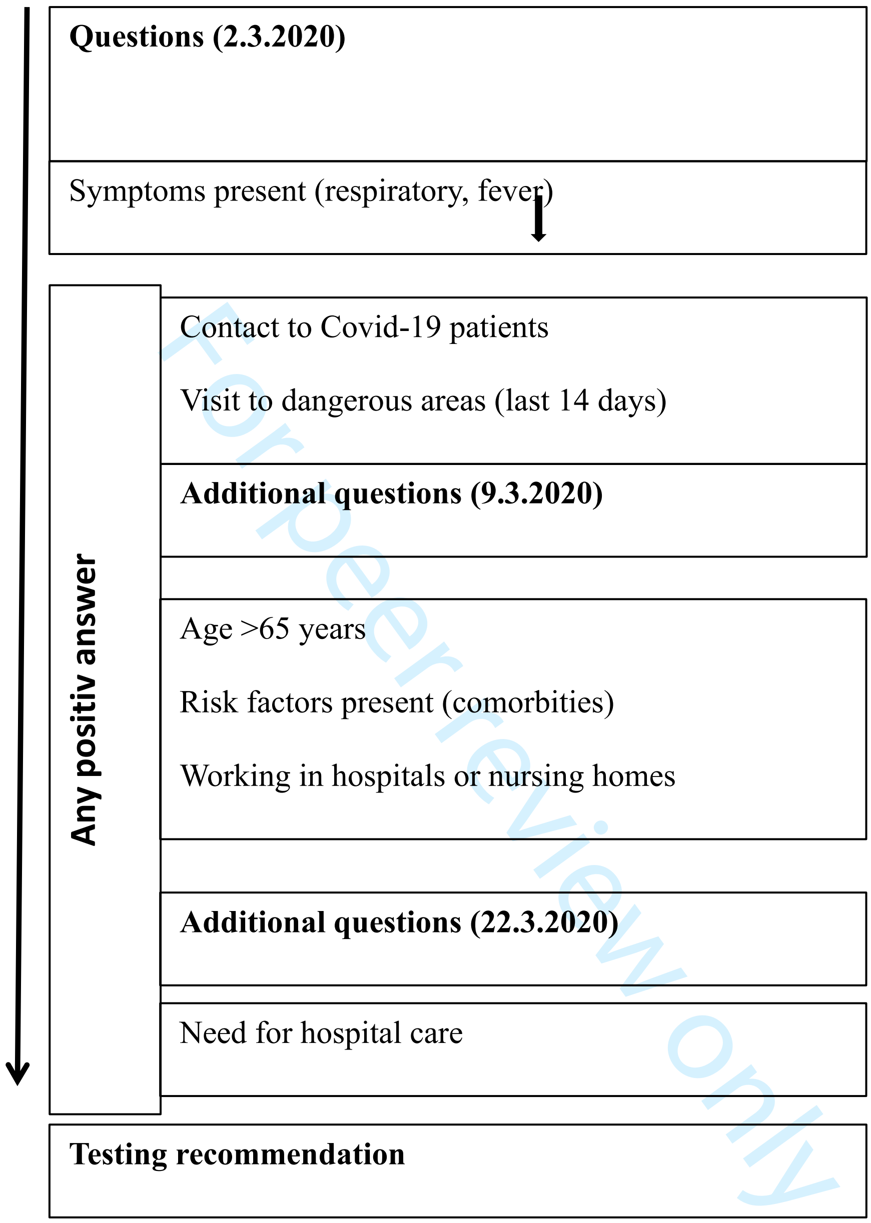


For peer review only

# Supporting information OFTT Questionnaire and results

## Supporting information 1. Usage of online tools during the COVID-19 pandemic: email survey

| | | | |
|---|---|---|---|
| v1 | **How did you get to this Online - Tool?** | | |
| | 1 - My family doctor advised me to use this tool. | 9 | 5.1% |
| | 2 - I found the information on the Internet. | 113 | 64.2% |
| | 3 - The website was recommended to me by family/friends. | 17 | 9.7% |
| | 4 - Via a telephone hotline. | 1 | 0.6% |
| | 5 - Other (Free text) | 35 | 19.9% |
| v2 | **Did you find the information that you needed?** | | |
| | 1 - Yes | 154 | 87.5% |
| | 2 - No, because the information was not comprehensive. | 17 | 9.7% |
| | 3 - No, because the information was not clear. | 5 | 2.8% |
| v3 | **What information did you search for? I wanted....** | | |
| | 1 - ... more information on COVID-19 symptoms | 97 | 55.1% |
| | 2 - ... more information on how to cope with symptoms | 4 | 2.3% |
| | 3 - ... to know when to consult a doctor | 36 | 20.5% |
| | 4 - ... to know more on testing criteria | 32 | 18.2% |
| | 5 - ... to know where to test | 7 | 4.0% |
| | 6 - Other | - | - |
| v4 | **Did the online tool recommend you to test for COVID-19?** | | |
| | 1 - Yes | 56 | 31.8% |
| | 2 - No | 120 | 68.2% |
| v5 | **Did you stick to the recommendations?** | | |
| | 1 - Yes | 149 | 84.7% |
| | 2 - No | 27 | 15.3% |
| v6 | **If you followed the Online - Tool recommendations, what made you do so?** | | |
| | 1 - I trust the website as a reliable information source. | 60 | 34.1% |
| | 2 - I compared the recommendations with recommendations from the media and took a decision. | 20 | 11.4% |
| | 3 - I compared the recommendations with those from FOPH (BAG) and took a decision. | 53 | 30.1% |
| | 4 - I sought advice from a person I trusted. | 7 | 4.0% |
| | 5 - Other, please specify: Free text | 9 | 5.1% |
| v7 | **In case you did not follow the recommendations, why did you not** | | |
| | 1 - I did not trust the website as a reliable source of information. | 1 | 0.6% |
| | 2 - The recommendations from the website differed from the media recommendations. | 2 | 1.1% |
| | 3 - I feared for my life and needed to consult a GP in person. | 6 | 3.4% |
| | 4 - Other, please specify: Free text | 18 | 10.2% |
| v8 | **Were your fears and anxieties allayed after visiting the website?** | | |
| | 1 - Yes, the information from the website reassured me. | 73 | 41.5% |
| | 2 - No, the information from the website did not reassure me. | 13 | 7.4% |

For peer review only - http://bmjopen.bmj.com/site/about/guidelines.xhtml

| | | | |
|---|---|---|---|
| | 3 - No, the information from the website increased my fears and anxieties. | 6 | 3.4% |
| | 4 - I was not worried. | 84 | 47.7% |
| v9 | **How did you cope with your fears? What helped you cope?** | | |
| | 1 - Free text | - | - |
| v10 | **In case you went to the GP, did you call ahead of time to notify them of your visit?** | | |
| | 1 - Yes | 115 | 65.3% |
| | 2 - No | 61 | 34.7% |
| v11 | **Did you get tested for Coronavirus (SARS-CoV-2 Swab)?** | | |
| | 1 - Yes | 48 | 27.3% |
| | 2 - No | 128 | 72.7% |
| v12 | **What was the result?** | | |
| | 1 - Positive | 3 | 1.7% |
| | 2 - Negative | 45 | 25.6% |
| v13 | **How did the media influence your decision making? The** | | |
| | 1 - ... helpful | 81 | 46.0% |
| | 2 - ... confusing | 47 | 26.7% |
| | 3 - I do not rely on the media as an information source. | 25 | 14.2% |
| | 4 - Free text | 23 | 13.1% |
| v14 | **How old are you?** | Mean 50.5 (SD 15), range 18-82 | |
| v15 | **What is your sex?** | | |
| | 1 - Female | 101 | 57.4% |
| | 2 - Male | 75 | 42.6% |
| | 3 - Other | 0 | 0.0% |
| v16 | **What is your nationality?** | | |
| | 0 - Missing | 0 | 0.0% |
| | 1 - Swiss | 147 | 83.5% |
| | 2 - German | 13 | 7.4% |
| | 3 - French | 1 | 0.6% |
| | 4 - Italian | 3 | 1.7% |
| | 5 - Liechtenstein | 0 | 0.0% |
| | 6 - Greater Europe | 4 | 2.3% |
| | 7 - Free text | 7 | 4.0% |
| v17 | **In which province do you live?** | | |
| | 1 - Bern | 108 | 61.4% |
| | 2 - Zürich | 12 | 6.8% |
| | 3 - Luzern | 10 | 5.7% |
| | 4 - Uri | 0 | 0.0% |
| | 5 - Schwyz | 1 | 0.6% |
| | 6 - Obwalden | 0 | 0.0% |
| | 7 - Nidwalden | 0 | 0.0% |
| | 8 - Glarus | 0 | 0.0% |
| | 9 - Zug | 2 | 1.1% |
| | 10 - Fribourg | 7 | 4.0% |
| | 11 - Solothurn | 3 | 1.7% |
| | 12 - Basel-Stadt | 2 | 1.1% |
| | 13 - Basel-Landschaft | 1 | 0.6% |
| | 14 - Schaffhausen | 0 | 0.0% |

For peer review only

| | | | |
|---|---|---|---|
| | 15 - Appenzell Ausserrhoden | 2 | 1.1% |
| | 16 - Appenzell Innerrhoden | 0 | 0.0% |
| | 17 - St. Gallen | 2 | 1.1% |
| | 18 - Graubünden | 3 | 1.7% |
| | 19 - Aargau | 9 | 5.1% |
| | 20 - Thurgau | 1 | 0.6% |
| | 21 - Ticino | 2 | 1.1% |
| | 22 - Vaud | 7 | 4.0% |
| | 23 - Valais | 0 | 0.0% |
| | 24 - Neuchâtel | 1 | 0.6% |
| | 25 - Geneva | 0 | 0.0% |
| | 26 - Jura | 0 | 0.0% |
| | 27 - I do not live in Switzerland | 3 | 1.7% |
| v18 | **What is your highest level of education?** | | |
| | 0 - Missing | 6 | 3.4% |
| | 1 - Tertiary education (university degree, college of education) | 120 | 68.2% |
| | 2 - Upper secondary education (High School Graduation, FMS, EZF, EBA) | 27 | 15.3% |
| | 3 - Lower secondary education/ obligatory schooling completed | 23 | 13.1% |
| | 4 - No formal education | | |
| v19 | **Are you currently...** | | |
| | 0 - Missing | 33 | 18.8% |
| | 1 - Employed | 106 | 60.2% |
| | 2 - Self employed | 24 | 13.6% |
| | 3 - Unemployed already before the current pandemic | 3 | 1.7% |
| | 4 - I lost my job during the COVID-19 period | 1 | 0.6% |
| | 5 - Studying or in an apprenticeship | 9 | 5.1% |
| v20 | **How much approximately do you earn per month? (net income in December 2019 including 1/12 of the 13th month salary.)** | | |
| | 0 - Missing | 29 | 16.5% |
| | 1 - Less that 4'000 CHF | 26 | 14.8% |
| | 2 - Between 4'001 und 6'000 CHF | 42 | 23.9% |
| | 3 - Above 6'001 CHF | 79 | 44.9% |
| v21 | **What type of health insurance do you have?** | | |
| | 1 - General | 68 | 38.6% |
| | 2 - Telemedicine - Modell | 12 | 6.8% |
| | 3 - GP - Modell | 83 | 47.2% |
| | 4 - Another alternative model | 8 | 4.5% |
| | 5 - No insurance | 5 | 2.8% |
| v22 | **In a second stage, we will interview individual participants of** | | |
| | 1 - Yes, I consent to be contacted. | 78 | 44.3% |
| | 2 - No, please, no more interviews. | 98 | 55.7% |
| | 3 - Free text | - | - |

For peer review only

## Interview Guide: Coronatest.ch -Patients v 2

# Rapport

0Describe yourself (prompts; nationality, occupation, living arrangements, employment status)

# Accessibility

0How did you get to coronatest.ch website? (referred by …, online search). What is your understanding of an OFTT?

0Did you consult BAG- was the information supplied by BAG understandable to you? In what way was it useful.

0Was the online digital tool easy for you to access? explain why or why not (easy to find on homepage, length of tool, clear and easy to follow instructions, language, sequence, when did you use the tool Monday, Tuesday, weekend or during the week and why)

0What information or components would have helped you better -what do you suggest needs to be done to make such an online too more accessible.

# Utility as a reliable information source and decision making

0When you consulted the online digital tool; did you follow the recommended advice? Prompt (why and how socioeconomic status could have influenced the process)

0What made you follow the advice and recommendations? Prompt confirmation from friends, generally, the media influence your decision-making process?

0What made you disregard the advice and recommendations? (prompt for severity of symptoms, change of condition)

0We have noticed that people that earn below 4000 tend not to follow recommendations, why do you think it`s like this, what can be done?

# Utility in allaying fear and anxiety

0Describe how you felt after consulting the online digital tool with regards to feeling anxious and or confident that all was going to be well? (Did you feel reassured after visiting corontest.ch)

0If your fears were not allayed, how did you deal with your fears? prompt on what increased confidence, what allayed your anxiety)

For peer review only

# Illness and testing Experience

0Did you test for COVID-19, what test and experience? Did you experience COVID-19 symptoms? Explain

0Speaking of self-isolation. Please explain in more detail how you experienced this what worked and what did not work e.g. the need to go shopping, not going for a run or walk protecting others vs own needs, putting others first, dilemmas, challenges

0The road to recovery has been described by many as very cumbersome and long-what was your experience? Any psychiatric or other residual effects experienced-explain.

0What personal life lessons did you learn during this pandemic you would like to share and what personal changes do you foresee in future

0What health and health system related observations did you make and what changes do you fore see in future?

0What, socio-economic changes have you observed and do you foresee in the future as a result of COVID-19

# Utility in preventing cross infection

0If you consulted a GP; did you call ahead of time?

0How did your GP/ health care provider react when you told him or her you suspected that you had COVID-19?

# Recommendations

0In a future pandemic, what would you do?

0Is there any additional information you wish a site like corontest provides but was missing during COVID-19? What information did you search for but did not find?

0Are there other strategies (to allay fear, anxiety) you deem effective alone or in conjunction with online digital tools when faced with epidemics such as COVID-19 to make it accessible to the older generation?

0How can online tools like corontast be adapted to facilitate your decision making processes

For peer review only

STROBE Statement—checklist of items that should be included in reports of observational studies

| | Item No | Recommendation | Page No |
|---|---|---|---|
| **Title and abstract** | 1 | (*a*) Indicate the study's design with a commonly used term in the title or the abstract | 1 |
| | | (*b*) Provide in the abstract an informative and balanced summary of what was done and what was found | 3 |
| **Introduction** | | | |
| Background/rationale | 2 | Explain the scientific background and rationale for the investigation being reported | 5 |
| Objectives | 3 | State specific objectives, including any prespecified hypotheses | 5 |
| **Methods** | | | |
| Study design | 4 | Present key elements of study design early in the paper | 6 |
| Setting | 5 | Describe the setting, locations, and relevant dates, including periods of recruitment, exposure, follow-up, and data collection | 6 |
| Participants | 6 | (*a*) *Cohort study*—Give the eligibility criteria, and the sources and methods of selection of participants. Describe methods of follow-up<br>*Case-control study*—Give the eligibility criteria, and the sources and methods of case ascertainment and control selection. Give the rationale for the choice of cases and controls<br>*Cross-sectional study*—Give the eligibility criteria, and the sources and methods of selection of participants | 7 |
| | | (*b*) *Cohort study*—For matched studies, give matching criteria and number of exposed and unexposed<br>*Case-control study*—For matched studies, give matching criteria and the number of controls per case | |
| Variables | 7 | Clearly define all outcomes, exposures, predictors, potential confounders, and effect modifiers. Give diagnostic criteria, if applicable | 7 |
| Data sources/ measurement | 8* | For each variable of interest, give sources of data and details of methods of assessment (measurement). Describe comparability of assessment methods if there is more than one group | *8-9* |
| Bias | 9 | Describe any efforts to address potential sources of bias | 9 |
| Study size | 10 | Explain how the study size was arrived at | 8 |
| Quantitative variables | 11 | Explain how quantitative variables were handled in the analyses. If applicable, describe which groupings were chosen and why | 10 |
| Statistical methods | 12 | (*a*) Describe all statistical methods, including those used to control for confounding | 10 |
| | | (*b*) Describe any methods used to examine subgroups and interactions | 10 |
| | | (*c*) Explain how missing data were addressed | 10.11.12.13 |
| | | (*d*) *Cohort study*—If applicable, explain how loss to follow-up was addressed<br>*Case-control study*—If applicable, explain how matching of cases and controls was addressed<br>*Cross-sectional study*—If applicable, describe analytical methods taking account of sampling strategy | |


For peer review only - http://bmjopen.bmj.com/site/about/guidelines.xhtml
For peer review only

| | |
|---|---|
| (*e*) Describe any sensitivity analyses | 12 |

Continued on next page

For peer review only


For peer review only - http://bmjopen.bmj.com/site/about/guidelines.xhtml

| | | | |
|---|---|---|---|
| **Results** | | | |
| Participants | 13* | (a) Report numbers of individuals at each stage of study—eg numbers potentially eligible, examined for eligibility, confirmed eligible, included in the study, completing follow-up, and analysed | 11 |
| | | (b) Give reasons for non-participation at each stage | |
| | | (c) Consider use of a flow diagram | |
| Descriptive data | 14* | (a) Give characteristics of study participants (eg demographic, clinical, social) and information on exposures and potential confounders | 11 |
| | | (b) Indicate number of participants with missing data for each variable of interest | 11 |
| | | (c) *Cohort study*—Summarise follow-up time (eg, average and total amount) | |
| Outcome data | 15* | *Cohort study*—Report numbers of outcome events or summary measures over time | |
| | | *Case-control study*—Report numbers in each exposure category, or summary measures of exposure | |
| | | *Cross-sectional study*—Report numbers of outcome events or summary measures | |
| Main results | 16 | (*a*) Give unadjusted estimates and, if applicable, confounder-adjusted estimates and their precision (eg, 95% confidence interval). Make clear which confounders were adjusted for and why they were included | 12.13.14 |
| | | (*b*) Report category boundaries when continuous variables were categorized | |
| | | (*c*) If relevant, consider translating estimates of relative risk into absolute risk for a meaningful time period | |
| Other analyses | 17 | Report other analyses done—eg analyses of subgroups and interactions, and sensitivity analyses | |
| **Discussion** | | | |
| Key results | 18 | Summarise key results with reference to study objectives | 18 |
| Limitations | 19 | Discuss limitations of the study, taking into account sources of potential bias or imprecision. Discuss both direction and magnitude of any potential bias | 21 |
| Interpretation | 20 | Give a cautious overall interpretation of results considering objectives, limitations, multiplicity of analyses, results from similar studies, and other relevant evidence | 20 |
| Generalisability | 21 | Discuss the generalisability (external validity) of the study results | 21 |
| **Other information** | | | |
| Funding | 22 | Give the source of funding and the role of the funders for the present study and, if applicable, for the original study on which the present article is based | 24 |

*Give information separately for cases and controls in case-control studies and, if applicable, for exposed and unexposed groups in cohort and cross-sectional studies.

**Note:** An Explanation and Elaboration article discusses each checklist item and gives methodological background and published examples of transparent reporting. The STROBE checklist is best used in conjunction with this article (freely available on the Web sites of PLoS Medicine at http://www.plosmedicine.org/, Annals of Internal Medicine at http://www.annals.org/, and Epidemiology at http://www.epidem.com/). Information on the STROBE Initiative is available at www.strobe-statement.org.


For peer review only - http://bmjopen.bmj.com/site/about/guidelines.xhtml

BMJ Open

# Effects and utility of an online forward triage tool during the SARS-CoV-2 pandemic: a mixed method study and patient perspectives, Switzerland

| | |
|---|---|
| Journal: | *BMJ Open* |
| Manuscript ID | bmjopen-2021-059765.R2 |
| Article Type: | Original research |
| Date Submitted by the Author: | 25-May-2022 |
| Complete List of Authors: | Michel, Janet; Inselspital Universitätsspital Bern, Department of Emergency Medicine<br>Mettler, Annette; University of Bern, Department of Emergency Medicine<br>Stuber, Raphael; University of Bern, Department of Emergency Medicine<br>Müller, Martin; University of Bern, Department of Emergency Medicine<br>Ricklin, Meret; University of Bern, Department of Emergency Medicine<br>Jent, Philipp; University of Bern, Department of Infectious Diseases<br>Hautz, Wolf; University of Bern, Department of Emergency Medicine; University of Oslo<br>Sauter, Thomas; University of Bern, Department of Emergency Medicine |
| <b>Primary Subject Heading</b>: | Emergency medicine |
| Secondary Subject Heading: | Health policy, Qualitative research, Public health, Emergency medicine |
| Keywords: | COVID-19, Public health < INFECTIOUS DISEASES, QUALITATIVE RESEARCH, Telemedicine < BIOTECHNOLOGY & BIOINFORMATICS, EPIDEMIOLOGY |
| | |

SCHOLARONE™
Manuscripts

*I, the Submitting Author has the right to grant and does grant on behalf of all authors of the Work (as defined in the below author licence), an exclusive licence and/or a non-exclusive licence for contributions from authors who are: i) UK Crown employees; ii) where BMJ has agreed a CC-BY licence shall apply, and/or iii) in accordance with the terms applicable for US Federal Government officers or employees acting as part of their official duties; on a worldwide, perpetual, irrevocable, royalty-free basis to BMJ Publishing Group Ltd ("BMJ") its licensees and where the relevant Journal is co-owned by BMJ to the co-owners of the Journal, to publish the Work in this journal and any other BMJ products and to exploit all rights, as set out in our licence.*

*The Submitting Author accepts and understands that any supply made under these terms is made by BMJ to the Submitting Author unless you are acting as an employee on behalf of your employer or a postgraduate student of an affiliated institution which is paying any applicable article publishing charge ("APC") for Open Access articles. Where the Submitting Author wishes to make the Work available on an Open Access basis (and intends to pay the relevant APC), the terms of reuse of such Open Access shall be governed by a Creative Commons licence – details of these licences and which Creative Commons licence will apply to this Work are set out in our licence referred to above.*

*Other than as permitted in any relevant BMJ Author's Self Archiving Policies, I confirm this Work has not been accepted for publication elsewhere, is not being considered for publication elsewhere and does not duplicate material already published. I confirm all authors consent to publication of this Work and authorise the granting of this licence.*

For peer review only

# Effects and utility of an online forward triage tool during the SARS-CoV-2 pandemic: a mixed method study and patient perspectives, Switzerland

Janet Michel[1]*, Annette Mettler[1]*, Raphael Stuber[1], Martin Müller[1], Meret E. Ricklin[1], Philipp Jent[3], Wolf E. Hautz[1,2], Thomas C. Sauter[1]

**Affiliations:**
[1] Department of Emergency Medicine, Inselspital, University Hospital, University of Bern, Bern, Switzerland
[2] Centre for Educational Measurement, University of Oslo, Oslo, Norway
[3] Department of Infectious Diseases, Inselspital, University Hospital, University of Bern, Bern, Switzerland
* Co-first authorship

**Author Details**

**Corresponding Author:**
Janet Michel RN, BA Cur, MPH, PhD
Department of Emergency Medicine
Inselspital, University Hospital, University of Bern.
3010 Bern, Switzerland
E-Mail: janetmichel71@gmail.com
Phone: +41 326322111

**Author 2**
Annette Mettler
Department of Emergency Medicine
Inselspital, University Hospital, University of Bern.
3010 Bern, Switzerland
E-Mail: annette.mettler@insel.ch

**Author 3**
Raphael Stuber
Department of Emergency Medicine
Inselspital, University Hospital, University of Bern.
3010 Bern, Switzerland
E-Mail: raphael.stuber@students.unibe.ch

**Author 4**
Martin Müller
Department of Emergency Medicine
Inselspital, University Hospital, University of Bern.
3010 Bern, Switzerland
E-Mail: martin.mueller2@insel.ch

For peer review only

**Author 5**
Meret E. Ricklin
Department of Emergency Medicine
Inselspital, University Hospital, University of Bern.
3010 Bern, Switzerland
E-Mail: meret.ricklin@insel.ch

**Author 6**
Philipp Jent
Department of Infectious Diseases
Inselspital, University Hospital, University of Bern.
3010 Bern, Switzerland
E-Mail: philipp.jent@insel.ch

**Author 7**
Wolf E. Hautz
Department of Emergency Medicine
Inselspital, University Hospital, University of Bern.
3010 Bern, Switzerland
E-Mail: wolf.hautz@insel.ch

**Author 8**
Thomas C. Sauter
Department of Emergency Medicine
Inselspital, University Hospital, University of Bern.
3010 Bern, Switzerland
E-Mail: thomas.sauter@insel.ch

**Word count 5 600**

**Key words**

online forward triage tool (OFTT), effects, utility, SARS-CoV-2, COVID-19, pandemic

For peer review only

# ABSTRACT

**Objective:** To assess the effects (quantitatively) and the utility (qualitatively) of a COVID-19 online forward triage tool (OFTT) in a pandemic context.
**Design**: A mixed-method sequential explanatory study was employed. Quantitative data of all OFTT users, between March 2nd, 2020 and May 12th, 2020 were collected. Secondly, qualitative data was collected through key informant interviews (n=19) to explain the quantitative findings, explore tool utility, user experience and elicit recommendations.
**Setting**: The working group e-emergency medicine at the emergency department (ED), developed an OFTT which was made available online
**Participants**: Participants included all users above the age of 18 that used the OFTT between March 2nd, 2020 and May 12th, 2020
**Intervention**: An OFTT that displayed the current test recommendations of the Federal Office of Public Health (FOPH) on whether someone needed testing for COVID-19 or not. No diagnosis was provided
**Results:** In the study period, 6,272 users consulted our OFTT; 40.2% (1626/4049) would have contacted a healthcare provider had the tool not existed. 560 participants consented to a follow-up survey and provided a valid e-mail address. 31.4% (176/560) participants returned a complete follow-up questionnaire. 84.7% (149/176) followed the recommendations given. 41.5% (73/176) reported that their fear was allayed after using tool. Qualitatively, seven overarching themes emerged namely i) accessibility of tool, ii) user-friendliness of tool, iii) utility of tool as an information source, iv) utility of tool in allaying fear and anxiety, v) utility of tool in medical decision making vi) utility of tool in reducing the potential for onward transmissions and vii) utility of tool in reducing health system burden.
**Conclusion:** Our findings demonstrated that a COVID-19 OFTT does not only reduce the health system burden, but can also serve as an information source, reduce anxiety and fear, reduce potential for cross infections and facilitate medical decision making.

**Word count 299**

**Strengths and limitations**

- The mixed method sequential explanatory design facilitated a holistic understanding of OFTTs.
- Perspectives of those that do not use online tools are missing.
- The long duration between tool use and the qualitative interviews could have introduced a certain degree of recall bias.
- Self-report bias cannot be ruled out.

For peer review only

For peer review only - http://bmjopen.bmj.com/site/about/guidelines.xhtml

# INTRODUCTION

The number of COVID-19 cases across the globe has surpassed 25 million and incident rates are again on the rise as many European countries experience subsequent waves.[1–4] Many people are seeking reliable information, recommendations on testing and management of COVID-19 as well as reassurance, adding to the health system burden. Online forward triage tools (OFTT) are being widely used during this COVID-19 pandemic context[5–8] as misinformation and worry in the population abound. There is evidence from an earlier 2009 H1N1 influenza pandemic, that online tools are effective and practical in reducing the health system burden.[9,10] There is also emerging evidence of this nature from the COVID-19 context. [6,11–14] For example, OFTTs help reduce exposure of worried but uninfected and infected persons, through avoidance of hospitals and doctors' offices – enabling patients to access recommendations of what to do, from the comfort of their own homes.[10,11]

Using OFTTs is relatively easy to the computer literate. People respond to questions and upon completion, recommendations are given, e.g., isolate, test, do not test etc. Existing evidence on the effects and utility of OFTTs differ with possible implications on the quality of the symptom assessment [5]. According to literature, the reasons patients use symptom checkers or OFTTs are i) to understand the causes of their symptoms (76%), ii) to determine whether or not to seek care (33%), and iii) where to seek care (21%).[15] There is also evidence that patients that have previously experienced a diagnostic error are more likely to use OFTT to search for where to seek care [15] than those that have not.

**Challenges with OFTT use and research gap**

In the European Union, 87% of people aged 75 years and above have never been online according to a recent survey.[16]That means the elderly, may be less inclined to use online tools if not computer literate. This in turn shuts the elderly out from society, increasing isolation and loneliness, not to mention the missed health benefits [10]. The digital divide is real [17]. How can digital tools be designed to be more inclusive?[18] Information on factors influencing the use of OFTTs is scant and the validation of COVID-19 OFTTs like other OFTTs, seems neglected. [15,19] That makes the quality assessment of these tools paramount [5] as evidence on effects and utility of OFTTs is limited.

**The aim of this study**

This study aimed at assessing the effects (quantitatively) and the utility (qualitatively) of a COVID-19 OFTT during a pandemic context in Switzerland, exploring patient perspectives and derive recommendations for tool improvement. We hypothesized that an OFTT adequately reduces patient visits to the health care system and consequently reduces the health system burden. We further explored qualitatively, for emergent themes, capturing the tool utility to this population.

For peer review only

# Methods

## Study design and participants

We employed a mixed-method sequential explanatory design to study the utility of the OFTT and the effects of using such a tool. The rationale for mixing both kinds of data within one study is that neither qualitative nor quantitative methods, are sufficient by themselves, to capture details of a phenomenon. In combination, they complement each other, taking advantage of the strengths of each. As in sequential explanatory designs, quantitative data collection was done first, as a major component of our study to inform qualitative interviews, see Figure 1.

**About here Figure 1: Mixed-Methods Sequential Explanatory Study Design.**

## Online forward triage tool description and setting

The working group e-emergency medicine at the emergency department (ED), Inselspital University Hospital Bern, together with the Department of Infectious Diseases, Inselspital University Hospital Bern, developed an online forward triage tool (OFTT) which was made available online (*coronatest.ch*). To the best of our knowledge, this was one of the first COVID-19 OFFTs set up in the German speaking part of Switzerland. In a skip-logic, the OFTT displayed the current test recommendations of the Federal Office of Public Health (FOPH) on whether someone needed testing for COVID-19 or not. No diagnosis was provided by the OFTT.

The questions and the content of the OFTT represented the official FOPH recommendations at the time. Thus, the OFTT was comparable in content to other OFTTs in Switzerland, which were based on the FOPH guidelines within that time period. One additional non-mandatory question, which did not affect the result, was integrated in our OFTT from the 11$^{th}$ March 2020, namely the question "What would you do if this online test did not exist?".

There were two possible outcomes of the OFTT: "According to the criteria of the Federal Office of Public Health (BAG), one meets or does not meet the criteria for a test for an infection with the coronavirus, COVID-19". The results page was linked to the FOPH's official behavioural recommendations and recommendations for the testing process. The average time to complete the assessment was 75sec.

## OFTT triage

Details on the structure of the OFTT as well as screen shot are published in a separate quantitative paper[20]. The Federal office of public health (FOPH) national COVID-19 Swiss testing criteria were transferred into a digital decision tree and adjusted promptly after the criteria were adapted by the FOPH. During the first phase of the pandemic, the recommendations for testing or not testing were mainly based on contact with an infected person or a visit to a risk area and were then changed during the course of the pandemic to a testing regime based on risk groups (healthcare professionals, patients>65 years and patients with pre-existing conditions). With the general availability of the tests, the test recommendations were extended to all symptomatic patients and our OFTT became obsolete. Unlike other triage techniques performed on emergency patients, the aim of the OFTT was not to make a COVID-19 diagnosis, assess the risk of severe COVID-19 progression or recommend treatment. See Fig 2 below.

**About here Fig 2: OFTT triage**

## Quantitative data

### Research participants and data collection

Participants included all users above the age of 18 that used the OFTT between March 2nd, 2020 and May 12th, 2020. In this timeframe, the recommendations on COVID-19 frequently changed in Switzerland and there was an initial lack of testing reagents and capacity as well as the risk of overburdening the healthcare system. During the first few weeks of the pandemic, the Federal Office of Public Health (FOPH) recommended testing only for symptomatic patients after travel to high-risk countries (e.g., Italy and China) or symptomatic contacts of coronavirus patients. In weeks that followed (as from the 20th March 2020), the strategy changed to testing of high-risk groups (older than 65 years, pre-existing conditions, and healthcare workers). The countries and risk groups were regularly adjusted according to the spread of the virus and the findings about risk groups but also the availability of testing capacity.
Due to the rapid spread of the virus in Switzerland, and broadly available testing capacities, a universal test recommendation was made by the Federal Office of Public Health (FOPH)- on April 27th, 2020. All symptomatic individuals were eligible to test. With this recommendation, our OFTT provided less benefit to the user and was finally removed on May 12th 2020 from the website paving the way to a second generation OFTT.

To minimise the barrier to the use of the OFTT and for legal data protection reasons, no personal data was collected within the OFTT. Further data on the users of the OFTT was collected in a second step, from participants who gave their explicit consent and provided their email addresses to be contacted. This also made it possible to investigate the adherence to recommendations and the test results. A non-mandatory additional question was built into the OFFT from 11th March 2020.
A pretested online questionnaire (see supplemental file 1) was used to assess the

i) utilization of the OFTT, including way of referral to the tool, reasons for use and information searched,
ii) additional factors, including influence of the media and influence of the OFTT on fear and anxiety.

The database used is compliant with Swiss laws on the collection of personal health related information. The follow-up questionnaire is available as supplemental file 1. Due to ethical reasons, we included the option "not want to answer" as a choice in the questionnaire for the socio-demographic data, in case the respondent did not want to give a statement on this specific sensitive topic.
The qualitative interviews were conducted with purposefully selected key informants who gave their consent during the survey (see below).

### Data analysis

Quantitative data was analysed in Stata® 16.1 (StataCorp, The College Station, Texas, USA). Descriptive statistics for all variables as mean and standard deviation or frequency as determined by the type and distribution of the data were computed. Categorical variables between two groups were compared using Chi-square statistics and the distribution of continuous variables were compared using Wilcoxon rank sum test.
To assess the risk of selection bias and to estimate the similarity of the groups, we compared responses to overlapping questions within the OFTT and the follow-up survey.

For peer review only

## Qualitative data

To explain the quantitative results, we explored the experience of tool use by the patients qualitatively. Following quantitative data analysis, an interview guide was created and adapted iteratively.

## Purposeful and quota sampling

We purposefully sampled participants from those that had firstly, utilized our OFTT, secondly, had taken part in the follow-up survey and thirdly, had consented to a follow-up interview. We included participants of all age groups (quota) to ensure inclusiveness.

## Sample Size

Many experts suggest saturation as central to qualitative sampling [20]. In this study we aimed for both data saturation and rich and detailed narratives and achieved this with 19 key informants from all age groups (see Table 1).

## Data collection

Due to COVID-19 concerns, video rather than face to face interviews were held with most participants in September 2020. A combination of video and telephonic interviews were conducted with three participants who had technical challenges and a telephone only interview was held with one lady, aged above 65, who had no computer access. Three face to face interviews were held with three key informants: one that was a hospital health care worker, and two key informants who worked close to Bern university hospital. A semi-structured interview guide informed by the quantitative results was used (see supplemental file 2). This was adapted iteratively throughout the data collection period. Two qualitative researchers sat in each session fielding questions in turns. All interviews were conducted in German by two researchers fluent in both English and German. The interviews lasted between 45 minutes to one and a half hours. Two audio-recorders were used in each session. All participants gave individual written consent as well as oral consent to the recording at the beginning of each session. See Table 1 for summary of Key Informants.

For peer review only

**Table 1. Key Informant summary**

| Age-group | Males | Females | Total |
|---|---|---|---|
| 18-29 | 1 | 2 | **3** |
| 30-45 | 2 | 2 | **4** |
| 46-64 | 3 | 4 | **7** |
| 65+ | 4 | 1 | **5** |
| **Total** | **10** | **9** | **19** |

## Data analysis

Audio recordings were transcribed, analysed and triangulated with quantitative data results. Qualitative narratives were obtained to explain quantitative results as well as to explore utility of OFTT to patients as well as elicit recommendations to make online tools more useful and inclusive. A grounded theory approach was utilized. Concepts were identified from collected data and compared iteratively. These concepts were grouped into categories and culminated into the identified themes.

**Measures to ensure trustworthiness of data:** To ensure dependability, data collection and analysis were performed iteratively, continuously adjusting our interview guide to capture newly emerging themes. Throughout data collection, two qualitative researchers kept reflexive journals and debriefed at the end of each interview. To ensure transferability, a thick description of participants, context and data collection process has been outlined. Data was managed and analysed with the aid of MAXQDA2018.

## Ethics approval

The local ethics committee of the Canton of Bern, Switzerland, deemed this project a quality evaluation study and waived the need for full ethical review (Req-2020-00289) on the 23rd of March 2020.

## Patients and Public Involvement statement

Patients and public were not involved in the design, conduct, reporting or dissemination of this research since the OFTT was set up as an emergency response to the pandemic.

For peer review only

# RESULTS

## Quantitative results

In total, n=6,272 completed assessments of the OFTT were recorded on the website during the study period from March 2nd, 2020 to May 12th, 2020. This question asked OFTT users what they would have done had the OFTT not existed. The question was answered by 97.6% (3953/4049) of the users as follows: 40.2% (1626/4049) would have contacted the GP or visited a hospital had the tool not existed; furthermore, 16.4% (665/4049) would have contacted a hotline.
In the OFTT, 25.6% (1,608/6272) of assessments received a recommendation to test for COVID-19 during the study period. In the follow-up survey question, "Did the online tool recommend you to test for COVID-19?" -31.8% (56/176) answered, yes.
In the OFTT, 13.2% (564/4270) of OFTT users reported being over 65 years of age. The variable age was only included and mandatory during some phases of the study period in accordance with the FOPH guidelines, that changed frequently. This resulted in 4270 assessments with data on age. In the follow-up survey, 17.6% (31/176) reported being over 65 years.

A link to the online follow-up questionnaire was sent to 560 participants that consented to a follow-up survey by providing a valid e-mail address. The online questionnaire was filled out by 37.9% (212/560) of the participants; 31.4% (176/560) completed the whole questionnaire and were included in the analysis (all 22 questions-see supplement). An overview of socio-demographic characteristics of participants of the follow-up survey are presented in Table 2.

For peer review only

| | Total | (n=176) | Female | (n=101) | Male | (n=75) | P-value* |
|---|---|---|---|---|---|---|---|
| **Age [mean, SD]** | 50.1 | [±15.4] | 45.9 | [±14.1] | 55.7 | [±15.4] | <0.001 |
| **Education** | | | | | | | |
| Not want to answer | 6 | [3.4] | 3 | [3.0] | 3 | [4.0] | |
| University | 120 | [68.2] | 67 | [66.3] | 53 | [70.7] | |
| Higher secondary school | 27 | [15.3] | 17 | [16.8] | 10 | [13.3] | |
| Lower secondary school | 23 | [13.1] | 14 | [13.9] | 9 | [12.0] | 0.871 |
| **Income per month** | | | | | | | |
| Not want to answer | 29 | [16.5] | 17 | [16.8] | 12 | [16.0] | |
| <4000 CHF | 26 | [14.8] | 20 | [19.8] | 6 | [8.0] | |
| 4000 - 6000 | 42 | [23.9] | 27 | [26.7] | 15 | [20.0] | |
| >6000 | 79 | [44.9] | 37 | [36.6] | 42 | [56.0] | 0.037 |
| **Work** | | | | | | | |
| Not want to answer | 33 | [18.8] | 14 | [13.9] | 19 | [25.3] | |
| Employed | 106 | [60.2] | 64 | [63.4] | 42 | [56.0] | |
| Self-employed | 24 | [13.6] | 13 | [12.9] | 11 | [14.7] | |
| Unemployed | 3 | [1.7] | 3 | [3.0] | 0 | [0.0] | |
| Lost work (Covid-19) | 1 | [0.6] | 1 | [1.0] | 0 | [0.0] | |
| Student/trainee | 9 | [5.1] | 6 | [5.9] | 3 | [4.0] | 0.236 |
| **Insurance** | | | | | | | |
| Don't know | 5 | [2.8] | 3 | [3.0] | 2 | [2.7] | |
| General | 68 | [38.6] | 39 | [38.6] | 29 | [38.7] | |
| Telemedicine | 12 | [6.8] | 6 | [5.9] | 6 | [8.0] | |
| GP | 83 | [47.2] | 47 | [46.5] | 36 | [48.0] | |
| Other | 8 | [4.5] | 6 | [5.9] | 2 | [2.7] | 0.859 |
| **Nationality** | | | | | | | |
| Not want to answer | 1 | [0.6] | 1 | [1.0] | 0 | [0.0] | |
| Switzerland | 147 | [83.5] | 80 | [79.2] | 67 | [89.3] | |
| Germany | 13 | [7.4] | 8 | [7.9] | 5 | [6.7] | |
| French | 1 | [0.6] | 0 | [0.0] | 1 | [1.3] | |

For peer review only

| | | | | | | | |
|---|---|---|---|---|---|---|---|
| Italy | 3 | [1.7] | 2 | [2.0] | 1 | [1.3] | |
| Other Europe | 4 | [2.3] | 3 | [3.0] | 1 | [1.3] | |
| Other | 7 | [4.0] | 7 | [6.9] | 0 | [0.0] | 0.202 |

**Table 2. Socio-demographic table of participants of follow-up survey**

* Chi-squared for categorical variables and Wilcoxon rank sum test for continuous variables; data are total number and percentage if not mentioned otherwise

The survey revealed that 84.7% (149/176) followed the tool recommendations and stayed at home thereby reducing the work-load of GPs and hospitals. Information about the utilization of the OFTT, specifically which information was searched for, how subjects found the tool, and information about satisfaction with the tool is presented in Table 3.

**Table 3. Online forward triage tool use**

| | Total (n=176) | [%] |
|---|---|---|
| Information searched | | |
| Information on COVID-19 symptoms | 97 | [55.1] |
| How to cope with symptoms | 4 | [2.3] |
| To know when to consult a doctor | 36 | [20.5] |
| To know more on testing criteria | 32 | [18.2] |
| To know where to test | 7 | [4.0] |
| Mode of referral | | |
| Referral by family doctor | 9 | [5.1] |
| Online search | 113 | [64.2] |
| Recommendation by peers | 17 | [9.7] |
| Hotline | 2 | [1.1] |
| Other | 35 | [19.9] |
| Satisfaction with information | | |
| Helpful | 154 | [87.5] |
| Not comprehensive | 17 | [9.7] |
| Not clear | 5 | [2.8] |

We present additional factors that may have influenced how individuals coped during the coronavirus pandemic, their use of the OFTT and adherence to OFTT recommendations. Overarching topics that were asked included the influence of the media, fear and uncertainty,

For peer review only

For peer review only - http://bmjopen.bmj.com/site/about/guidelines.xhtml

and reasons for adherence to the recommendation (see table 4). All questions and answers from the follow-up questionnaire are attached. See supplemental file 1.

**Table 4. Additional factors**

| | Total (n=176) | [%] |
|---|---|---|
| Estimated influence of media | | |
| Helpful | 81 | [46.0] |
| Confusing | 47 | [26.7] |
| No trust in media as source of information | 25 | [14.2] |
| Other | 23 | [13.1] |
| Influence of OFTT on fear and anxieties | | |
| Reassured | 73 | [41.5] |
| No reassurance | 13 | [7.4] |
| Increased fears and anxieties. | 6 | [3.4] |
| Not worried before OFTT use | 84 | [47.7] |
| Reasons for following the recommendation (n=149) | | |
| Trust in tool | 60 | [40.3] |
| Information congruent with media | 20 | [13.4] |
| Comparison with FOPH recommendation | 53 | [35.6] |
| Reassurance by others | 7 | [4.7] |
| Other | 9 | [6.0] |

## Qualitative findings

Seven overarching themes on the utility of the OFTT emerged during the qualitative interviews. These are used to structure the report of our findings, i.e., i) accessibility of the tool, ii) user-friendliness of the tool, iii) utility of the tool as an information source, iv) utility of the tool in allaying fear and anxiety, v) utility of the tool in decision making (test or not to test), vi) utility of the tool in reducing onward transmission-cross infection, and vii) utility of the tool in reducing health system burden. The qualitative findings are summarised in Table 5.

For peer review only

**Table 5. Summary of qualitative themes**

| Theme | Category | Unit meaning |
|---|---|---|
| **Accessibility** | Online search | Appeared but not on the top of google search<br>Advertise tool in future |
| | Unreachable for some | Include telephonic services to reach the elderly<br>Tool buddies |
| **Utility as a reliable information source** | COVID-19 Symptoms | Cough was a main symptom<br>Symptom description like type of cough and severity of fever etc. was not possible<br>Test or do not test decision was arbitrary-how the decision was arrived at was not clear e.g., 95% probability test or 5% probability do not test |
| | Testing info and centres missing | Information on when to call doctor was not clear e.g., fever above 39 degrees for 4 days -call doctor<br>List of where to test and contact numbers were missing |
| **Utility in decision making** | Followed recommendations | Trust- the university hospital is a trusted institution |
| | Did not follow recommendations | Fear of a positive result and the resultant consequences<br><br>Cost of test<br>Test shortage<br>GP refusing patients to test -hysteria |
| **Utility in allaying fear and anxiety** | Reassured some | Fear and anxiety allayed after tool use |
| | Person contact | An online tool is still an online tool - recommendations seen as not having a lot of weight<br><br>A talk with a general practitioner (GP)- debriefing after tool use could have put them at ease |
| | Testing | Testing in itself is reassuring -make test available to all who are anxious |
| | Friends and family as a resource | Many relied on family and friends to deal with fear- social circle still a major source of support |
| | Increased anxiety in some | |

For peer review only

| | | |
|---|---|---|
| | | High risk label unsettled some |
| **Utility in reducing health system burden** | Many stayed at home | Recommendations followed- stay at home<br>Some called Insurance companies |
| **Utility in reducing onward transmission** | Call GP before a visit | Most called GP ahead of visit |
| **Systems thinking** | Utility of tool is dependent upon other health system and societal components<br><br>Fear of a positive test<br>-rather not know | Participants told by tool to test only to be told that there are no tests (shortages)<br>Fear of a positive test<br><br>Media misinformation of painful test influenced some not to test-work with media<br><br>Economic factors like cost of test influenced some not to test<br>A new life-threatening disease in a population is associated with psycho-social and behavioural issues that need to be taken into account |

**Theme 1: Accessibility of the tool**

The accessibility of the tool emerged as very important. Many participants suggested to advertise the tool to make it more accessible as revealed below:

> *"I did not know of the existence of tool (an accidental internet search led the key informant to the tool). Please advertise tool on TV and to Insurance companies."* -Key Informant 15

The older people seem willing to embrace technology and were prepared to use it. However, they stated that they needed help with practical application at times as revealed below;

> *"Provide telephone services for the elderly and a contact person, a GP so one can ask questions if unsure."* -Key Informant 14

**Theme 2: User-friendliness of the tool**

Most participants could not remember the tool immediately due to the time lapse from the tool usage to interview. After being shown the tool once again, the header only, many cited it as having been easy and simple to follow with the language being clear and the length acceptable.

**Theme 3: Utility of the tool as an information source**

The novel nature of COVID-19 infection left many scrambling for knowledge of the disease. Many health care providers were inundated with phone calls. One participant said the following;

For peer review only - http://bmjopen.bmj.com/site/about/guidelines.xhtml

*"The tool provided information on symptoms but did not have a list of testing centers. The recommendations said call GP before visit but there was no number to call."* -Key Informant 1

*"Telemedicine could play a better information spreading role – media spread fear and misinformed people for example mask use vs no mask."* -Key Informant 15

**Theme 4: Utility of the tool in allaying fear and anxiety**

Many participants interviewed reported being reassured after tool use. Others cited being more anxious after tool use due to terminology and language and many suggested that a person, a doctor be available after tool use for closure. Participants revealed the following;

*"Wording of tool could be adapted – a friend aged 65, a diabetic, became depressed after using tool and getting the high-risk patient classification. He needed a psychiatrist to cope. Rather ask how are you, do you take any medication, which ones? Mentioning conditions seem to increase anxiety."* -Key Informant 17

*"I felt discriminated against by tool-differentiate between a health 73-year-old with no chronic illnesses and a 50-year overweight diabetic."* -Key Informant 13

**Theme 5: Utility of the tool in decision making process (to test or not to test)**

Many participants cited trust in our university hospital (Insel) as one of the main reason participants followed the recommendations. Some participants revealed the following;

*"Insel has a good name and trusted the tool."* -Key Informant 16

*"Coordination is needed for FOPH and Insel to speak in one voice."* -Key Informant 17

Juxtaposed and not necessarily contradicting the quantitative survey, where trust was reported as the main reason for following the recommendations, most of the participants cited shortages of tests, improved symptoms, cost of test, misinformation that the test was painful and fear of a positive result as reasons for not testing. Of utmost importance were GPs who viewed the test request by online tool users as being hysteric. Below is what some participants said:

*"I read scientific papers to inform oneself and then decided."* -Key Informant 8

*"Remember recommendations from an online tool have less weight than recommendations from a doctor – there is no person behind this and so many might have taken the tool and went further to contact own GP"*- Key Informant 8

*"I wished to see an algorithm that said something like, "the probability of you having COVID-19 is 75% test or 25% do not test."*-Key Informant 5

**Theme 6: Utility in reducing the potential for onward transmission- cross infection**

The tool recommended all participants to call the health care provider ahead of visit and most of them did. A reason some participants might not have called the testing centres ahead of a visit could be that the tool itself did not provide a list of contact numbers-a short coming that was rectified in the second generation OFTT.

**Theme 7: Utility of tool in reducing health system burden**

Social distancing, isolation and quarantine were among the recommendations made to reduce the spread of COVID-19. Most of the participants stayed at home. One participant said the following;

> *"I followed recommendations and stayed at home. However, home testing should be provided if people should stay at home. Engage Spitex [organization for outpatient and home-based care in Switzerland] in future pandemics and work with them."* -Key Informant 6

For peer review only

# Discussion

This study quantitatively assessed the effects and confirmed the utility (qualitatively) of a COVID-19 online forward triage tool by exploring patient perspectives. We further elaborate on areas for improvement as well as share lessons learned for policy makers. Qualitatively, seven overarching themes emerged namely i) accessibility of tool, ii) user-friendliness of tool, iii) utility of tool as an information source, iv) utility of tool in allaying fear and anxiety, v) utility of tool in decision making (test or not to test), vi) utility of tool in reducing the potential for onward transmissions (preventing cross infection) and vii) utility of tool in reducing health system burden.

*Accessibility of OFTT*

One of the objectives of our OFTT was to provide an easily accessible, reliable and up to date information platform for professionals and the public. The tool was not advertised commercially; hence it did not appear at the top of the google search and many participants cited coming across the tool accidentally. Information about the tool was only disseminated via the hospital website and hospital communication to local doctors.

Despite the above -mentioned shortcoming, our findings revealed that the tool was accessible to both genders and all age groups including the elderly. In line with other studies,[21] the elderly seem ready to embrace online tools, contradicting other studies.[10,17] Contradicting our findings, one study revealed that it's the young and highly educated patients that tend to use symptom checkers or OFTTs.[22]

Despite the revealed readiness of the elderly to embrace technology, key informants suggested keeping the use of telephonic services for the elderly as an option in telemedicine. Further supporting these findings, nurse triage lines (telephone) have been proven effective in this COVID-19 pandemic context in the US and in Canton Vaud, Switzerland.[10,23] Others suggested having a list of tool buddies reachable by phone, that links people that have used the tool before and are willing to be contacted by a new user, that might be experiencing challenges in using the OFTT. With regards to reaching the low education and low-income group, additional studies need to be done as those who earned less than CHF 4000 were not necessarily lowly educated but PhD and post doc students, concurring with findings elsewhere.[24]

*User-friendliness of OFTT*

Most of the participants could not recall tool, but after showing them tool header only, many cited tools as user-friendly, easy, with a clear language and an acceptable length, concurring with a study that was conducted elsewhere.[25] In support of our findings, online tools have been shown to be risk averse as compared to health care professionals and the users have expressed high levels of satisfaction.[22] The optimal amount of time spent filling in OFTT questionnaires nor the optimal number of questions an OFTT should ask in general, is still unclear[26] and warrants further studies.

*Utility of OFTT as an information source*

Overall, the tool was very useful in providing information on signs and symptoms. Information on where to test (list with contact numbers), how to self-care, when to contact a GP were cited by some as shortcomings and ought to be included to make the tool comprehensive in future. Information challenges with OFTTs have also been reported elsewhere.[27,28] This finding underlines the need to have an option to talk directly to a GP after OFTT use so as to debrief.

Further information or links to comprehensive and reliable sources with information on how to self-care and when to contact a GP or health care centre emerged as gaps that need to be incorporated in COVID-19 OFTTs so as to increase their utility as information sources. The

For peer review only - http://bmjopen.bmj.com/site/about/guidelines.xhtml

majority of our participants were highly educated, and this segment of the population seems to inform itself, by consulting a variety of scientific sources as well as keeping abreast with the FOPH announcements. In the context of a novel infection, where guidelines change quickly and continuously, the credibility of the tool to the highly educated, could be enhanced by stipulating sources of information and referencing and dating the FOPH criteria informing the tool.

*Utility of OFTT in allaying fear and anxiety*

For most of the participants, the tool was effective in allaying their fear and anxiety. Many wished a human presence, a doctor to debrief with after the online tool use as mentioned above. There was however, a downside for some that felt labelled as being high risk. For this group, the tool had a negative effect and increased their anxiety. Other studies have revealed similar effects.[29,30] This raises the issue of language and terminology use in such tools. Bearing in mind that COVID-19 is a novel condition, not well understood and considered fatal, the impact of a high-risk label should not be underestimated, including discrimination. Concurring with our findings, COVID-19 stigma has been reported elsewhere [31]. Many participants reported fear of a positive test result and the consequences thereof, concurring with findings from elsewhere. [32,33] Further concurring with our findings, lasting psychological consequences that last beyond the COVID-19 infection itself have also been revealed.[31] This raises the question of psychological readiness to deal with such a diagnosis. Emerging studies have reported COVID-19 patients as having psychiatric related conditions post infection, further concurring with our study.[34,35]

*Utility of OFTT in facilitating decision making*

The tool was useful in assisting patients in decision making particularly not to test. Trust in the institution proved pivotal as many followed recommendations simply because they trusted the source of the tool, our university hospital. Studies elsewhere concur with our findings.[36,37] On the other hand, some of those that got the recommendation to test did not do so due to a myriad of reasons as revealed above. In addition, the cost of the test (CHF 180 at the time), shortages of tests and fear of a positive result and the resultant consequences of isolating, stigma etc. further influenced decisions not to test. A low income was found not to be a reliable socio-economic status proxy in our study. Most low-income participants were PhD students and post-docs who cited various reasons for not following recommendations. Many told us how they sought and read scientific evidence to inform themselves and this, rather than the recommendations, guided their decision making. In line with our findings, salary is not a good proxy for socio-economic status among online tool users.[24] A shortcoming in this regard, was the missing information on how the tool arrived at the recommendation to test or not to test e.g. algorithm used[19] something some key informants wished to know. The issue of safety concerns with regards to specificity of digital tool algorithms has also been reported elsewhere.[38]

*Utility of OFTT in preventing onward transmission- cross infection*

The tool proved useful in preventing cross infection concurring with findings elsewhere.[19] Most participants who were told to stay at home did so, reducing mobility and exposure. Most of the participants called the GP practice ahead of time. That gave the GP practices time to ensure that the suspect patient did not mix with other patients, thereby reducing the potential for onward transmission (cross infection).[19]

For peer review only

*Utility of OFTT in reducing health system burden*

Our primary hypothesis was that such an OFTT reduces the health system burden. Most of the participants who used the tool would have called their GP or visited the hospital. OFTT use effectively kept these worried participants at home and out of the doctors' offices and hospitals, effectively reducing the health system burden. Contradicting our findings, research from elsewhere has produced inconclusive and sometimes contradicting evidence.[28,39] Further studies in different contexts are therefore called for. Further contradicting our findings,, another study reported that symptom checkers` triage capabilities are not greater than that of an average lay person.[40] In fact the convenience of telemedicine has also been associated with increased utilization of services, increasing work load and health care spending.[41]

**Recommendations and lessons learned**

Our study demonstrated the effects and utility of a COVID-19 OFTT. The assessment of an OFFTT is important but not without challenges. Below are some of the lessons worth sharing with both health care providers and policy makers as subsequent waves sweep across Europe;

- Most of the participants had challenges remembering the tool. Immediate feedback e.g., in one minute, please rate this tool, or three open questions; please tell us how useful this tool was with regards to i) accessibility of tool, ii) utility of tool as an Information source, ii) utility of tool in facilitating your decision making could be more effective. Data protection concerns and the need to keep barriers to use as low as possible, could stand in the way of this approach.
- The tool simply instructed patients to test or not to test, an arbitrary decision, without shedding light on how the decision was made. Patients wish to see an algorithm that says something like, "*the probability of you having COVID-19 is 75% test or 25% do not test.*"
- Many participants said, "*bear in mind that online tool recommendations have less weight than recommendations from a GP.*" Additional caution is needed in language and terminology use as some patients that felt labelled by tool as high risk, had negative outcomes. Ensuring access to a doctor to debrief with after such tool use is advisable. Retired doctors who are still willing to make a contribution to the society, could play such a role.
- Many participants found the tool by accident; hence it is advisable to advertise tool on social media platforms, billboards, TV, radio and could make it appear at the top of google search. In addition, taking the tool to the people e.g., through road shows could be a useful strategy to reach the old people – if they do not come to the tool, take the tool to the people.
- Many participants compared the tool recommendations with what the Federal Office of Public Health (FOPH) recommended at the time. Having a tool link on FOPH website that stipulates and references the FOPH criteria informing the tool, could increase trust in tool and acceptability. Coordination between FOPH, university hospitals, and other medical professional bodies is recommended to further enhance trust in the tool.
- Many elderly people are willing to embrace telemedicine, but challenges persist. Telephone and voice activated system for the older population or call centers to serve this group, are still needed (taking heed of unreachable and unanswered calls) during this transitional phase.
- Most participants found media confusing – telemedicine could play a better information spreading role, sifting through the noise and offering scientific based

For peer review only

recommendations. For many, the media spread fear and misinformed people in many instances.

- The OFTT lacked information on where to test (contact list of testing centres), how to self-care, how to manage symptoms and when to contact a doctor-addressing these shortcomings could improve the utility of OFTTs. Our results underline the importance of not offering a telehealth tool as a stand-alone product, but to integrate it into an overall concept with links to credible reliable sources.
- Systems thinking-refers to the ability to see interconnectedness in a system with a dysfunction in one part affecting other parts and consequently outcomes. Our study revealed the reasons patients did not follow the recommendation to test, as multipronged. Attention has to be paid to supply chain issues, as test shortages affected outcomes. The cost of a test and the fear of a positive result additionally emerged as hindrances to testing. This calls for systems thinking. Noteworthy, is the reaction of GPs who labelled OFTT users who asked for a COVID-19 test as hysteric. This does not only reveal that the pandemic caught everyone by surprise, but also demonstrates the need to involve, collaborate with and win the local health care providers-policy implementers, like GPs and Spitex (home based nursing), to enhance tool utility as well as ensure positive outcomes
- One key informant suggested having patients who had recovered from COVID-19 act as champions to share their illness experience, and motivate the public to take preventive measures and take the disease seriously-an approach that was also effective in HIV prevention and coping strategies.

## Strengths and limitations

Many online tools have been developed during the COVID-19 pandemic. The effects and utility of these tools however have not been assessed. *Coronatest.ch* was one of the first COVID-19 OFTTs in Switzerland. Our study could become the base line for studies that assess the effects and utility of such online tools. The identified themes namely i) accessibility of tool, ii) user-friendliness of tool, iii) utility of tool as an information source, iv) utility of tool in allaying fear and anxiety, v) utility of tool in decision making (test or not to test), and vi) utility of tool in reducing onward transmission-cross infection, vii) utility of tool in reducing health system burden, could serve as a framework for assessing OFTT utility (follow-up paper). The mixed method sequential explanatory design gave us a better understanding of OFTTs, their effects measured quantitatively and utility explained with the aid of qualitative findings. We did not simply report the effects but could also explain why the results were that way, generating a holistic picture of the phenomenon.

The selection of the participants in our study carries the risk of a selection bias. Perspectives of those that do not use online tools are missing and should be explored in further studies. In addition, only a limited number of OFTT users took part in our study. This selection bias cannot, to the best of our knowledge, be prevented due to data protection regulations which impose a voluntary participation and prohibit a technically possible automatic tracking of participants. Another way to avoid this possible selection bias would be to make the use of such a tool conditional on participation in the study. We have deliberately decided against this procedure for ethical reasons, in order to make our OFTT accessible to as many users as possible and to keep barriers as low as possible. In addition, mandatory entry of personal data in OFTT for study purposes would also discourage individuals from using the tool and thus trigger a new bias. Our comparison of overlapping questions between the OFTT and the follow-up survey

For peer review only

can at least help to estimate the similarities within the two groups. For both questions, the percentages are comparable and can help in estimating the similarity of the groups.

Another limit of our study is the relatively long duration between the use of tool and the qualitative interviews. This could have introduced a certain degree of recall bias. As with all online tools, we cannot confirm the accuracy of the data entered. In particular, we cannot say for sure whether the OFTT users used the tool to assess own symptoms or for other reasons, such as curiosity, fear or uncertainty about how to deal with the novel infection. Likewise, multiple use, trial runs or use of tool by a health care worker on behalf of patients, relatives and friends are all possible. Socio-economic status might have introduced a selection bias in our study since most of the participants had a higher education. Income emerged not to be a good proxy for assessing socio-economic status. Other instruments, apart from income are therefore needed to assess socio-economic status.  Additionally, an on online assessment cannot fully replace a (polymerase chain reaction) PCR test as some asymptomatic people might be positive and those with COVID-19 specific symptoms might be suffering from a different disease.[5] In our mind, the data still sheds light on the effects and utility of such an online tool and the recommendations given could guide other OFTT developers as the third wave sweeps across Europe. As the study was conducted with a specific OFTT, transferability of our results to other OFTTs is not necessarily a given. Given the limited evidence on the use of OFTTs, the results, in particular the qualitative component of the study, could be of value to other OFTT developers, with particular regards to utility and accessibility issues. Further studies with other OFTTs outside the COVID-19 context are recommended so as to increase transferability and improve the utility of OFTTs in the current third wave, future pandemics and other health care settings.

For peer review only

# Conclusion

OFTT use has increased greatly during this pandemic. The effects and utility of such tools however, have not been widely assessed. That makes our study, one of the firsts, in assessing effects and utility of a COVID-19 OFTT. Our study revealed that an OFTT does not only reduce the health system burden but can also serve as an information source, reduce anxiety and fear, reduces potential for onward transmission and facilitate decision making.

For peer review only

# DECLARATIONS

**Acknowledgement:** Emergency telemedicine at University of Bern, Switzerland is supported by an endowed professorship by the Touring Club Switzerland.

**Author contributions:** Study design and idea: J.M., A.M, R.S, T.C.S., M.M, M.E.R, P.J, W.E.H.; Data extraction and preparation: R.S., M.M, A.M; Qualitative interviews: J.M., R.S., A.M.; Statistical analysis: M.M.; Qualitative analysis: JM. Writing of first draft: J.M., A.M, M.M.; Revision of the final draft and final approval: all authors; Supervision: T.C.S., W.E.H.; Project administration: T.C.S., W.E.H.

**Category of article:** original article

**Running title:** Usage of online tools during the COVID-19 pandemic

**Source of funding:** Swiss National Science Foundation (Project ID: 196615)

**Conflict of interest:** This manuscript is partially founded by the Swiss National Science Foundation (Project ID: 196615). The founder has no influence on the content of the manuscript or the decision to publish it.
TCS holds the endowed professorship for emergency telemedicine at University of Bern, Switzerland. The founder has no influence on the research performed, the content of any manuscript or any decision to publish. All other authors have nothing to disclose.

**Data sharing**
Due to the nature of the study (OFTT) participants did not agree for their data to be shared publicly. The data to support findings are available. Please contact corresponding author JM.

For peer review only

# REFERENCES


1. Higgins-Dunn N. Coronavirus cases surpass 25 million as pandemic strains nations around the world. CNBC. Published August 30, 2020. Accessed August 31, 2020. https://www.cnbc.com/2020/08/30/global-coronavirus-cases-surpass-25-million-as-pandemic-strains-nations-around-the-world.html

2. Switzerland Coronavirus: 42,393 Cases and 2,010 Deaths - Worldometer. Accessed September 1, 2020. https://www.worldometers.info/coronavirus/country/switzerland/

3. Germany Coronavirus: 244,792 Cases and 9,371 Deaths - Worldometer. Accessed September 1, 2020. https://www.worldometers.info/coronavirus/country/germany/

4. United Kingdom Coronavirus: 335,873 Cases and 41,501 Deaths - Worldometer. Accessed September 1, 2020. https://www.worldometers.info/coronavirus/country/uk/

5. Munsch N, Martin A, Gruarin S, et al. *A Benchmark of Online COVID-19 Symptom Checkers*. Health Informatics; 2020. doi:10.1101/2020.05.22.20109777

6. Hollander JE, Carr BG. Virtually Perfect? Telemedicine for Covid-19. *N Engl J Med*. 2020;382(18):1679-1681. doi:10.1056/NEJMp2003539

7. Schrager J, Schuler K, Wright D, et al. Development and Usability Testing of a Web-based COVID-19 Self-triage Platform. *West J Emerg Med*. 2020;21(5). doi:10.5811/westjem.2020.7.48217

8. Hamm JM, Greene C, Sweeney M, et al. Telemedicine in the emergency department in the era of COVID-19: front-line experiences from 2 institutions. *J Am Coll Emerg Physicians Open*. n/a(n/a). doi:10.1002/emp2.12204

9. Kellermann AL, Isakov AP, Parker R, Handrigan MT, Foldy S. Web-Based Self-Triage of Influenza-Like Illness During the 2009 H1N1 Influenza Pandemic. *Ann Emerg Med*. 2010;56(3):288-294.e6. doi:10.1016/j.annemergmed.2010.04.005

10. Spaulding AB, Radi D, Macleod H, et al. Design and Implementation of a Statewide Influenza Nurse Triage Line in Response to Pandemic H1N1 Influenza. *Public Health Rep*. 2012;127(5):532-540.

11. Gann B. Giving patients choice and control: health informatics on the patient journey. *Yearb Med Inform*. 2012;7:70-73.

12. Joshi AU,, Lewiss RE. Telehealth in the time of COVID-19. *Emerg Med J*. Published online 2020. doi:10.1136/ emermed-2020-209846

13. Judson TJ, Odisho AY, Neinstein AB, et al. Rapid design and implementation of an integrated patient self-triage and self-scheduling tool for COVID-19. *J Am Med Inform Assoc JAMIA*. 2020;27(6):860-866. doi:10.1093/jamia/ocaa051

14. Amir-Behghadami M, Janati A. Iranian national COVID-19 electronic screening system: experience to share. *Emerg Med J*. 2020;37(7):412-413. doi:10.1136/emermed-2020-209806


For peer review only

15. Meyer AND, Giardina TD, Spitzmueller C, Shahid U, Scott TMT, Singh H. Patient Perspectives on the Usefulness of an Artificial Intelligence-Assisted Symptom Checker: Cross-Sectional Survey Study. *J Med Internet Res*. 2020;22(1):e14679. doi:10.2196/14679

16. Why do many seniors have trouble using technology? No Isolation. Accessed September 8, 2020. https://www.noisolation.com/global/research/why-do-many-seniors-have-trouble-using-technology/

17. Conger K, Griffith E. As Life Moves Online, an Older Generation Faces a Digital Divide. *The New York Times*. https://www.nytimes.com/2020/03/27/technology/virus-older-generation-digital-divide.html. Published March 27, 2020. Accessed September 8, 2020.

18. Digital inclusion for low-skilled and low-literate people: a landscape review - UNESCO Digital Library. Accessed September 8, 2020. https://unesdoc.unesco.org/ark:/48223/pf0000261791

19. Millenson ML, Baldwin JL, Zipperer L, Singh H. Beyond Dr. Google: the evidence on consumer-facing digital tools for diagnosis. *Diagn Berl Ger*. 2018;5(3):95-105. doi:10.1515/dx-2018-0009

20. Morse JM. Determining Sample Size. *Qual Health Res*. 2000;10(1):3-5. doi:10.1177/104973200129118183

21. Chambers D, Cantrell AJ, Johnson M, et al. Digital and online symptom checkers and health assessment/triage services for urgent health problems: systematic review. *BMJ Open*. 2019;9(8). doi:10.1136/bmjopen-2018-027743

22. Chambers D, Cantrell AJ, Johnson M, et al. Digital and online symptom checkers and health assessment/triage services for urgent health problems: systematic review. *BMJ Open*. 2019;9(8). doi:10.1136/bmjopen-2018-027743

23. CoronaCheck for healthcare professionals | CoronaCheck Unisanté. Accessed December 1, 2020. https://coronavirus.unisante.ch/en/professionnels

24. The not-so-straightforward measurement of socioeconomic status. The Dunn Lab. Accessed September 18, 2020. http://www.thedunnlab.com/updates/measuringses

25. Nelson CA, Pérez-Chada LM, Creadore A, et al. Patient Perspectives on the Use of Artificial Intelligence for Skin Cancer Screening: A Qualitative Study. *JAMA Dermatol*. 2020;156(5):501-512. doi:10.1001/jamadermatol.2019.5014

26. What questions are priority areas for primary care during the COVID-19 pandemic? A rapid question generation and prioritisation exercise. CEBM. Accessed July 14, 2020. https://www.cebm.net/covid-19/what-questions-are-priority-areas-for-primary-care-during-the-covid-19-pandemic-a-rapid-question-generation-and-prioritisation-exercise/

27. Advantages and Disadvantages of using Symptoms Checker ⋆ The Stuff of Success. Accessed October 19, 2020. https://thestuffofsuccess.com/2018/04/08/advantages-and-disadvantages-of-using-symptoms-checker/

28. Symptom Checkers and Self-Triage Tools in the Era of COVID-19: What's the Evidence for their Use? NODE.Health. Published April 17, 2020. Accessed September 18, 2020.

For peer review only

https://nodehealth.org/2020/04/17/symptom-checkers-and-self-triage-tools-in-the-era-of-covid-19-whats-the-evidence-for-their-use/

29. How Online Searches Fuel Health-Related Anxiety. ICANotes. Published May 7, 2019. Accessed October 19, 2020. https://www.icanotes.com/2019/05/07/how-online-searches-fuel-health-related-anxiety/

30. Van Riel N, Auwerx K, Debbaut P, Van Hees S, Schoenmakers B. The effect of Dr Google on doctor–patient encounters in primary care: a quantitative, observational, cross-sectional study. *BJGP Open*. 1(2). doi:10.3399/bjgpopen17X100833

31. The "Untold" Side of COVID-19: Social Stigma and Its Consequences in India - Prama Bhattacharya, Debanjan Banerjee, TS Sathyanarayana Rao, 2020. Accessed September 18, 2020. https://journals.sagepub.com/doi/full/10.1177/0253717620935578

32. How Fear Inhibits HIV Testing and Treatment in African Men. Contagion Live. Accessed October 19, 2020. https://www.contagionlive.com/view/how-fear-inhibits-hiv-testing-and-treatment-in-african-men

33. Desmon S. Study: Fear Keeps Men from Getting Tested for HIV. Johns Hopkins Center for Communication Programs. Published October 28, 2019. Accessed October 19, 2020. https://ccp.jhu.edu/2019/10/28/study-fear-keeps-men-from-hiv-testing/

34. More Than Half of COVID-19 Survivors in Study Reported Psychiatric Disorders. EcoWatch. Published August 4, 2020. Accessed September 18, 2020. https://www.ecowatch.com/covid-19-psychiatric-disorders-2646878306.html

35. Varatharaj A, Thomas N, Ellul MA, et al. Neurological and neuropsychiatric complications of COVID-19 in 153 patients: a UK-wide surveillance study. *Lancet Psychiatry*. 2020;7(10):875-882. doi:10.1016/S2215-0366(20)30287-X

36. Birkhäuer J, Gaab J, Kossowsky J, et al. Trust in the health care professional and health outcome: A meta-analysis. *PLoS ONE*. 2017;12(2). doi:10.1371/journal.pone.0170988

37. Brennan N, Barnes R, Calnan M, Corrigan O, Dieppe P, Entwistle V. Trust in the health-care provider–patient relationship: a systematic mapping review of the evidence base. *Int J Qual Health Care*. 2013;25(6):682-688. doi:10.1093/intqhc/mzt063

38. Anhang Price R, Fagbuyi D, Harris R, et al. Feasibility of Web-Based Self-Triage by Parents of Children With Influenza-Like Illness: A Cautionary Tale. *JAMA Pediatr*. 2013;167(2):112. doi:10.1001/jamapediatrics.2013.1573

39. EBSCO HEALTH. Enabling Patient Engagement with a Symptom Checker. Published online 2020:16.

40. Schmieding ML, Mörgeli R, Schmieding MAL, Feufel MA, Balzer F. Benchmarking Triage Capability of Symptom Checkers Against That of Medical Laypersons: Survey Study. *J Med Internet Res*. 2021;23(3):e24475. doi:10.2196/24475

41. Ashwood JS, Mehrotra A, Cowling D, Uscher-Pines L. Direct-To-Consumer Telehealth May Increase Access To Care But Does Not Decrease Spending. *Health Aff (Millwood)*. 2017;36(3):485-491. doi:10.1377/hlthaff.2016.1130

For peer review only

836 # List of figures

837 **Figure 1: Mixed-Methods Sequential Explanatory Study Design**

838 **Fig 2: OFTT triage**

839

840

For peer review only

**Figure 1: Mixed-Methods Sequential Explanatory Study Design.**

| Phase | Procedure | Product |
|---|---|---|
| **Collection of clinical data** | OFTT | Data about usage of the tool Contact data for later study phases |
| **Quantitative data collection** | Questionnaires | Numeric data |
| **Quantitative data analysis** | Statistical analysis | Descriptive statistics |
| **Connecting quantitative and qualitative data** | Purposefully selecting patients for interviews | Interview guide |
| **Qualitative data collection** | Individual in-depth interviews | Interview transcripts |
| **Qualitative data analysis** | Coding and thematic analysis | Codes and themes |
| **Integration of qualitative and quantitative results** | Interpretation and explanation of quantitative results with the aid of and qualitative findings | Implications for OFTT development |

For peer review only

**Fig 2 OFTT triage**

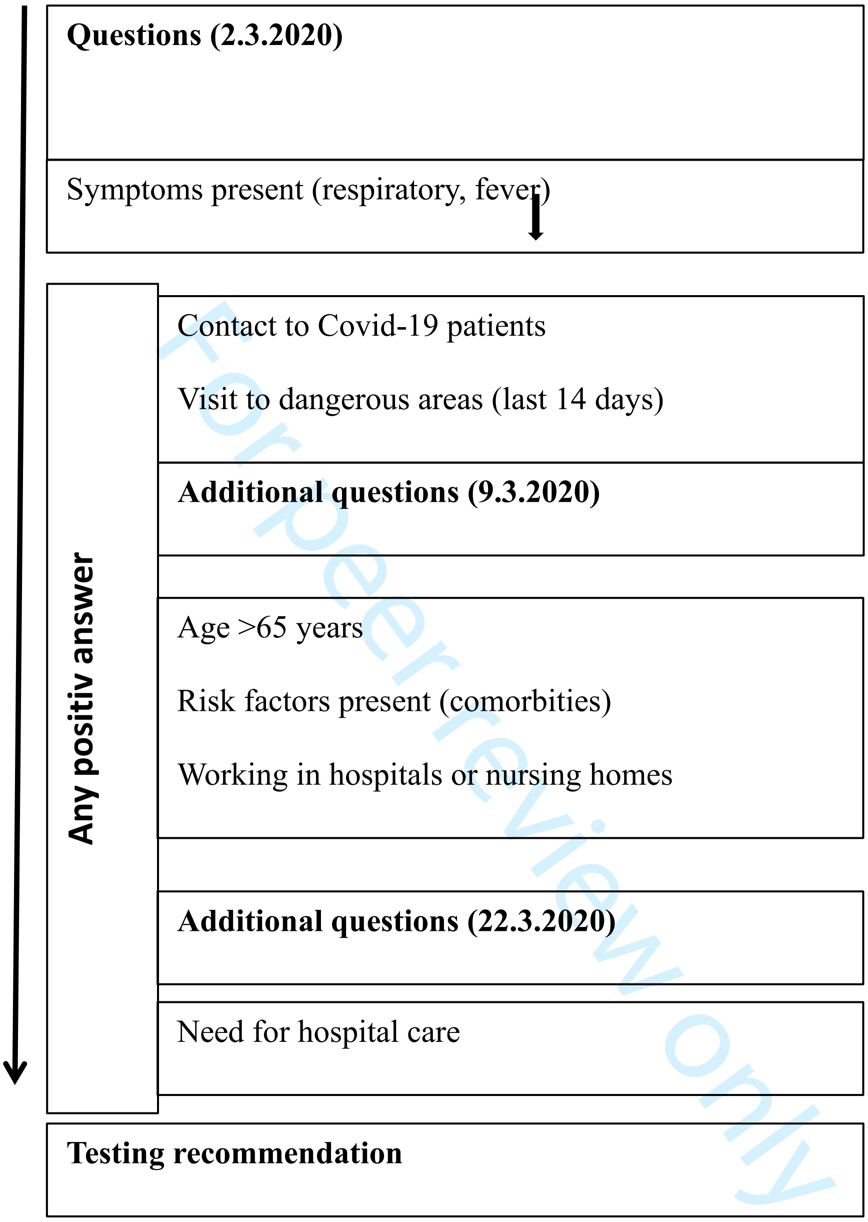

# Supporting information OFTT Questionnaire and results

## Supporting information 1. Usage of online tools during the COVID-19 pandemic: email survey

| | | | |
|---|---|---|---|
| v1 | **How did you get to this Online - Tool?** | | |
| | 1 - My family doctor advised me to use this tool. | 9 | 5.1% |
| | 2 - I found the information on the Internet. | 113 | 64.2% |
| | 3 - The website was recommended to me by family/friends. | 17 | 9.7% |
| | 4 - Via a telephone hotline. | 1 | 0.6% |
| | 5 - Other (Free text) | 35 | 19.9% |
| v2 | **Did you find the information that you needed?** | | |
| | 1 - Yes | 154 | 87.5% |
| | 2 - No, because the information was not comprehensive. | 17 | 9.7% |
| | 3 - No, because the information was not clear. | 5 | 2.8% |
| v3 | **What information did you search for? I wanted....** | | |
| | 1 - ... more information on COVID-19 symptoms | 97 | 55.1% |
| | 2 - ... more information on how to cope with symptoms | 4 | 2.3% |
| | 3 - ... to know when to consult a doctor | 36 | 20.5% |
| | 4 - ... to know more on testing criteria | 32 | 18.2% |
| | 5 - ... to know where to test | 7 | 4.0% |
| | 6 - Other | - | - |
| v4 | **Did the online tool recommend you to test for COVID-19?** | | |
| | 1 - Yes | 56 | 31.8% |
| | 2 - No | 120 | 68.2% |
| v5 | **Did you stick to the recommendations?** | | |
| | 1 - Yes | 149 | 84.7% |
| | 2 - No | 27 | 15.3% |
| v6 | **If you followed the Online - Tool recommendations, what made you do so?** | | |
| | 1 - I trust the website as a reliable information source. | 60 | 34.1% |
| | 2 - I compared the recommendations with recommendations from the media and took a decision. | 20 | 11.4% |
| | 3 - I compared the recommendations with those from FOPH (BAG) and took a decision. | 53 | 30.1% |
| | 4 - I sought advice from a person I trusted. | 7 | 4.0% |
| | 5 - Other, please specify: Free text | 9 | 5.1% |
| v7 | **In case you did not follow the recommendations, why did you not** | | |
| | 1 - I did not trust the website as a reliable source of information. | 1 | 0.6% |
| | 2 - The recommendations from the website differed from the media recommendations. | 2 | 1.1% |
| | 3 - I feared for my life and needed to consult a GP in person. | 6 | 3.4% |
| | 4 - Other, please specify: Free text | 18 | 10.2% |
| v8 | **Were your fears and anxieties allayed after visiting the website?** | | |
| | 1 - Yes, the information from the website reassured me. | 73 | 41.5% |
| | 2 - No, the information from the website did not reassure me. | 13 | 7.4% |

For peer review only

| | | | |
|---|---|---|---|
| | 3 - No, the information from the website increased my fears and anxieties. | 6 | 3.4% |
| | 4 - I was not worried. | 84 | 47.7% |
| v9 | **How did you cope with your fears? What helped you cope?** | | |
| | 1 - Free text | - | - |
| v10 | **In case you went to the GP, did you call ahead of time to notify them of your visit?** | | |
| | 1 - Yes | 115 | 65.3% |
| | 2 - No | 61 | 34.7% |
| v11 | **Did you get tested for Coronavirus (SARS-CoV-2 Swab)?** | | |
| | 1 - Yes | 48 | 27.3% |
| | 2 - No | 128 | 72.7% |
| v12 | **What was the result?** | | |
| | 1 - Positive | 3 | 1.7% |
| | 2 - Negative | 45 | 25.6% |
| v13 | **How did the media influence your decision making? The** | | |
| | 1 - ... helpful | 81 | 46.0% |
| | 2 - ... confusing | 47 | 26.7% |
| | 3 - I do not rely on the media as an information source. | 25 | 14.2% |
| | 4 - Free text | 23 | 13.1% |
| v14 | **How old are you?** | Mean 50.5 (SD 15), range 18-82 | |
| v15 | **What is your sex?** | | |
| | 1 - Female | 101 | 57.4% |
| | 2 - Male | 75 | 42.6% |
| | 3 - Other | 0 | 0.0% |
| v16 | **What is your nationality?** | | |
| | 0 - Missing | 0 | 0.0% |
| | 1 - Swiss | 147 | 83.5% |
| | 2 - German | 13 | 7.4% |
| | 3 - French | 1 | 0.6% |
| | 4 - Italian | 3 | 1.7% |
| | 5 - Liechtenstein | 0 | 0.0% |
| | 6 - Greater Europe | 4 | 2.3% |
| | 7 - Free text | 7 | 4.0% |
| v17 | **In which province do you live?** | | |
| | 1 - Bern | 108 | 61.4% |
| | 2 - Zürich | 12 | 6.8% |
| | 3 - Luzern | 10 | 5.7% |
| | 4 - Uri | 0 | 0.0% |
| | 5 - Schwyz | 1 | 0.6% |
| | 6 - Obwalden | 0 | 0.0% |
| | 7 - Nidwalden | 0 | 0.0% |
| | 8 - Glarus | 0 | 0.0% |
| | 9 - Zug | 2 | 1.1% |
| | 10 - Fribourg | 7 | 4.0% |
| | 11 - Solothurn | 3 | 1.7% |
| | 12 - Basel-Stadt | 2 | 1.1% |
| | 13 - Basel-Landschaft | 1 | 0.6% |
| | 14 - Schaffhausen | 0 | 0.0% |

For peer review only

| | | | |
|---|---|---|---|
| | 15 - Appenzell Ausserrhoden | 2 | 1.1% |
| | 16 - Appenzell Innerrhoden | 0 | 0.0% |
| | 17 - St. Gallen | 2 | 1.1% |
| | 18 - Graubünden | 3 | 1.7% |
| | 19 - Aargau | 9 | 5.1% |
| | 20 - Thurgau | 1 | 0.6% |
| | 21 - Ticino | 2 | 1.1% |
| | 22 - Vaud | 7 | 4.0% |
| | 23 - Valais | 0 | 0.0% |
| | 24 - Neuchâtel | 1 | 0.6% |
| | 25 - Geneva | 0 | 0.0% |
| | 26 - Jura | 0 | 0.0% |
| | 27 - I do not live in Switzerland | 3 | 1.7% |
| v18 | **What is your highest level of education?** | | |
| | 0 - Missing | 6 | 3.4% |
| | 1 - Tertiary education (university degree, college of education) | 120 | 68.2% |
| | 2 - Upper secondary education (High School Graduation, FMS, EZF, EBA) | 27 | 15.3% |
| | 3 - Lower secondary education/ obligatory schooling completed | 23 | 13.1% |
| | 4 - No formal education | | |
| v19 | **Are you currently...** | | |
| | 0 - Missing | 33 | 18.8% |
| | 1 - Employed | 106 | 60.2% |
| | 2 - Self employed | 24 | 13.6% |
| | 3 - Unemployed already before the current pandemic | 3 | 1.7% |
| | 4 - I lost my job during the COVID-19 period | 1 | 0.6% |
| | 5 - Studying or in an apprenticeship | 9 | 5.1% |
| v20 | **How much approximately do you earn per month? (net income in December 2019 including 1/12 of the 13th month salary.)** | | |
| | 0 - Missing | 29 | 16.5% |
| | 1 - Less that 4'000 CHF | 26 | 14.8% |
| | 2 - Between 4'001 und 6'000 CHF | 42 | 23.9% |
| | 3 - Above 6'001 CHF | 79 | 44.9% |
| v21 | **What type of health insurance do you have?** | | |
| | 1 - General | 68 | 38.6% |
| | 2 - Telemedicine - Modell | 12 | 6.8% |
| | 3 - GP - Modell | 83 | 47.2% |
| | 4 - Another alternative model | 8 | 4.5% |
| | 5 - No insurance | 5 | 2.8% |
| v22 | **In a second stage, we will interview individual participants of** | | |
| | 1 - Yes, I consent to be contacted. | 78 | 44.3% |
| | 2 - No, please, no more interviews. | 98 | 55.7% |
| | 3 - Free text | - | - |

For peer review only

Interview Guide: Coronatest.ch -Patients v 2

# Rapport

0Describe yourself (prompts; nationality, occupation, living arrangements, employment status)

# Accessibility

0How did you get to coronatest.ch website? (referred by …, online search). What is your understanding of an OFTT?

0Did you consult BAG- was the information supplied by BAG understandable to you? In what way was it useful.

0Was the online digital tool easy for you to access? explain why or why not (easy to find on homepage, length of tool, clear and easy to follow instructions, language, sequence, when did you use the tool Monday, Tuesday, weekend or during the week and why)

0What information or components would have helped you better -what do you suggest needs to be done to make such an online too more accessible.

# Utility as a reliable information source and decision making

0When you consulted the online digital tool; did you follow the recommended advice? Prompt (why and how socioeconomic status could have influenced the process)

0What made you follow the advice and recommendations? Prompt confirmation from friends, generally, the media influence your decision-making process?

0What made you disregard the advice and recommendations? (prompt for severity of symptoms, change of condition)

0We have noticed that people that earn below 4000 tend not to follow recommendations, why do you think it`s like this, what can be done?

# Utility in allaying fear and anxiety

0Describe how you felt after consulting the online digital tool with regards to feeling anxious and or confident that all was going to be well? (Did you feel reassured after visiting corontest.ch)

0If your fears were not allayed, how did you deal with your fears? prompt on what increased confidence, what allayed your anxiety)

For peer review only

# Illness and testing Experience

0Did you test for COVID-19, what test and experience? Did you experience COVID-19 symptoms? Explain

0Speaking of self-isolation. Please explain in more detail how you experienced this what worked and what did not work e.g. the need to go shopping, not going for a run or walk protecting others vs own needs, putting others first, dilemmas, challenges

0The road to recovery has been described by many as very cumbersome and long-what was your experience? Any psychiatric or other residual effects experienced-explain.

0What personal life lessons did you learn during this pandemic you would like to share and what personal changes do you foresee in future

0What health and health system related observations did you make and what changes do you fore see in future?

0What, socio-economic changes have you observed and do you foresee in the future as a result of COVID-19

# Utility in preventing cross infection

0If you consulted a GP; did you call ahead of time?

0How did your GP/ health care provider react when you told him or her you suspected that you had COVID-19?

# Recommendations

0In a future pandemic, what would you do?

0Is there any additional information you wish a site like corontest provides but was missing during COVID-19? What information did you search for but did not find?

0Are there other strategies (to allay fear, anxiety) you deem effective alone or in conjunction with online digital tools when faced with epidemics such as COVID-19 to make it accessible to the older generation?

0How can online tools like corontast be adapted to facilitate your decision making processes

For peer review only

STROBE Statement—checklist of items that should be included in reports of observational studies

| | Item No | Recommendation | Page No |
|---|---|---|---|
| **Title and abstract** | 1 | (*a*) Indicate the study's design with a commonly used term in the title or the abstract | 1 |
| | | (*b*) Provide in the abstract an informative and balanced summary of what was done and what was found | 3 |
| **Introduction** | | | |
| Background/rationale | 2 | Explain the scientific background and rationale for the investigation being reported | 5 |
| Objectives | 3 | State specific objectives, including any prespecified hypotheses | 5 |
| **Methods** | | | |
| Study design | 4 | Present key elements of study design early in the paper | 6 |
| Setting | 5 | Describe the setting, locations, and relevant dates, including periods of recruitment, exposure, follow-up, and data collection | 6 |
| Participants | 6 | (*a*) *Cohort study*—Give the eligibility criteria, and the sources and methods of selection of participants. Describe methods of follow-up<br>*Case-control study*—Give the eligibility criteria, and the sources and methods of case ascertainment and control selection. Give the rationale for the choice of cases and controls<br>*Cross-sectional study*—Give the eligibility criteria, and the sources and methods of selection of participants | 7 |
| | | (*b*) *Cohort study*—For matched studies, give matching criteria and number of exposed and unexposed<br>*Case-control study*—For matched studies, give matching criteria and the number of controls per case | |
| Variables | 7 | Clearly define all outcomes, exposures, predictors, potential confounders, and effect modifiers. Give diagnostic criteria, if applicable | 7 |
| Data sources/ measurement | 8* | For each variable of interest, give sources of data and details of methods of assessment (measurement). Describe comparability of assessment methods if there is more than one group | *8-9* |
| Bias | 9 | Describe any efforts to address potential sources of bias | 9 |
| Study size | 10 | Explain how the study size was arrived at | 8 |
| Quantitative variables | 11 | Explain how quantitative variables were handled in the analyses. If applicable, describe which groupings were chosen and why | 10 |
| Statistical methods | 12 | (*a*) Describe all statistical methods, including those used to control for confounding | 10 |
| | | (*b*) Describe any methods used to examine subgroups and interactions | 10 |
| | | (*c*) Explain how missing data were addressed | 10.11.12.13 |
| | | (*d*) *Cohort study*—If applicable, explain how loss to follow-up was addressed<br>*Case-control study*—If applicable, explain how matching of cases and controls was addressed<br>*Cross-sectional study*—If applicable, describe analytical methods taking account of sampling strategy | |

For peer review only

| | |
|---|---|
| (*e*) Describe any sensitivity analyses | 12 |

Continued on next page

For peer review only


For peer review only - http://bmjopen.bmj.com/site/about/guidelines.xhtml

| | | | |
|---|---|---|---|
| **Results** | | | |
| Participants | 13* | (a) Report numbers of individuals at each stage of study—eg numbers potentially eligible, examined for eligibility, confirmed eligible, included in the study, completing follow-up, and analysed | 11 |
| | | (b) Give reasons for non-participation at each stage | |
| | | (c) Consider use of a flow diagram | |
| Descriptive data | 14* | (a) Give characteristics of study participants (eg demographic, clinical, social) and information on exposures and potential confounders | 11 |
| | | (b) Indicate number of participants with missing data for each variable of interest | 11 |
| | | (c) *Cohort study*—Summarise follow-up time (eg, average and total amount) | |
| Outcome data | 15* | *Cohort study*—Report numbers of outcome events or summary measures over time | |
| | | *Case-control study*—Report numbers in each exposure category, or summary measures of exposure | |
| | | *Cross-sectional study*—Report numbers of outcome events or summary measures | |
| Main results | 16 | (*a*) Give unadjusted estimates and, if applicable, confounder-adjusted estimates and their precision (eg, 95% confidence interval). Make clear which confounders were adjusted for and why they were included | 12.13.14 |
| | | (*b*) Report category boundaries when continuous variables were categorized | |
| | | (*c*) If relevant, consider translating estimates of relative risk into absolute risk for a meaningful time period | |
| Other analyses | 17 | Report other analyses done—eg analyses of subgroups and interactions, and sensitivity analyses | |
| **Discussion** | | | |
| Key results | 18 | Summarise key results with reference to study objectives | 18 |
| Limitations | 19 | Discuss limitations of the study, taking into account sources of potential bias or imprecision. Discuss both direction and magnitude of any potential bias | 21 |
| Interpretation | 20 | Give a cautious overall interpretation of results considering objectives, limitations, multiplicity of analyses, results from similar studies, and other relevant evidence | 20 |
| Generalisability | 21 | Discuss the generalisability (external validity) of the study results | 21 |
| **Other information** | | | |
| Funding | 22 | Give the source of funding and the role of the funders for the present study and, if applicable, for the original study on which the present article is based | 24 |

*Give information separately for cases and controls in case-control studies and, if applicable, for exposed and unexposed groups in cohort and cross-sectional studies.

**Note:** An Explanation and Elaboration article discusses each checklist item and gives methodological background and published examples of transparent reporting. The STROBE checklist is best used in conjunction with this article (freely available on the Web sites of PLoS Medicine at http://www.plosmedicine.org/, Annals of Internal Medicine at http://www.annals.org/, and Epidemiology at http://www.epidem.com/). Information on the STROBE Initiative is available at www.strobe-statement.org.

For peer review only
